# Enabling a bioinspired *N*,*N*,*N*-copper coordination motif through spatial control in UiO-67: synthesis and reactivity†

**DOI:** 10.1039/d3dt03096b

**Published:** 2024-03-12

**Authors:** Isabelle Gerz, Erlend S. Aunan, Valeria Finelli, Mouhammad Abu Rasheed, Gabriele Deplano, Rafael Cortez S. P., Inga L. Schmidtke, David S. Wragg, Matteo Signorile, Knut T. Hylland, Elisa Borfecchia, Karl Petter Lillerud, Silvia Bordiga, Unni Olsbye, Mohamed Amedjkouh

**Affiliations:** a Department of Chemistry, University of Oslo P. O. Box 1033 Blindern N-0315 Oslo Norway; b Centre for Materials Science and Nanotechnology, University of Oslo P.O. Box 1126 Blindern N-0316 Oslo Norway; c Department of Chemistry, NIS and INSTM Reference Centre, Università di Torino Via G. Quarello 15/A I-10135 and Via P. Giuria 7 I-10125 Turin Italy; d University School for Advanced Studies IUSS Pavia Palazzo del Broletto Piazza della Vittoria 15 I-27100 Pavia Italy; e Hylleraas Centre for Quantum Molecular Sciences, Department of Chemistry, University of Oslo N-0315 Oslo Norway

## Abstract

Metal–organic frameworks (MOFs) featuring zirconium-based clusters are widely used for the development of functionalized materials due to their exceptional stability. In this study, we report the synthesis of a novel N,N,N-ligand compatible with a biphenyl dicarboxylic acid-based MOF. However, the resulting copper(i) complex exhibited unexpected coordination behaviour, lacking the intended trifold coordination motif. Herein, we demonstrate the successful immobilization of a bioinspired ligand within the MOF, which preserved its crystalline and porous nature while generating a well-defined copper site. Comprehensive spectroscopic analyses, including X-ray absorption, UV/Vis, and infrared spectroscopy, were conducted to investigate the copper site and its thermal behaviour. The immobilized ligand exhibited the desired tridentate coordination to copper, providing access to a coordination motif otherwise unattainable. Notably, water molecules were also found to coordinate to copper. Upon heating, the copper centre within the MOF exhibited reversible dehydration, suggesting facile creation of open coordination sites. Furthermore, the copper site displayed reduction at elevated temperatures and subsequent susceptibility to oxidation by molecular oxygen. Lastly, both the molecular complexes and the MOF were evaluated as catalysts for the oxidation of cyclohexane using hydrogen peroxide. This work highlights the successful immobilization of a bioinspired ligand in a zirconium-based MOF, shedding light on the structural features, thermal behaviour, and catalytic potential of the resulting copper sites.

## Abstract 2

The incorporation of a novel *N*,*N*,*N*-linker into UiO-67 permits it to coordinate copper in an otherwise unattainable way.

## Graphical abstract

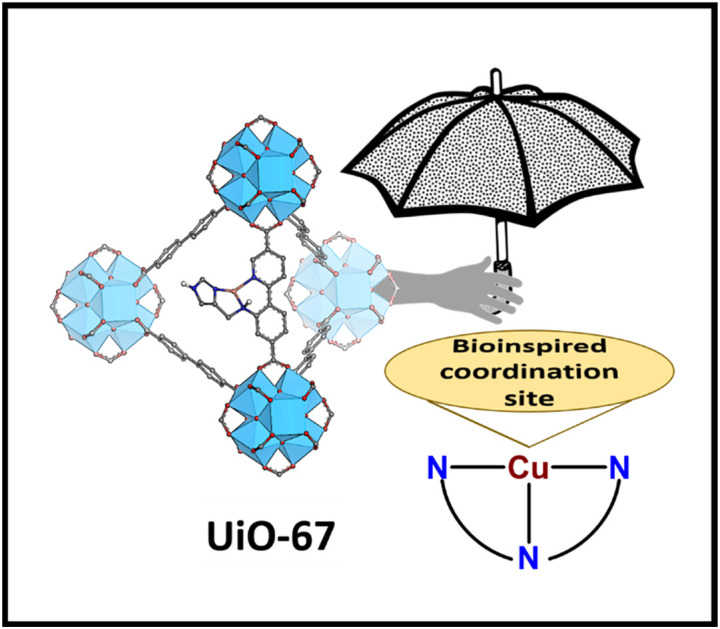

## Introduction

**Fig. 1:**
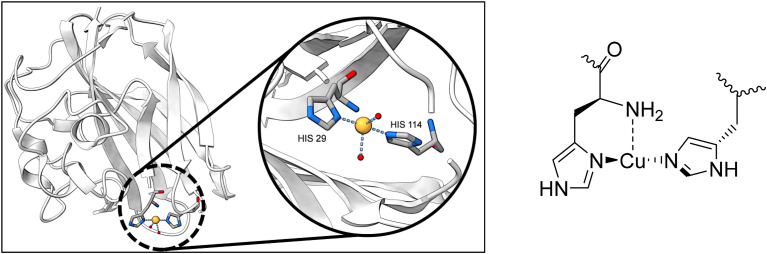
Schematic representation of the histidine copper site, the active site in LPMO. Protein Data Bank ID: 4alc.^10^

Monooxygenases are a class of enzymes that add a single hydroxyl group to their substrate. Both particulate methane monooxygenase (pMMO) and the lytic polysaccharide monooxygenase (LPMO) feature a structural motif known as a histidine brace, coordinating a copper atom (see Fig. 1).^1–5^ The T-shaped, tridentate coordination of copper by the histidine brace has been identified in the literature as key for LPMO's reactivity.^6^ There are extensive reports of mimics of the copper histidine brace.^7–9^ However, it lies in the nature of the open coordination site created by the T-shape that additional ligands may be coordinated – especially in the copper(ii) state. Commonly, counter-anions are found to coordinate to the copper atom.^8,9^

When weakly coordinating ions are employed, the open coordination site(s) can favor ML_2_ type complexes, hampering efforts to create molecular models that resemble enzymatic active sites.^11,12^ For bulky substituents, ML_2_ formation has been linked to inertia towards oxygen.^13,14^ Therefore, considerable efforts have been directed at controlling the ML *vs.* ML_2_ formation.^14,15^ The well-defined environment in the macromolecular enzyme can be difficult to recreate in a molecular system. Some phenomena observed in molecular active site mimics, such as aggregation,^16–19^ disproportionation^20–22^ and formation of oxo-bridged dimers (as opposed to reactive mono-metallic sites)^23^ can also be traced back to the lack of spatial separation of the metal complexes. The attachment to a framework can enforce spatial separation, preventing ML_2_ formation and the aforementioned phenomena. For example, Nash *et al.* resolved the aggregation tendencies of Zn phthalocyanines under aqueous conditions by incorporation into a metal organic framework. The resulting material showed enhanced singlet oxygen generation compared to the molecular complex.^24^ While the fixation of porphyrins to metal–organic frameworks as heme-enzyme mimics is an active field of research,^25,26^ reports of metal–organic frameworks inspired by the copper-histidine motif are scarce. Yaghi's group presented a bioinspired complex in MOF-808, by functionalizing the cluster with histidines.^27^ We recently incorporated a bis-imidazole copper complex into a metal–organic framework by post-synthetic covalent functionalization of the linker.^19^ However, neither Yaghi's nor our structures had the T-shaped *N*,*N*,*N*-coordination around copper. Herein, we present such a three-coordinate environment embedded into UiO-67, a zirconium MOF renowned for its stability.^28–31^ The coordination environment of copper in the MOF was studied by a combination of spectroscopic methods (UV/Vis, IR, XAS) at different temperatures, revealing a CuL complex that is reversibly hydrated and, once reduced, shows modest reactivity towards oxygen. Lastly, the MOF and the molecular complexes were tested as catalysts for C–H activation of cyclohexane.

## Results and discussion

### Ambition and synthetic considerations

**Fig. 2:**
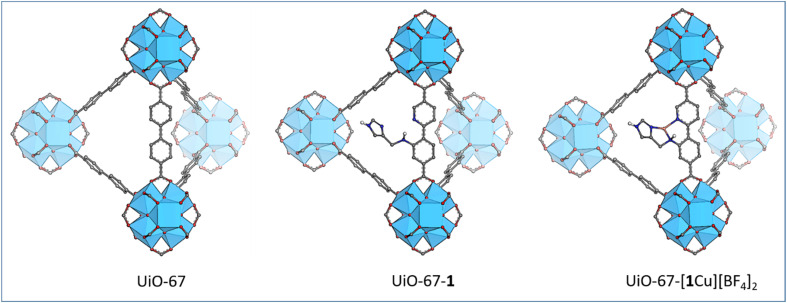
Sketch of the target MOFs in this work. Starting from the standard framework UiO-67 with biphenyl dicarboxylic acid linkers (left), 1 can be introduced through PSLE (middle) and metallated to yield UiO-67 with the bioinspired *N*,*N*,*N*-coordination motif around Cu (right). Zirconium: blue polyhedra, oxygen: red, carbon: grey, nitrogen: blue, copper: orange. Hydrogen: white. Non-heteroatomic hydrogen and the counterion are omitted for clarity.

The functionalization of a MOF with a bioinspired moiety requires careful planning of the synthetic steps. The two aryl rings in the linker of UiO-67 were identified as a suitable scaffold for the *N*,*N*,*N*-coordination motif. Fig. 2 depicts the aimed-at functionalized MOFs along with the parent MOF UiO-67, visualizing the synthetic goals of this work. The strategy stipulates to first synthesize the novel linker 1 that is then introduced to UiO-67 by post-synthetic linker exchange (PSLE) to form the mixed linker material UiO-67-1. In the target linker, one aryl ring of the standard biphenyl linker is replaced with a pyridine while the other is substituted with a secondary amine that bears an imidazole. Thereby, the linker structure necessary for the MOF topology is retained, while providing the coordination environment for Cu.

In this work, the material synthesis will be described, followed by spectroscopic investigations that seek to identify the coordination environment of copper and finally, the products’ catalytic activity towards cyclohexane is studied.

### Synthesis of the novel linker H_2_1

**Scheme 1:**
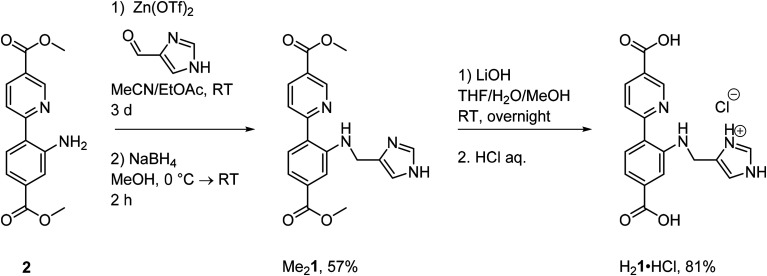
Synthesis of the functionalized linker H_2_1·HCl.

Secondary amines are readily obtained from the corresponding imines through reductive amination (Scheme 1, all procedures and characterization for the linker synthesis can be found in section 1.1 in the ESI; Fig. S1–13 and Tables S1, 2†). This can be performed either directly or indirectly, meaning that the imine is either generated *in situ* and reduced immediately or isolated first and then reduced in an additional step. Since the imine could not be isolated, the indirect approach could not be applied as a synthesis route in this instance. The direct approach gave the desired product, albeit in very poor yields. The imine could however be stabilized through zinc coordination. Elemental analysis of the synthetic intermediate was consistent with one imine ligand per zinc triflate, formulating the complex as ZnL. The intermediate decomposed rapidly in solution (see Fig. S4†). Subsequent treatment with NaBH_4_ in MeOH, followed by aq. Na_2_EDTA solution, furnished the desired amine ligand Me_2_1 in good yields. Me_2_1, an intensely yellow colored solid, was characterized by NMR, HRMS, elemental analysis, UV/Vis and SC-XRD. The corresponding diacid H_2_1, capable of acting as a linker in the UiO-67 MOF, was obtained as its HCl salt through ester hydrolysis of Me_2_1.

### Syntheses of the molecular Cu(i) and Cu(ii) complexes of Me_2_1

**Scheme 2:**
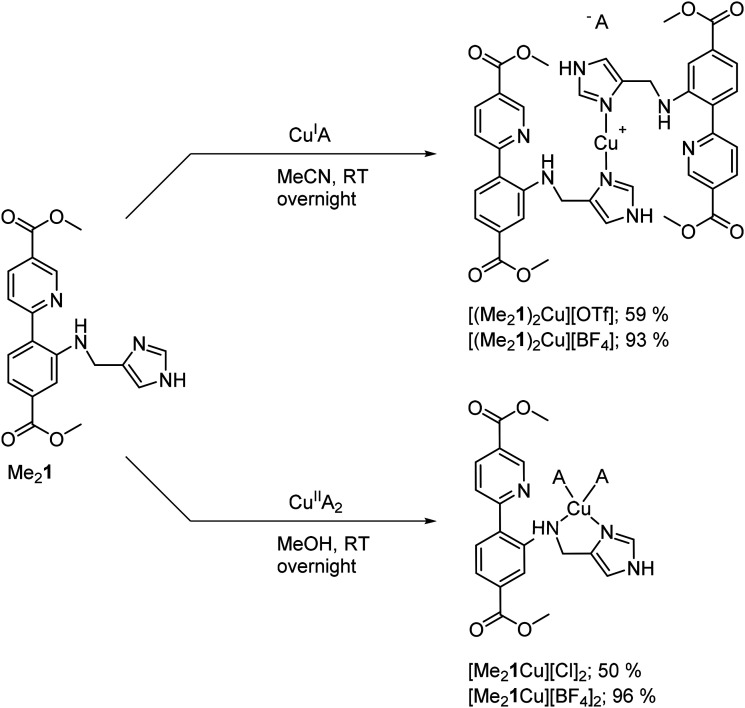
Molecular copper complexes synthesized in this work.

Molecular Cu complexes were synthesized from Me_2_1, both as Cu(i) and as Cu(ii) complexes (Scheme 2, all procedures and characterization for the complex synthesis can be found in section 1.2 in the ESI; Fig. S14–22 and Tables S3†) as references for the spectroscopic and catalytic studies. The methyl ester bearing compounds were chosen over the free acid to ensure solubility in MeCN. Reacting Me_2_1 with CuOTf afforded the homoleptic complex [(Me_2_1)_2_Cu][OTf]. The copper(i) complex had a similar color to its ligand and was characterized by NMR, HRMS, elemental analysis, and UV/Vis (see ESI†). ^15^N NMR studies found a coordination shift Δ*δ*^15^N of −53.0 ppm for the imidazole nitrogen, which is in good agreement with the coordination shifts we reported for other imidazole copper(i) complexes.^19^ In contrast, the pyridine shift barely changed upon metalation (Δ*δ*^15^N = –3.8 ppm), indicating little interaction with copper. The amine nitrogen could not be detected in the ^1^H-^15^N HMBC spectrum of the complex.

Altering the copper source from CuOTf to CuBF_4_ did not only improve the yield considerably (Scheme 2), but it also allowed for analysis by SC-XRD, which proved difficult before despite employing different crystallization techniques. It is worth noting, that the NMR data was unaffected by the exchange of the counter ion, suggesting a high similarity of the structures in solution. The SC-XRD structure of [(Me_2_1)_2_Cu][BF_4_] (see Fig. S20 and Table S3†) could be obtained after recrystallization from acetonitrile. The crystal scattered weakly, and the counter ion was disordered, resulting in a low-resolution crystal structure. Nevertheless, the structure revealed a linear copper(i) complex, where copper is solely ligated by the imidazole moieties of the two ligands. The linear coordination motif is underpinned by the high similarity of the Cu K-edge XANES of [Cu(Me_2_1)_2_][OTf] with that of a [Cu(NH_3_)_2_]^+^ model compound, measured in solution^32^ (see Fig. S21†), where both compounds show the prominent rising edge peak at *ca.* 8983 eV, fingerprinting linear Cu(i) sites.^33,34^

The Cu(ii) complexes [Me_2_1Cu][BF_4_]_2_ and [Me_2_1Cu][Cl]_2_ were synthesized from Me_2_1 and Cu(BF_4_)_2_·6H_2_O and CuCl_2_ respectively. The Cu(ii) complexes were characterized by elemental analysis.

### Syntheses of the functionalized MOFs

**Fig. 3:**
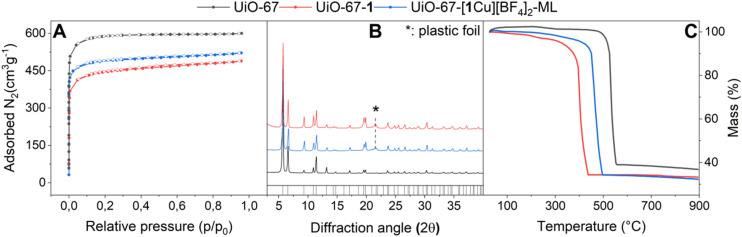
Basic characterization of UiO-67 (black), UiO-67-1 (blue) and UiO-67-[1Cu][BF_4_]_2_-ML (red). (A) Nitrogen adsorption isotherms performed at 77 K. (B) Measured powder X-ray diffractograms (*Y*-axis: normalized intensity), with calculated reflections given in the rug. (C) Thermogravimetric analysis (30 °C − 900 °C) performed in synthetic air (100 mLmin-1).

H_2_1 was incorporated into UiO-67 *via* post-synthetic linker exchange (PSLE) furnishing UiO-67-1. The functionalized MOF was metallated using a solution of Cu(BF_4_)_2_·6H_2_O in acetonitrile, yielding UiO-67-[1Cu][BF_4_]_2_ as a green powder. The metalation was performed in a second step in order to ensure that copper is preferentially situated at incorporated ligand sites and to prevent formation of CuL_2_ species. UiO-67-[1Cu][BF_4_]_2_ was synthesized with various loadings of both 1 as well as Cu/L-ratios in order to maximize the information about the local copper environment gained by various spectroscopic characterization techniques. An overview of the synthesized MOFs is given in Table 1. The basic characterization of UiO-67-[1Cu][BF_4_]_2_-ML is shown in Fig. 3. All synthetic procedures and detailed characterization data can be found in section 1.3 in the SI (Fig. S23–34 and Tables S4–8†). Despite a slight loss of surface area (5–35%) associated with the post-synthetic modifications, the final materials were obtained as highly porous solids with retained crystallinity and they displayed good thermal stability.

**Table 1:** Overview over the three copper incorporated materials, where LL, ML and HL refers to low, medium, and high loading of Cu, respectively

Material	1/Zr_6_	Cu/Zr_6_	Cu/1
UiO-67-[1Cu][BF_4_]_2_-LL	0.3	0.2	0.67
UiO-67-[1Cu][BF_4_]_2_-ML	1.2	0.8	0.67
UiO-67-[1Cu][BF_4_]_2_-HL	1.2	1.2	1.00

### Spectroscopic characterization of the MOFs

**Fig. 4:**
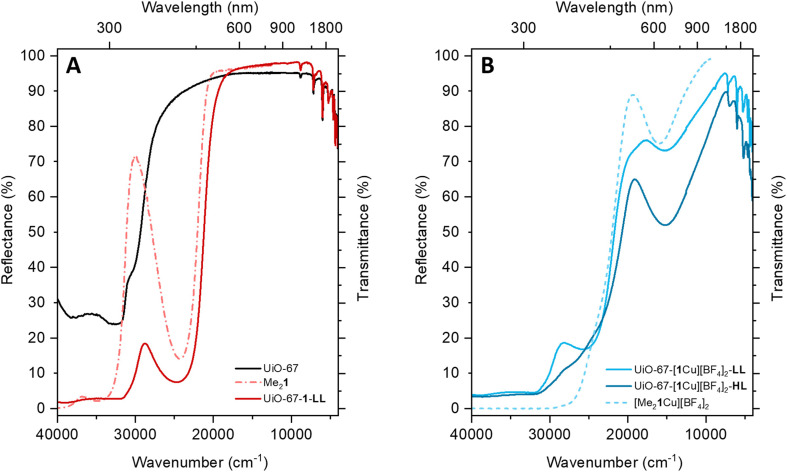
Comparison of UV/Vis spectra of the MOFs (measured in DR) and the molecular compounds (measured in T, MeCN solutions). (A) The parent MOF UiO-67 (solid black curve) is compared to the ligand-exchanged UiO-67-1-LL (solid red curve), which exhibits the characteristic band causing the yellow color of the ligand Me_2_1 (dashed red curve). (B) The MOF (LL and HL, solid light blue and dark blue curve) is compared to the molecular copper complex [Me_2_1Cu][BF_4_]_2_ (dashed light blue curve).

The incorporation of ligand 1 and its subsequent metalation result in color changes visible to the naked eye (colorless to yellow to green). The diffuse reflectance (DR) UV/Vis measurements can be compared to the transmission spectra of the respective molecular compounds in solution (Fig. 4).

**Fig. 5:**
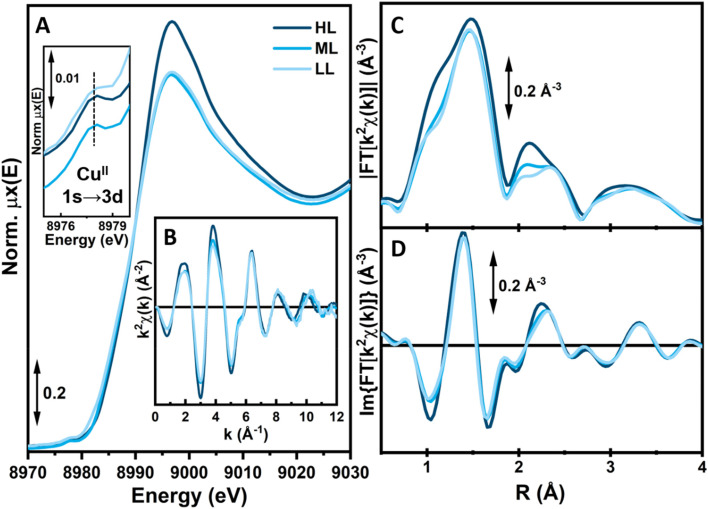
Overview of Cu K-edge XAS results for as-prepared UiO-67-[1Cu][BF_4_]_2_ with different Cu-loading (high loading, HL: black; medium loading, ML: grey; low loading, LL: light grey curves) measured at RT. (A) XANES spectra; the inset reports a magnification of the pre-edge peak assigned to 1s → 3d in Cu(ii). Centers. (B) *k*^2^-weighted *χ*(*k*) EXAFS spectra. (C and D) Magnitude (C) and imaginary part (D) of the FT-EXAFS spectra, obtained transforming the *k*^2^*χ*(*k*) curves in part (B) in the 2.5–11.0 Å^−1^ range.

The spectrum of UiO-67 (Fig. 4A, black curve) is characterized by a strong absorption in the UV region (starting from 30 000 cm^−1^) ascribed to π–π* transition of the biphenyl dicarboxylate (BPDC) linker.^35^ The inclusion of 1 is testified by the appearance of a new component at 24 600 cm^−1^ (Fig. 4A, red curve). The assignment is confirmed by the presence of a similar component in the spectrum of Me_2_1 in MeCN (dashed red curve). Due to partial substitution of the linker, the UV/Vis spectrum of UiO-67-[1Cu][BF_4_]_2_-LL combines the spectral features of UiO-67 and H_2_1. However, in UiO-67-[1Cu][BF_4_]_2_-LL, the very high intensity of the absorption bands above 30 000 cm^−1^, does not allow to distinguish the shoulder at 30 000 cm^−1^, visible in UiO-67. The subsequent incorporation of copper as a Cu(ii) species is testified by the appearance of a new broad and tailed band at 15 100 cm^−1^, in the region typical of d–d transitions for the metal cation (Fig. 4B).^36^ The d–d-transition in the MOFs is red-shifted compared to the one in [Me_2_1Cu][BF_4_]_2_ (dashed light blue line), suggesting subtle changes in the local copper environment. While these spectra confirm the successful copper incorporation, they offer little information about the precise coordination environment around copper. The element-selective response and the combined sensitivity to both oxidation state and local structure around Cu atoms^4,5^ make XAS the technique of choice to address these aspects, as proven by a number of previous XAS-based works targeting the redox chemistry of Cu ions in both MOFs^6,7^ and zeolites.^8–10^ The as-prepared materials HL, ML and LL exclusively contain Cu(ii) centers, showing the characteristic XANES features of six/five-fold coordinated sites,^11,12^*i.e.*, a smooth rising-edge profile and an intense white-line peak at *ca.* 8996 eV (Fig. 5 and section S2, Fig. S35 and Table S9†). Based on these observations, pointing to a well-defined oxidation state of Cu species in as-prepared UiO-67-[1Cu][BF_4_]_2_-HL, we fitted the corresponding EXAFS spectrum. EXAFS analysis supported the presence of five-fold coordinated Cu(ii) sites, bonded to the three light atoms of linker 1 (〈*R*_N_〉 = 1.92 ± 0.02 Å) and to two additional O atoms (〈*R*_O_〉 = 2.02 ± 0.02 Å), plausibly from water/hydroxyl extra-ligands. The accuracy of XAS to identify light atoms is insufficient to distinguish nitrogen from oxygen ligands,^34,37,38^ therefore the data could also support different distributions of nitrogen and oxygen across the two observed bond lengths. At higher R, the EXAFS signal is consistent with the coordination environment offered by the functionalized linker, including contributions from the six closest C atoms in the second-shell region (〈*R*_C_〉 = 2.88 ± 0.04 Å).

For comparison purposes, UiO-67-[1Cu][BF_4_]_2_-LL and -ML samples (RT, in air) were characterized by *ex situ* XAS (Fig. 5, light blue curves). A lower Cu loading appears to promote a decrease in the average first-shell coordination number. This is indicated by both a less intense white line peak in the XANES and a lower first-shell peak in the FT-EXAFS, observed to the same extent in both ML and LL samples with respect to the HL one. Importantly, from *ca.* 2.2 Å upward, all the three materials show a very similar EXAFS signal, supporting in all cases an equivalent coordination motif of Cu towards linker 1. As also supported by EXAFS fitting for as-prepared UiO-67-[1Cu][BF_4_]_2_-ML, we instead suggest an influence of the Cu-loading on the average number of H_2_O/OH extra-ligands present in the first coordination sphere of Cu at ambient conditions. In particular, XAS indicates that UiO-67-[1Cu][BF_4_]_2_-ML and -LL could preferentially contain four-fold coordinated Cu(ii) centers, ligated to only one H_2_O/OH molecule in addition to the three N from linker 1. Concluding, XAS analysis could confirm that the synthetic goal of incorporating a *N*,*N*,*N*-coordinated copper moiety (as depicted in Fig. 2) was achieved.

### MOF reactivity

**Fig. 6:**
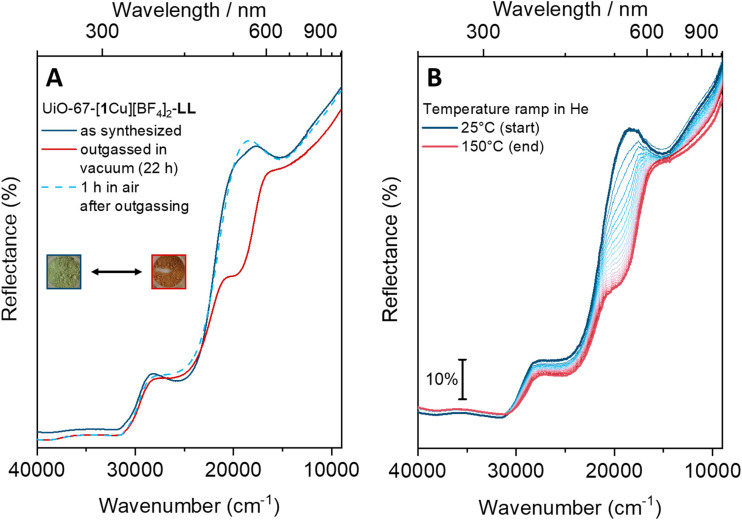
Dehydration of UiO-67-[1Cu][BF_4_]_2_-LL. (A) Outgassing the MOF in vacuum results in an additional, prominent shoulder at 19 500 cm^−1^. The process is reversible and after one hour in ambient atmosphere, the spectral appearance of the pristine MOF is regained. The insets show pictures of the MOF in its hydrated (green) and dehydrated state (brown). (B) Similar spectral changes are observed when the sample is heated in helium.

The MOFs quickly changed color from light green to light brown upon outgassing or heating (see Fig. 6). A similar color change is well-known from the dehydration of hydrated CuCl_2_.^39^ The MOFs’ dehydration/hydration process was reversible, as they regained their green color when left in air. The LL material was chosen for the UV/Vis studies due to the well-defined bands in its spectrum. The color-change stems from an intense shoulder forming at around 19 500 cm^−1^ (Fig. 6A and B, red curves). The less intense, broad d–d band around 15 000 cm^−1^ persists during the process. The brown color, often associated with Cu(i) complexes, is thus not indicative of reduction but rather of a change in coordination environment of the Cu(ii) species. After one hour in air, the ‘activated’ MOF has almost completely recovered pre-treatment features. The process could be accelerated by saturating the air with H_2_O vapor. In contrast, a sample left in a closed vial (still under air) remained light brown. This behavior was not observed for the molecular complex [Me_2_1Cu][BF_4_]_2_ (as a neat solid). When [Me_2_1Cu][BF_4_]_2_ was heated stepwise (80 °C, 120 °C and 150 °C, 1 h at each temperature step) in an oven, the sample's appearance remained unchanged (see Fig. S22†).

Overall, the experimental data suggest that the as-synthesized UiO-67-[1Cu][BF_4_]_2_ has water ligated to copper; this additional ligand seems to be loosely bound, as it is readily removed by heating the sample. The anion, BF_4_^−^, is known to be reactive towards some transition metal complexes.^40^ The reversibility however suggests that if BF_4_^−^ is actively involved, it is not consumed.

The higher Cu-loading in UiO-67-[1Cu][BF_4_]_2_-HL guarantees the best data quality under *in situ* conditions, allowing to monitor the EXAFS region at the key treatment steps, comparing the material in its as-prepared state at RT in He, after thermal treatment in He up to 150 °C and upon subsequent exposure to O_2_, still at 150 °C.

**Fig. 7:**
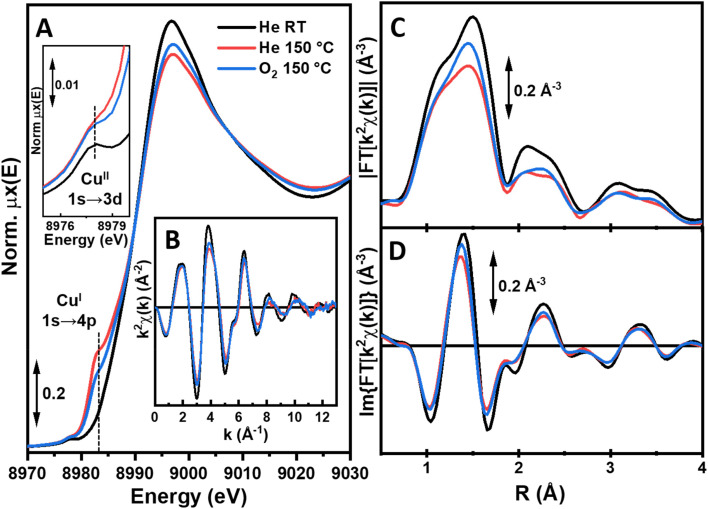
Overview of *in situ* Cu K-edge XAS results for UiO-67-[1Cu][BF_4_]_2_-HL at key treatment steps. (A) XANES spectra; the inset reports a magnification of the pre-edge peak assigned to 1s → 3d transition in Cu(ii) centers; the rising-edge peak assigned to 1s → 4p transition in Cu(i) centers is also labelled in the main panel. (B) *k*^2^-weighted *χ*(*k*) EXAFS spectra. (C and D) Magnitude (C) and imaginary part (D) of the FT-EXAFS spectra, obtained transforming the *k*^2^*χ*(*k*) curves in part (B) in the 2.5–12.0 Å^−1^ range.

The XANES features for UiO-67-[1Cu][BF_4_]_2_-HL after thermal treatment up to 150 °C (Fig. 7, red curves) clearly evidence a partial reduction to Cu(i), giving a mixed Cu(ii)/Cu(i) state. Indeed, while the pre-edge at *ca.* 8977 eV peak diagnostic for Cu(ii) is still detectable, a rising-edge peak at *ca.* 8983 eV develops, fingerprinting the simultaneous presence of Cu(i) centers. In contrast to the peak observed for linear Cu(i) species in [Cu(Me_2_1)_2_][OTf], sharp and well separated from the main edge (see Fig. S21†), this peak is quite broad and occurs rather as a rising edge shoulder. These spectroscopic observations suggest three-fold coordinated Cu(i) sites to form in the MOF scaffold under *in situ* conditions. In the EXAFS, we observe a substantial decrease in the first-shell peak with respect to what was observed at RT, arising due to both thermal dampening effects and dehydration/partial reduction phenomena involving the Cu centers. Notably, the EXAFS signal in the range of 2.3–4.0 Å is homogeneously dampened, as expected due to thermal effects. Nonetheless, it maintains the same shape and *R*-space localization, supporting an unchanged coordination of Cu centers to the functionalized linker 1. Upon subsequent exposure to O_2_ at 150 °C (Fig. 7, blue curves), a fraction of the Cu(i) centers formed during thermal treatment undergoes re-oxidation to Cu(ii). This behavior is supported by an inverse spectral evolution of the XANES features with respect to what was observed upon heating from RT to 150 °C, including increase of the Cu(ii) pre-edge peak intensity and decrease of the rising-edge peak characteristic of Cu(i). As these two EXAFS spectra were collected under isothermal conditions, it is possible to safely attribute the increase in the first-shell peak observed upon exposure to O_2_ with respect to the previous step at 150 °C in He to an increased coordination number. More likely, this evidence is related to the newly formed Cu(ii) species and it supports an oxidation event accompanied by coordination of O_2_-derived ligands to Cu centers. The XAS element-averaged response together with the relatively low abundance of Cu sites undergoing redox transformation under the conditions employed here hinders the possibility of quantitatively refining local structures from the available data (the effect of O_2_ in a parallel UV/Vis study was also subtle, section S3, Fig. S36 and 37†). Yet, *in situ* XAS unequivocally prove the possibility to form Cu(i) centers in UiO-67-[1Cu][BF_4_]_2_ upon thermal treatment under mild conditions. At least a fraction of the Cu(i) centers is accessible and reactive towards molecular O_2_ at 150 °C, plausibly leading to Cu(ii)-active oxygen species of potential interest for selective oxidation reactions.

**Fig. 8:**
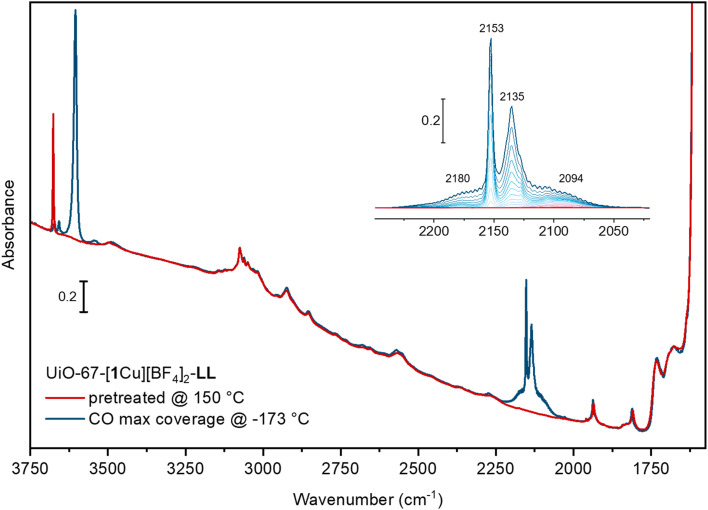
*In situ* IR spectra of UiO-67-[1Cu][BF_4_]_2_-LL after the thermal activation (red curve), and after reaching CO maximum coverage at LT (dark blue curve). The inset shows the effect of intermediates CO coverages where the spectrum of the activated material has been subtracted: maximum coverage (dark blue curve) to complete outgassing (red curve), while intermediate coverages are shown in transient colours.

An indirect method for probing qualitatively open metal sites accessibility from the gas phase is to perform a series of IR spectra collected in presence of increasing CO coverages, (isothermal at low temperature = LT, approx. −173 °C) on the samples pre-treated at 150 °C in dynamic vacuum, monitoring the CO band shift with respect to the gas phase.^41^ The IR spectrum of the pre-treated UiO-67-[1Cu][BF_4_]_2_-LL sample (red curve in Fig. 8) is characterized by an intense band at 3676 cm^−1^, associated to the *ν*(O–H) stretching of isolated μ_3_-OH species on the cornerstones of the Zr_6_-cluster of the MOFs.^35,42–44^ The bands at 3150–3000 cm^−1^ are characteristic of aromatic *ν*(C–H) vibrations, while the ones in the range 2950–2830 cm^−1^ refer to aliphatic *ν*(C–H) peculiar of residual DMF acting as a linker to the Zr_6_-cluster in case of missing linker defects.^42^ Unfortunately, no clear evidence of any bands involving the imidazole building block is appreciated. Upon CO adsorption at low temperatures (blue curve refers to the maximum coverage) a complete erosion of the μ_3_-OH is observed with the parallel growth of a reversible band at 3605 cm^−1^.^35,44^ The Δ*ν* observed testify the medium-weak strength of the OH–CO interaction, associated with the modest acidity of the hydroxyls at the nodes. The formation of these adducts are associated with the growth of a main peak at 2153 cm^−1^, more intense than the band due to physisorbed CO (2134 cm^−1^). The inset in the figure illustrates the spectra evolution along a progressive decrease in CO equilibrium pressure. Apart from the sharp band at 2153 cm^−1^ and the component at 2134 cm^−1^, two weak bands at 2094 and at 2180 cm^−1^ appear more visible at medium low-coverages. The band at 2094 cm^−1^ and its smaller shoulder at 2105 cm^−1^ can be related to Cu(i)–CO adducts, where the band *ν*(CO) is shifted to lower wavenumbers with respect to the 
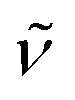
 of gas-phase CO (2143 cm^−1^), as a consequence of a combined effect of CO polarization and π-backdonation from the metal cation to CO antibonding orbitals. This component is the most resistant towards outgassing, inferring a superior stability of the Cu(i)–CO adduct compared to other carbonyls observed here. Finally, the band observed at 2180 cm^−1^ is assigned to the formation of Cu(ii)–CO adducts, as in this case the interaction is dominated by polarization of the probe molecule interacting with the cation.^43,45^ The low intensities of both the components associated with the formation of Cu(i)–CO and Cu(ii)–CO adducts suggest that in the adopted conditions the amount of accessible copper sites with coordinative vacancies are very limited.

### Catalytic testing

**Fig. 9:**
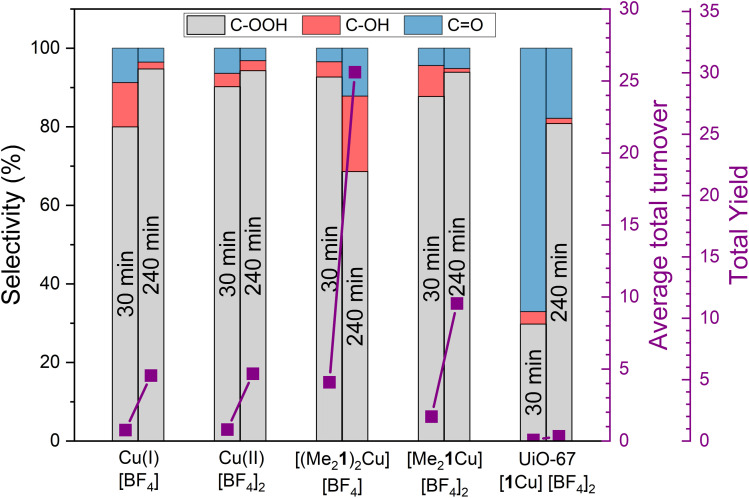
Reactivity of complexes [(Me_2_1)2Cu][BF4] and [Me_2_1Cu][BF_4_]_2_ and corresponding Cu(ii)–MOF system UiO-67-[1Cu][BF_4_]_2_-ML compared to the Cu(i) and Cu(ii) tetrafluoroborate salts for the oxidative conversion of cyclohexane. The graph represents an average of three replicates. C-based product selectivity (left-*Y* axis) and average turnover (purple line, right-*Y* axis) of the different catalysts towards oxidation products data (Cy–OOH + Cy–OH + Cy=O) at 30, and 240 min are shown as pairs of stacked bars from left to right.

The Cu complexes [(Me_2_1)_2_Cu][BF_4_], [Me_2_1Cu][BF_4_]_2_ and the corresponding Cu(ii)–MOF system UiO-67-[1Cu][BF_4_]_2_-ML were tested for the oxidation of cyclohexane with H_2_O_2_ in acetonitrile. The main findings are presented in Fig. 9, together with test results for Cu(i) and Cu(ii) tetrafluoroborate salts [(Me_2_1)_2_Cu][BF_4_] and [Me_2_1Cu][BF_4_]_2_ as reference. The testing procedure and other experimental details are listed in section S4 in the ESI (Fig. S38, 39 and Table S10†). Both Cu complexes and the Cu tetrafluoroborate salts yielded multiple turn-over numbers, indicating that a catalytic reaction took place. No product formation was observed in the absence of Cu. The volatility of cyclohexane led to poor mass balance after the work-up procedure of standard experiments. Therefore, parallel testing of the Cu(ii) complex [Me_2_1Cu][BF_4_]_2_ was performed in a closed NMR tube with *in situ* NMR analysis. The mass balances, taking into account cyclohexane as well as (C/O)–OH and C=O containing products, remained at 98 ± 3% during the first 4 hours of reaction. At longer reaction times, the mass balance gradually decreased due to the formation of over-oxidation products, which were too disperse and low in abundance to be quantified or assigned to specific products (Fig. S38†). Hence, only the results obtained during the first 4 hours are reported here. Another reason for discarding the test results obtained at longer reaction time is precipitate formation, observed as an increasing cloudiness of solution. For the Cu(i) and Cu(ii) complexes, precipitation started after ∼4 hours reaction time. For the Cu tetrafluoroborate salts, cloudiness was observed earlier, after ∼1.5, and ∼3 h reaction time for Cu(i)[BF_4_] and Cu(ii)[BF_4_]_2_, respectively. These observations suggest that the ligand stabilizes a solvated Cu species, even though catalyst degradation through precipitate formation eventually occurs for both, triflate salt and complex.

The results presented in Fig. 9 show that both Cu(i) and Cu(ii) complexes have substantially higher reactivity compared to the corresponding Cu(i) and Cu(ii) salts. Moreover, this difference is more pronounced for the Cu(i) pair than the Cu(ii) pair, and similar activity was observed for the Cu tetrafluoroborate salts of either oxidation state. Furthermore, the turn-over frequency of UiO-67-[1Cu][BF_4_]_2_-ML was more than an order of magnitude lower than that of the corresponding Cu(ii) complex. Product selectivity data are presented in Fig. 9: For all catalysts (except the MOF at 30 min reaction time), cyclohexyl hydroperoxide is the main product, followed by cyclohexanone and cyclohexanol. No other products were detected by GC-MS.

**Fig. 10:**
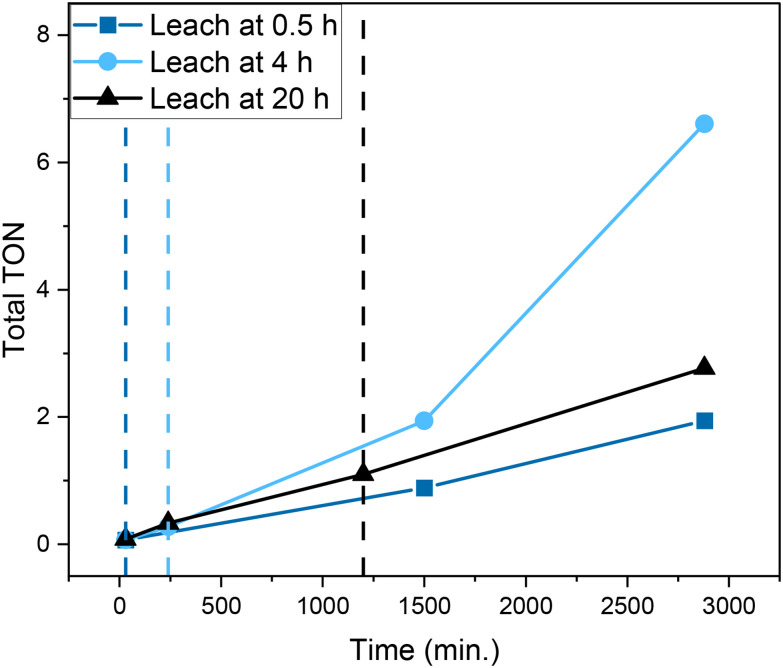
Leaching test data for UiO-67-[1Cu][BF_4_]_2_. MOF separated from solution at 0.5 h (dark blue line), at 4 h (light blue line), and at 20 h (black line) contact time and left reacting until 48 h total reaction time.

The poor performance of UiO-67-[1Cu][BF_4_]_2_ led us to perform leaching tests. The MOF catalyst was removed after 0.5, 4 and 20 h from solution, and the reaction was monitored for 24 h (as reported in Fig. 10). For all three experiments, the reaction progressed despite removal of the MOF. As can be seen, removing the MOF after 0.5 h led to lower product formation rates than MOF removal after 4 h reaction time. More notably, removing the MOF after 4 h reaction time led to higher overall TON after 48 h than removing it after 20 h reaction time. The MOF remained green after 0.5 and 4 h of reaction, then became yellow after 5–6 h of reaction, remaining so until the end of the test. Additionally, a light-yellow color of the supernatant was observed when separating it from the MOF (visible after 4 and 20 h of reaction, but not after 0.5 h), suggesting that linker was leaching into solution. These results suggest that Cu in solution, rather than Cu in the MOF, is the main catalytic species responsible for cyclohexane oxidation. It further suggests that (at least) two parameters are controlling the catalytic system. One is the amount of copper leaching out of the MOF, which increases with reaction time (0.5 h compared to 4 h and 20 h). The other parameter is related to the amount of hydrogen peroxide available, which decreases more quickly over time when the MOF is present. Indeed, gas evolution indicative of H_2_O_2_ conversion was vivid over the MOF, even though cyclohexane conversion was very low.

Overall, the leaching tests suggest that a catalytic reaction with substantial rate is taking place between UiO-67-[1Cu][BF_4_]_2_ and the co-substrate (H_2_O_2_), but that the rate of cyclohexane oxidation is negligible over the MOF compared to Cu in solution. The reason could be, *e.g.*, hindered access of cyclohexane to the Cu sites (site poisoning or diffusion limitations leading to pore blocking) or competing substrates (*e.g.*, linker components). To elucidate whether cyclohexane or its reaction products are trapped in the MOF, samples of UiO-67-[1Cu][BF_4_]_2_ before and after testing were digested and the solutions were analyzed by NMR. The results are presented in Fig. S39.† Indeed, some interesting features were observed. Firstly, peaks attributed to cyclohexane (1.75 ppm) and cyclohexanol/cyclohexyl hydroperoxide (1.82 ppm) were observed by ^1^H NMR of the reacted sample (Fig. S39,† right inset). The amount was modest, appx. 5 mol% relative to the functionalized linker. However, it might indicate that restricted pore diffusion due to occluded reactant/products could reduce access to the copper sites. Secondly, both ^1^H NMR and ^13^C NMR suggested the formation of new carbonyl species under reaction conditions. This is another indication that additional oxidation products are being formed from cyclohexane (*e.g.*, aldehyde or carboxylic acids). Lastly, some features of ^1^H NMR in the reacted sample (7.1–7.3 ppm) strongly suggested an oxidation of the tridentate ligand, probably on the N–C bond, to form imine species, which are susceptible to hydrolysis to form an aldehyde and amine.^46,47^ This hypothesis was validated by comparing the NMR spectrum of the reacted catalyst with that of 2 (Fig. S39,† left inset). It is challenging to distinguish whether the hydrolysis is taking place under reaction conditions or during digestion from these data. However, the absence of hydrolysis in the fresh UiO-67-[1Cu][BF_4_]_2_ strongly suggests that ligand oxidation took place during testing.

Considering prior literature, the groups of Kirillov/Pombeiro and Garcia-Bosch reported similar results to ours over mono-Cu complexes with N-bound ligands, reacted with H_2_O_2_ in acetonitrile solution, albeit at slightly different conditions.^14,48,49^ Notably, both groups found that cyclohexyl hydroperoxide was the main product, followed by cyclohexanol and cyclohexanone, suggesting a radical reaction mechanism. This conclusion is in line with current mechanistic proposals for the oxidative cleavage of polysaccharide chains by LPMO enzymes, although the rate of reaction is much higher in the enzymatic systems, which are active in an aqueous environment and supported by a huge backbone compared to the Cu complexes.^50,51^

## Conclusion

A new linker for UiO-67 doubling as a metal-coordinating ligand bearing three chemically distinct nitrogen-donors (imidazole, pyridine, and secondary amine) was synthesized. A combined UV/Vis, XAS and IR investigation of UiO-67-[1Cu][BF_4_]_2_ showed that the coordination motif of this ligand towards copper is fundamentally different inside of the MOF to its molecular counterpart. This is attributed to the spatial separation of the ligands when being part of the framework. It consequently prevents the formation of CuL_2_ complexes, which were found for the metalation of the molecular ligand. The spectroscopic data (UV/Vis-NIR and XAS) suggests that the Cu(ii) site in the MOF features labile water ligands. The MOF can be reversibly dehydrated, allowing to open one or more coordination sites. At mild temperatures, the Cu(ii) site in the MOF is partially reduced *via* thermal reduction. Notably, the thereby created reduced species is different from the molecular Cu(i) species, highlighting that the incorporation into a MOF creates coordination motifs that are inaccessible otherwise. The reduced species could be partially re-oxidized by molecular oxygen, even if both redox events were not quantitative.

The open coordination site postulated for the MOF did not translate into an increased catalytic activity towards cyclohexane oxidation. The testing showed the challenges to apply a testing procedure optimized for molecular catalysts to a heterogeneous catalyst, as leach tests suggested that catalysis is mainly due to leaching Cu species rather than true MOF reactivity. Some observations like color change and gas formation, however, remain suggestive of a Fenton-like behavior of the heterogeneous systems similar to that of molecular systems.

## Conflicts of interest

There are no conflicts to declare.

## Supplementary Material

DT-053-D3DT03096B-s001

DT-053-D3DT03096B-s002
