# Supplementary material for: Enabling a bioinspired N,N,N-copper coordination motif through spatial control in UiO-67: synthesis and reactivity
Source: Dalton Trans. 2024 Mar 12;53(19):8141–53. doi: 10.1039/d3dt03096b (PMC11091859; doi:10.1039/d3dt03096b)
Supplement: DT-053-D3DT03096B-s001 [file DT-053-D3DT03096B-s001.pdf]

Electronic Supplementary Material (ESI) for Dalton Transactions.
This journal is © The Royal Society of Chemistry 2024

Supporting Information

Enabling a Bioinspired *N,N,N*- Copper Coordination through Spatial Control in UiO-67: Synthesis and Reactivity

Isabelle Gerz,[a,b] Erlend S. Aunan, [a,b] Valeria Finelli, [c,d], Mouhammad Abu Rasheed, [a,b] Gabriele Deplano,[c] Rafael Cortez S. P.,[a,b] Inga L. Schmidtke,[a,b,e] David S. Wragg, [a,b] Matteo Signorile,[c] Knut T. Hylland, [a,b] Elisa Borfecchia,[c] Karl Petter Lillerud, [a,b] Silvia Bordiga,[c*] Unni Olsbye, [a,b*] Mohamed Amedjkouh [a,b*]

[a] *Department of Chemistry, University of Oslo, P. O. Box 1033 Blindern, N-0315 Oslo, Norway*

[b] *Centre for Materials Science and Nanotechnology, University of Oslo, P.O. Box 1126 Blindern, N-0316 Oslo, Norway*

[c] *Department of Chemistry, NIS and INSTM Reference Centre, Università di Torino, Via G. Quarello 15/A, I-10135, and Via P. Giuria 7, I-10125, Turin, Italy*

[d] *University School for Advanced Studies, IUSS Pavia, Palazzo del Broletto, Piazza della Vittoria 15, I-27100, Pavia, Italy*

[e] *Hylleraas Centre for Quantum Molecular Sciences, Department of Chemistry, University of Oslo, N-0315 Oslo, Norway*

Contents

Contributions 3

General 5

1. Synthesis and Characterization 8

1.1 Synthesis of the Linker 8

Synthesis of Methyl 6-(4-(methoxycarbonyl)-2-aminophenyl)nicotinate (**2**) 8

Synthesis of the Synthetic Intermediate **3** 11

Synthesis of Me_2**1** 12

Synthesis of H_2**1**·HCl 17

1.2 Syntheses of the Molecular Copper Complexes 21

Synthesis of [(Me_2**1**)$_2$Cu][OTf] 21

Synthesis of [(Me_2**1**)$_2$Cu][BF_4] 24

Synthesis of [Me_2**1**Cu][BF_4]$_2$ 29

Synthesis of Me_2**1**$CuCl_2$ 30

1.3 Syntheses of Metal Organic Frameworks 31

Synthesis of UiO-67-**1** 31

Synthesis of UiO-67-[**1**Cu][BF_4]$_2$-**LL** 31

Synthesis of UiO-67-[**1**Cu][BF_4]$_2$-**ML** 31

Synthesis of UiO-67-[**1**Cu][BF_4]$_2$-**HL** 32

Removing Residual H_2BPDC 32

Powder X-ray Diffraction 33

Variable Temperature pXRD of UiO-67-[1Cu][BF_4]$_2$-**HL** 35

Digestion ^{1}H NMR of Metal-Organic Frameworks 35

Thermogravimetric Analysis 36

Energy Dispersive X-ray Spectroscopy (EDS) 39

Estimation of MOF Composition 39

Elemental Analysis 40

Microwave Plasma Atomic Emission Spectroscopy (MP-AES) 40
N_2 Adsorption 41
2. X-Ray Absorption Spectroscopy 45
2.1 EXAFS Fitting for as-prepared UiO-67-1[Cu(BF_4)$_2$]-ML and -HL.............. 45
EXAFS Fitting Model 45
EXAFS Fit Results.. 45
3. UV/Vis Spectroscopy 49
3.1 MOF Reactivity.. 49
4. Catalytic testing 52
4.1 Test and Work-up Procedures 52
4.2 Selectivity Studies 52
4.3 Leaching Test of MOF Sample... 53
4.4 Analysis equipment 53
4.5 NMR Analysis of the Digested MOF UiO-67-[1Cu][BF_4]$_2$ 53
5. Bibliography 56

Contributions

Isabelle Gerz

Investigation: Synthesis Plan Molecular Compounds: Equal; Execution of the Synthesis and Characterisation of all Molecular Complexes and Organic Compounds other than those synthesized by KTH and RCSP, UV/Vis studies: Support; XAS measurements: Support, Writing Original Draft: Lead, Writing – review & editing: Lead

Erlend S. Aunan

Investigation: Synthesis and Characterisation of all MOFs; XAS measurements: Support, Writing Original Draft: Support, Writing – review & editing: Support

Valeria Finelli

Investigation: Spectroscopic Characterization of the MOFs (IR and UV/Vis studies: Lead); Writing Original Draft: Support, Writing – review & editing: Support

Mouhammad Abu Rasheed

Investigation: Conducting catalytic testing/leaching studies in liquid phase; NMR studies on digested MOFs: Support, Writing Original Draft: Support, Writing – review & editing: Support

Gabriele Deplano

Investigation: UV/Vis studies: support; XAS measurements: support; Writing - review & editing: support.

Rafael Cortez S. P.

Synthesis and Characterization of $[(Me_2\mathbf{1})_2Cu][BF_4]$, Writing – review & editing: Support

Inga L. Schmidtke

XRD analysis, Writing – review & editing: Support

David S. Wragg

XRD analysis, Writing – review & editing: Support

Matteo Signorile

Supervision G.D., XAS measurements: Lead, Writing - review & editing: Support.

Knut T. Hylland

Investigation: Synthesis Plan Molecular Compounds: Equal; Synthesis and Characterisation of methyl 6-(4-(methoxycarbonyl)-2-aminophenyl)nicotinate, $H_2\mathbf{1}$, ^{15}N NMR analysis, Writing Original Draft: Support, Writing – review & editing: Support

Continued on next page

Elisa Borfecchia

Investigation: XAS analysis: Lead, Writing Original Draft: Support, Writing – review & editing: Support

Karl Petter Lillerud

Supervision ESA

Unni Olsbye

Supervision MAR, Writing– review & editing: Support

Silvia Bordiga

Supervision VF, Writing – review & editing: Support

Mohamed Amedjkouh

Supervision IG, Writing – review & editing: Support

General

All syntheses involving Cu(I) species were carried out in an Ar-filled UNIlab pro glovebox from MBraun. All solvents used with copper(I) species were degassed with the freeze-pump-thaw technique (3 cycles). UiO-67 was obtained from ProfMOF A/S, with an estimated chemical formula of $Zr_6O_4(OH)_4(BPDC)_{5.3}(benzoate)_{0.4}(OH/H_2O)_{1.0}$. The chemicals for synthesis were used as received from commercial suppliers, unless noted otherwise. Chemicals for catalytic testing: Aluminium Oxide 90 standardized for column chromatography adsorption analysis from Millipore, Germany. Aluminium oxide activated, acidic, Brockmann-(|) from Sigma-Aldrich, Switzerland. Acetonitrile 100% for HPLC, Diethyl ether 100%, and Hydrogen peroxide 33% water solution from VWR Chemicals, UK. Cyclohexane 99,5% anhydrous, Cyclohexanol ReagentPlus® 99%, Cyclohexanone 99,5%, and Cycloheptanone 99% from Sigma-Aldrich, Germany. Triphenylphosphine 99% from Fluka Chemica, Switzerland. Tetrakis(acetonitrile)copper(I) tetrafluoroborate 97%, and Copper(II) tetrafluoroborate hydrate from Sigma-Aldrich, Switzerland.

Melting points, if measured, are uncorrected and were measured on a Stuart SMP10 instrument.

NMR spectra were recorded on the following Bruker instruments: DPX300, AVIII400, DRX500, AVI600, AVII600, AVIIIHD800. ^{1}H and ^{13}C NMR spectra are referenced to residual solvent signals. ^{15}N NMR signals are referenced to an external nitromethane standard. All NMR spectra were recorded at ambient temperature, unless specified. The NMR samples of compounds containing copper were prepared in the glovebox in commercial 9 inch NMR tubes. The samples were transferred out of the glovebox with an adapter. The sample was connected to a vacuum line and the solvent was frozen with liquid nitrogen. The tube was flame sealed under vacuum.

Powder X-ray diffraction was performed with a Bruker D8 Discover diffractometer, using Cu $K_{\alpha 1}$ radiation selected by a Ge (111) Johanssen monochromator, with exception of sample UiO-67-**1** which was measured in capillary tubes using synchrotron radiation (λ = 0.3393 Å). For the sake of ease of comparison, all diffractograms are recalculated to $Cu_{K\alpha}$ wavelength (λ = 1.5406 Å). MOF-samples were digested in 1.0 M NaOD in D_2O prior to ^{1}H NMR analysis. DSS was used as internal standard. MS (ESI) was recorded on a Bruker maXis II ETD spectrometer. Elemental analysis was performed by Mikroanalytisches Laboratorium Kolbe, Oberhausen, Germany.

Thermogravimetric analyses were conducted on a NETZSCH STA 449 F3 Jupiter, ramping from 30 to 900 °C with a 10 °C/min ramping rate. The samples were under a stream of synthetic air, consisting of a 20 mL/min flowrate of N_2 and 5 mL/min flowrate of O_2. SEM images were taken on a Hitachi SU8230 Field Emission Scanning Electron Microscope (FE-SEM). Single crystal diffraction data were acquired on a Bruker D8 Venture equipped with a Photon 100 detector and using Mo Kα radiation (λ = 0.71073 Å) from an Incoatec iµS microsource. The data reduction was performed with the Bruker Apex3 Suite, the structures were solved with ShelXT and refined with ShelXL.[1,2]

Olex2 was used as user interface.[3] The cif files were edited with enCIFer v. 1.4.[4] and molecular graphics were produced with Diamond v. 4.6.2.

UV/Vis-NIR measurements were performed on a Specord 200 Plus instrument for solutions. Diffuse reflectance (DR) UV/Vis-NIR *ex situ* spectra were recorded on a Varian Cary5000 spectrophotometer equipped with a reflectance sphere. The MOFs in powder form were directly placed inside the sample holder. Spectralon powder was used as a standard for 100% reflectance. Transmission (T) UV/Vis spectrum of ligand **1** as 10^{-4} M solution in MeCN was collected on a Varian Cary300 spectrophotometer by placing the solution in a 10 mm optical path quartz cuvette. DR UV-Vis measurements were also performed under different gas flow by using an Avantes AvaSpecULS2048XL-EVO fibre optics spectrometer (100 µm slits), coupled to an Avantes AvaLight-DH-S light source (equipped with a deuterium and a halogen lamp) with an integration time of 50 ms and averaging 20 scans per spectrum. A custom-designed high-temperature resistant fibre optic with a high-OH fused silica core of 100 µm in diameter was used for the collection of diffuse reflected light from the sample. Polytetrafluoroethylene (PTFE) powder was used as a standard for 100% reflectance. All measurements were performed in a quartz tubular reactor (6 mm external diameter, 4 mm internal diameter), by loading approximately 30 mg of sample (250–500 µm sieved fraction). The reactor was inserted into a cylindrical oven powered by a power supply controlled through a PID. This apparatus can be coupled to gas feeding system equipped with digital mass flow controllers (Bronkhorst El-Flow) for the samples activation under 30 mL/min of He from ambient temperature till 150 °C (heating rate 5 °C/min). They were kept at this temperature for 60 min, then they were treated for another hour under 30 mL/min O_2 flow.

IR spectra were collected in transmission mode, using a home-made quartz cell, combined with a Bruker INVENIO R spectrophotometer equipped with a Mercury-Cadmium-Telluride (MCT) cryogenic detector. Prior to the transmission IR measurements, the samples were pelletized at a pressure of 0.5 ton: the use of higher pressures was avoided to prevent samples partial amorphization. Then, the self-supported pellets were placed in a quartz cell able to reach low temperatures and connected to a vacuum line for the materials activation. The samples were outgassed overnight under dynamic vacuum. Once reached the activation temperature (150 °C) with heating rate 5 °C/min, they were outgassed for 90 min. Then the IR spectra were collected with a resolution of 2 cm^{-1} by accumulating 32 scans. After IR measurements, powder X-Ray Diffraction (pXRD) patterns were registered on a PANalytical X' Pert instrument with Cu Kα radiation corresponding to an incident λ = 1.5405 Å. The patterns were acquired in the 2θ range of 3–30° with a step of 0.02° in Bragg-Brentano geometry, to check the pellets stability of the samples re-exposed to air at the end of the spectroscopic experiments.

In situ XAS data on UiO-67-[**1**Cu][BF_4]$_2$-**HL**

were collected at the BM31[5] beamline of the European Synchrotron Radiation Facility (ESRF, Grenoble, France). The experiments were conducted by loading ca. 10 mg of powder in a 1 mm diameter capillary reactor connected to an appropriate gas-flow setup. All the gas flows were regulated by mass flow controllers and set to 20 ml/min.

Temperature at the data collection point was controlled by a calibrated heat gun, and a heating rate of 5 °C/min was employed throughout the experiment. The UiO-67-[**1**Cu][BF_4]$_2$-HL sample was characterized (i) at RT in He gas flow, (ii) after heating and keeping the sample for 60 min at 150 °C always in He flow, and (iii) after subsequent exposure to pure O_2 flow for 60 min, under isothermal conditions at 150 °C.

Cu K-edge XAS spectra were collected in transmission mode, using a water-cooled flat Si(111) double crystal monochromator. The incident (I_0) and transmitted (I_1) X-ray intensities were detected using 30 cm-length ionization chambers filled with a mixture of He and Ar. The XAS spectrum of a Cu metal foil was simultaneously collected by means of a third ionization chamber (I_2), for energy calibration/alignment purposes. Continuous scans were performed in the 8800–9795 eV range, with a constant energy step of 0.5 eV; acquisition time was ca. 3 min/scan.

The XAS scans were normalized to unity edge jump and calibrated/aligned in energy using the Athena software from the Demeter package.[6] Higher-quality spectra were obtained by merging 5 scans collected upon stabilization at each treatment step, after checking for the signal reproducibility. For the three average spectra, the $\chi(k)$ EXAFS functions were extracted by using the Athena program. Fourier-transform (FT) EXAFS spectra were obtained by transforming the $k^2\chi(k)$ functions in the (2.5–12.0) $Å^{-1}$ range. EXAFS fitting for the as-prepared material was performed using the Artemis program from the same package.[6]

For comparison purposes, *ex situ* Cu K-edge XAS spectra for as-prepared UiO-67-[**1**Cu][BF_4]$_2$-**ML** and UiO-67-[**1**Cu][BF_4]$_2$-**LL** were collected on the BM23 beamline of the ESRF.[7] The samples were prepared in the form of self-supporting pellets with mass optimized for XAS data collection in transmission mode and measured at RT in air.

1. Synthesis and Characterization

1.1 Synthesis of the Linker

Synthesis of Methyl 6-(4-(methoxycarbonyl)-2-aminophenyl)nicotinate (**2**)

Methyl 6-(4-(methoxycarbonyl)-2-nitrophenyl)nicotinate was synthesized according to the literature procedure published by Hylland et al.[8] In that publication, the compound is denominated **1n**. Methyl 6-(4-(methoxycarbonyl)-2-nitrophenyl)nicotinate (1.49 g, 4.71 mmol, 1.0 equiv.) was suspended in AcOH (50 mL), and Fe powder (3.94 g, 70.6 mmol, 15 equiv.) was added in one portion. The reaction mixture was flushed with Ar for 2 h, and then stirred at RT under Ar atmosphere for 18 h. The reaction mixture was poured into a beaker containing ice (ca. 100 mL), and concentrated ammonia solution (28 % aq., ca. 100 mL) was added in portions of 25 mL. More ice was added to the neutralization if the mixture warmed up significantly. After all ammonia had been added, the resulting suspension was stirred for 5 min, the solids were collected through filtration and washed with water (3x 100 mL). The solids were then dissolved in warm EtOAc (ca. 50−60 °C, 300 mL) under stirring. The warm EtOAc solution was filtered through Celite to remove undissolved material. The Celite was rinsed with additional EtOAc (total 100 mL). The intensely yellow filtrate and the washings were combined and dried over Na_2SO_4. Removal of the solvent under reduced pressure, followed by recrystallization of the crude product from MeCN gave **2** as orange crystals. Yield: 1.24 g, 4.33 mmol, 92 %.

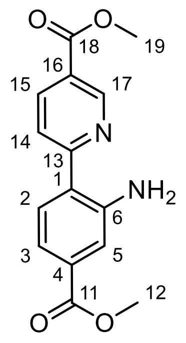

M.p. 188-189 °C; ^{1}H NMR (600 MHz, d_6-DMSO): δ[ppm] 9.14 (1H, d, $^4J_{H,H}$ = 2.3 Hz, H17), 8.33 (1H, dd, $^3J_{H,H}$ = 8.5 Hz, $^4J_{H,H}$ = 2.3 Hz, H15), 8.03 (1H, d, $^3J_{H,H}$ = 8.5 Hz, H14), 7.77 (1H, d, $^3J_{H,H}$ = 8.3 Hz, H2), 7.47 (1H, d, $^4J_{H,H}$ = 1.8 Hz, H5), 7.17 (1H, dd, $^3J_{H,H}$ = 8.2 Hz, $^4J_{H,H}$ = 1.8 Hz, H3), 7.03 (2H, broadened s, NH_2), 3.91 (3H, s, H19), 3.84 (3H, s, H12). ^{13}C NMR (150 MHz, d_6-DMSO): δ[ppm] 166.2 (C11), 165.0 (C18), 161.4 (C13), 148.8 (C17), 148.4 (C6), 137.6 (C15), 131.2 (C4), 129.8 (C2), 122.8 (C16), 122.0 (C1), 121.9 (C14), 117.7 (C5), 115.7 (C3), 52.4 (C19), 52.1 (C12). ^{15}N{^{1}H} NMR (800 MHz, d_6-DMSO): δ[ppm] –77.6 (N_{Pyr}), –313.2 (N_{amine}). ESI-MS: m/z 309.085 (100%, $[M+Na]^+$). HRMS m/z $[M+Na]^+$ ($C_{15}H_{14}N_2NaO_4^+$): Calcd: 309.0846 Found: 309.0846. Anal. Calcd: C, 62.93; H, 4.93; N, 9.79 Found: C, 62.82; H, 4.89; N, 9.76.

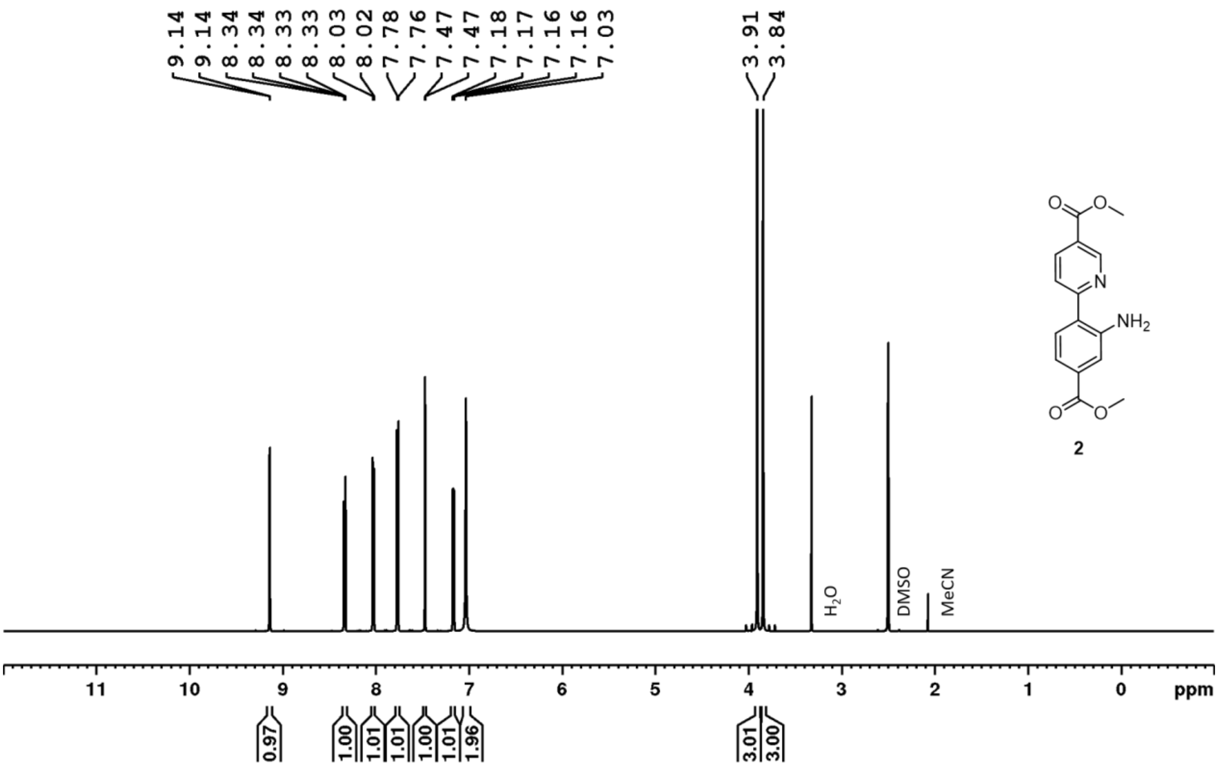

Figure S1. ^{1}H NMR spectrum (600 MHz, d_6-DMSO) of **2**.

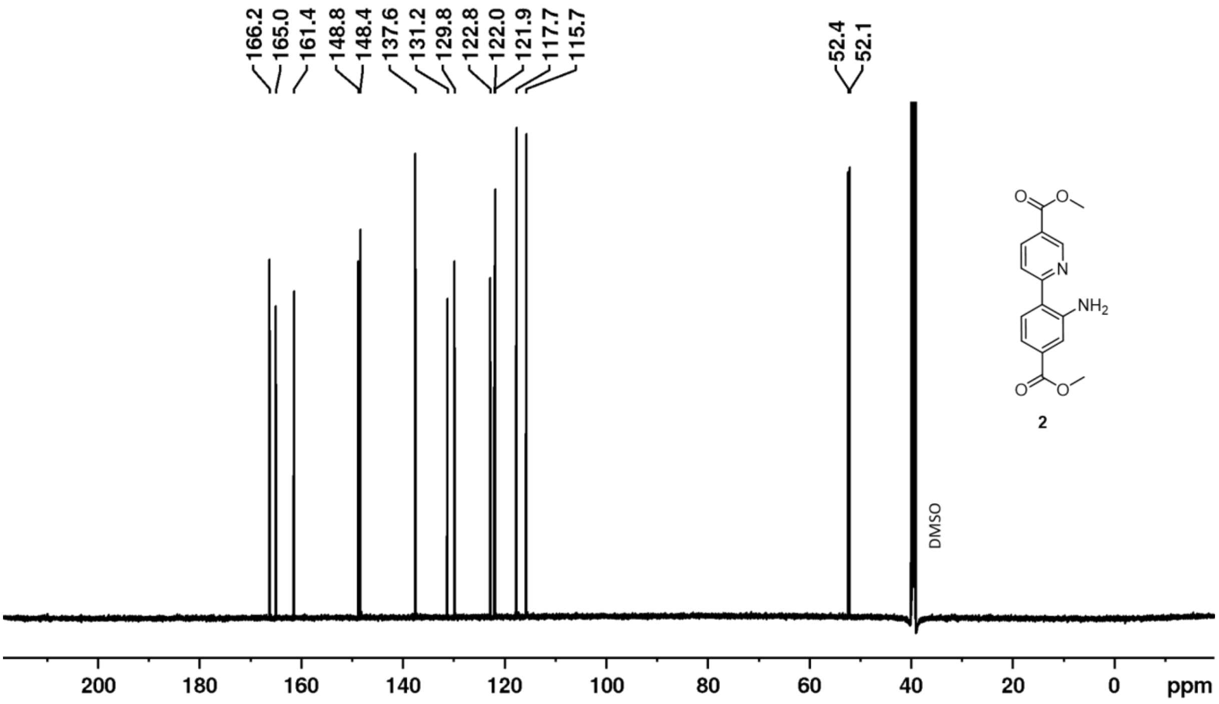

Figure S2. ^{13}C NMR spectrum (150 MHz, d_6-DMSO) of **2**.

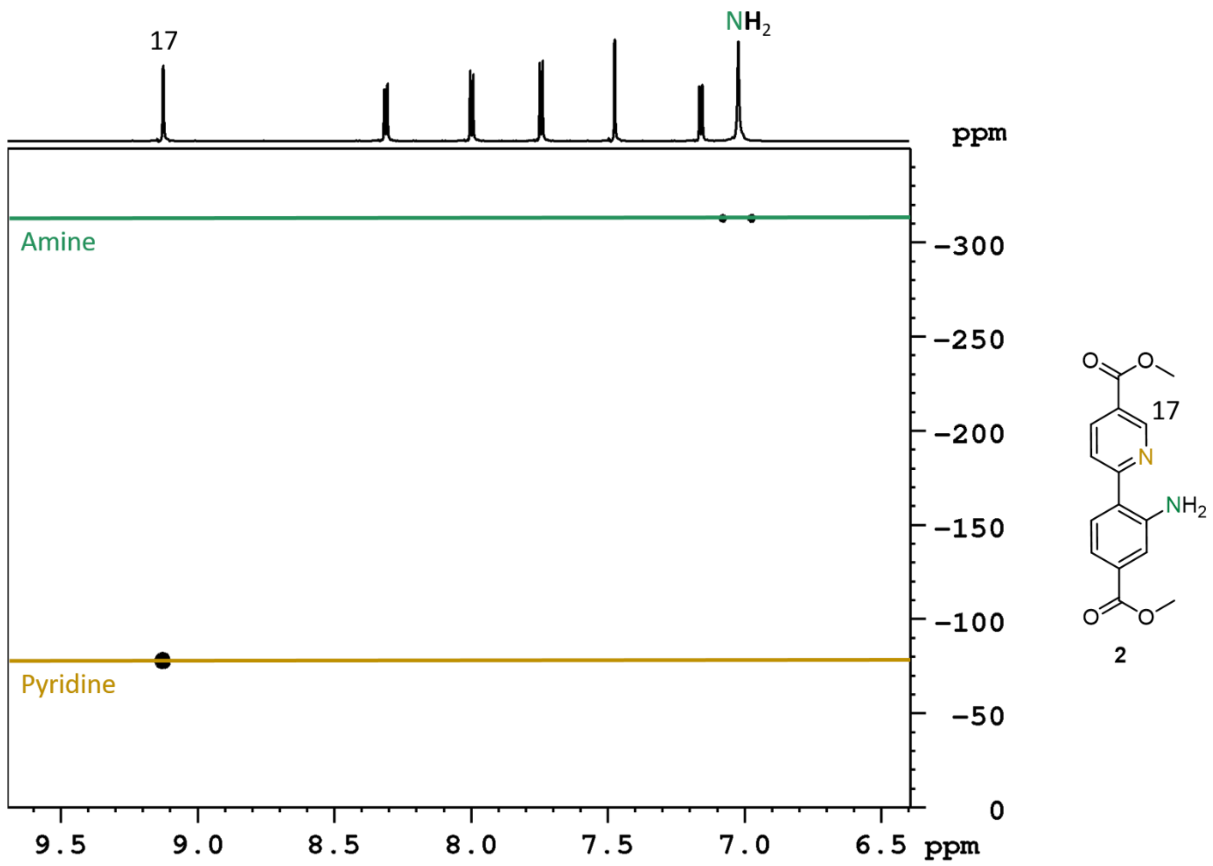

Figure S3. ^{1}H-^{15}N HMBC (800 MHz, d_6-DMSO) of **2**. N_{amine} is detected through its HSQC double peak.

Synthesis of the Synthetic Intermediate **3**

2 (4.00 g, 14.0 mmol), 4imidazolecarboxaldehyde (1.34 g, 14.0 mmol, 1.0 equiv.) and $Zn(OTf)_2$ (5.08 g, 14 mmol, 1.0 equiv.) were stirred for three days in a mixture of ethyl acetate (20 mL) and acetonitrile (10 mL). Solids were collected through filtration and washed with ethyl acetate (30 mL). After drying, **3** was obtained as a colorless solid (Yield: 7.13 g, 9.80 mmol, 70 %).

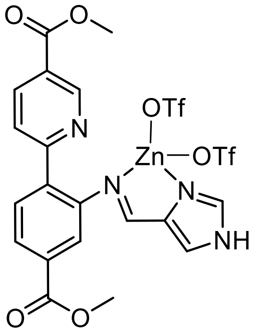

^{1}H NMR (300 MHz, d_6-DMSO): δ[ppm] 13.46 (1H, s), 9.01 (1H, s), 8.58 (1H, s), 8.28 (1H, d, , $^{3}J_{H,H}$ = 7 Hz), 8.14 (1H, s), 8.1-7.8 (4H, m, overlap), 7.52 (1H,s), 3.91 (3H, s), 3.89 (3H, s). ESI-MS: *m/z* 365.124 (72 %, [L+H^{+}]), 387.106 (100 %, [L+Na^{+}]). Anal. Calcd: C, 34.65; H, 2.22; N, 7.70; Zn, 8.98 Found: C, 34.53; H, 2.23; N, 8.08; Zn, 8.31.

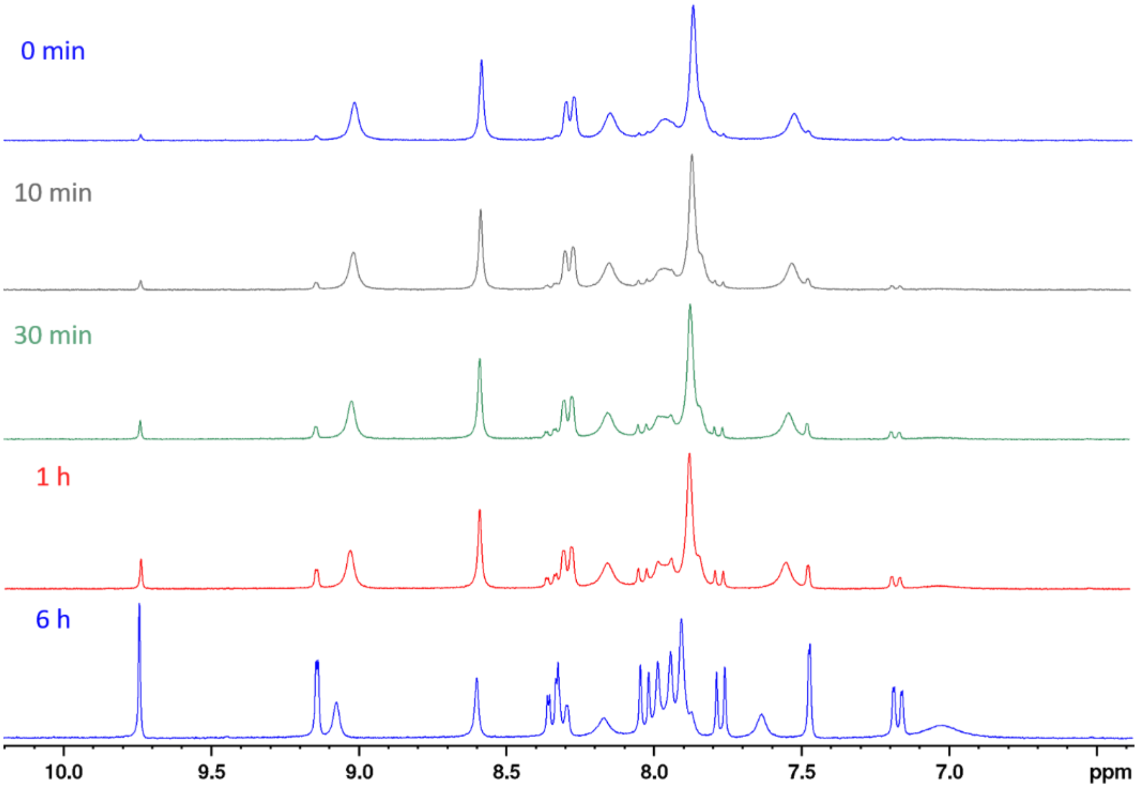

Figure S4. Aromatic region of the ^{1}H NMR (300 MHz, d_6-DMSO) spectra of **3** over time. Decomposition through hydrolysis is observed (see evolution of an aldehyde peak at 9.7 ppm).

Synthesis of Me_2**1**

3 (7.13 g, 9.79 mmol) was suspended in dry MeOH (100 mL). The suspension was cooled in an ice bath and $NaBH_4$ (1.85 g, 48.9 mmol, 5 equiv.) was added portionwise over the course of 10 min. The ice bath was removed and the mixture was stirred at ambient temperature for 2 h. The reaction mixture was poured into sat. $NaHCO_3$ solution (aq., 100 mL). The aqueous phase was extracted twice with CH_2Cl_2 (300 mL and 100 mL). The combined organic phases were washed with Na_2EDTA solution (aq., 200 mL) and dried over Na_2SO_4. After removal of the solvent, the product was purified by column chromatography (CH_2Cl_2 → CH_2Cl_2:MeOH 95:5). The product was obtained as a bright yellow solid (Yield: 2.91 g, 7.93 mmol, 81 %).

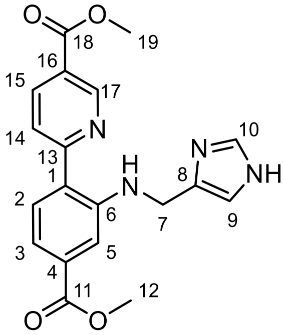

^{1}H NMR (800 MHz, d_6-DMSO): δ[ppm] 11.90 (1H, s, imidazole N**H**), 9.07 (1H, d, $^{4}J_{H,H}$ = 2.1 Hz, H17), 8.96 (1H, t, $^{3}J_{H,H}$ = 5.0 Hz, N**H**), 8.37 (1H, dd, $^{3}J_{H,H}$ = 8.5 Hz, $^{4}J_{H,H}$ = 2.3 Hz, H15), 8.09 (1H, d, $^{3}J_{H,H}$ = 8.5 Hz, H14), 7.85 (1H, d, $^{3}J_{H,H}$ = 8.2 Hz, H2), 7.63 (1H, s, H10), 7.40 (1H, d, $^{4}J_{H,H}$ = 1.6 Hz, H5), 7.26 (1H, dd, $^{3}J_{H,H}$ = 8.2 Hz, $^{4}J_{H,H}$ = 1.6 Hz, H3), 7.02 (1H, s, H9), 4.34 (2H, d, $^{3}J_{H,H}$ = 4.9 Hz, H7), 3.90 (3H, s, H19), 3.85 (3H, s, H12). ^{13}C NMR (200 MHz, d_6-DMSO): δ[ppm] 166.3 (C11), 164.9 (C18), 161.3 (C13), 148.3 (C17), 147.7 (C6), 137.9 (C8), 137.7 (C15), 135.2 (C10), 131.7 (C1), 130.0 (C2), 123.0 (C4), 122.7 (C16), 122.3 (C14), 115.6 (C3), 112.8 (C9), 112.1 (C5), 52.4 (C19), 52.2 (C12), 41.0 (C7). ^{15}N{^{1}H} NMR (800 MHz, d_6-DMSO): δ[ppm] –80.1 (N_{Pyr}), –119.6 (N_{IM}), –215.1 (N_{AZ}), –307.7 (N_{amine}). The ^{15}N shifts were collected in two separate experiments (Figure S7 and Figure S8). ESI-MS: m/z 367.140 (100%, $[M+H]^+$); HRMS m/z $[M+H]^+$ ($C_{19}H_{19}N_4O_4^+$): Calcd: 367.1401 Found: 367.1401. Anal. Calcd: C, 62.29; H, 4.95; N, 15.29 Found: C, 62.21; H, 4.93; N, 15.27.

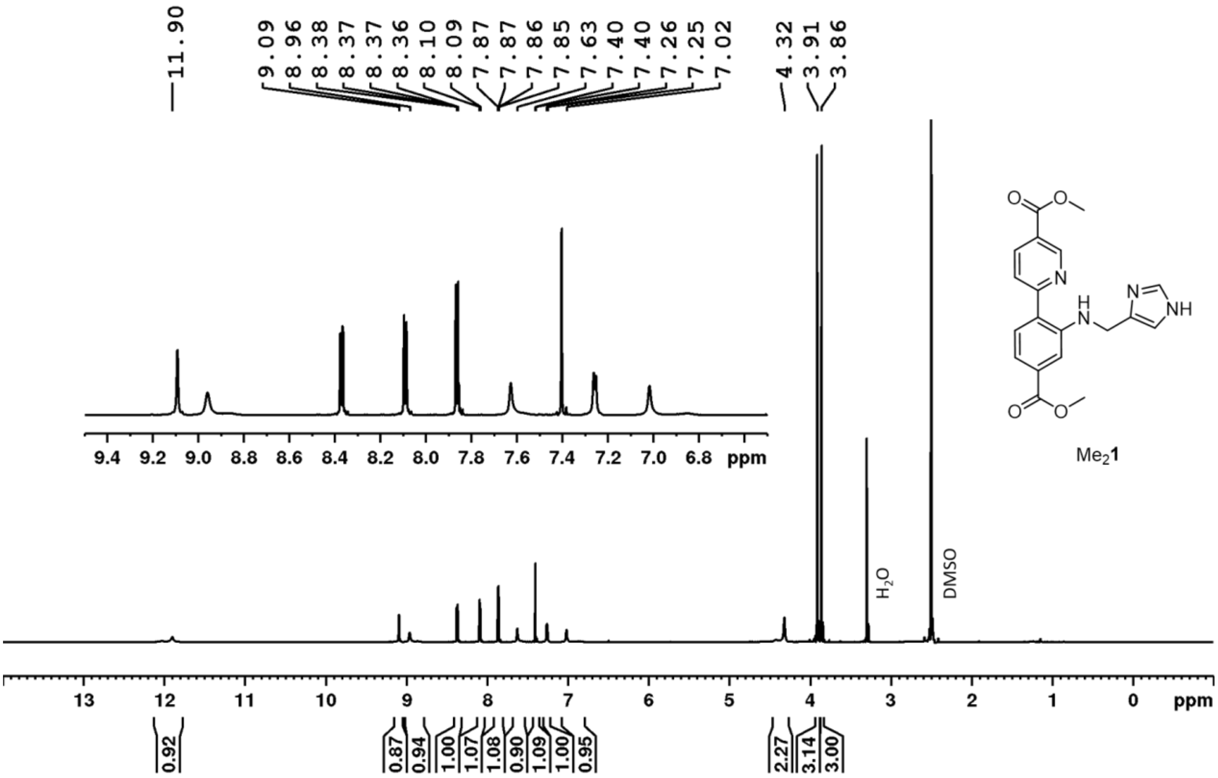

Figure S5. ^{1}H NMR spectrum (800 MHz, d_6-DMSO) of Me$_2$**1**.

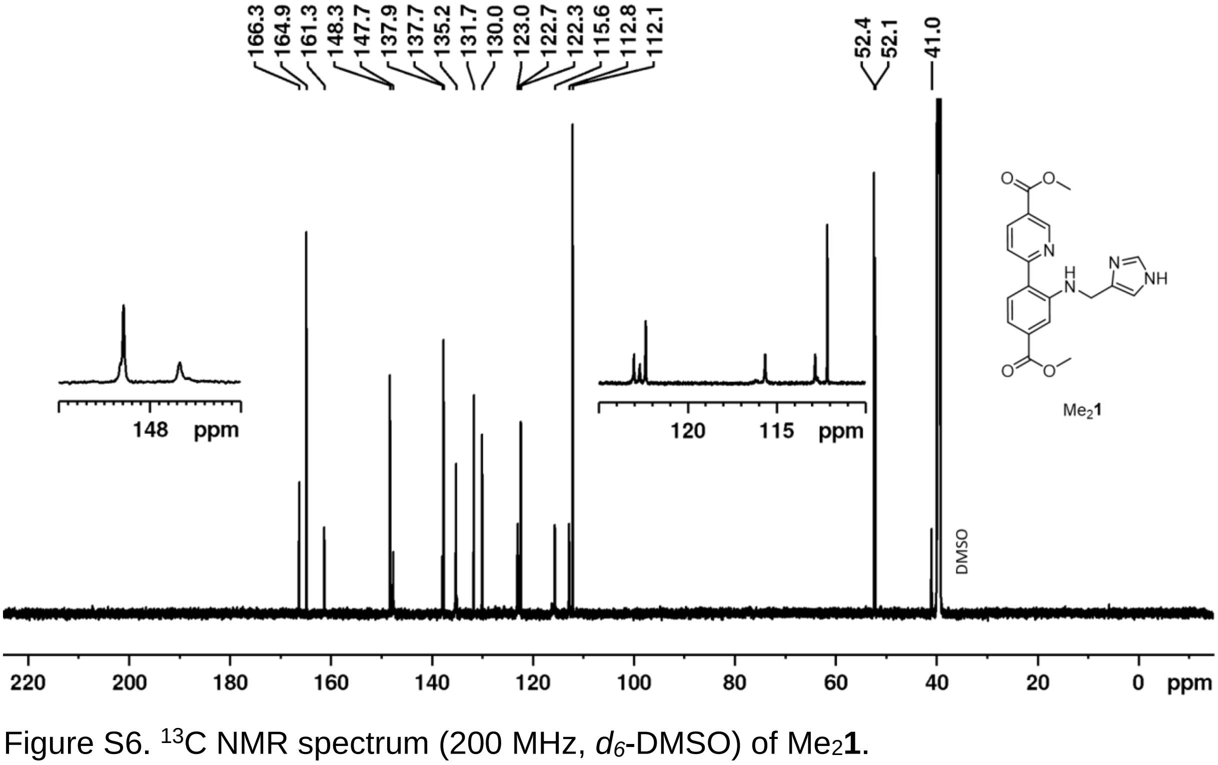

Figure S6. ^{13}C NMR spectrum (200 MHz, d_6-DMSO) of Me$_2$**1**.

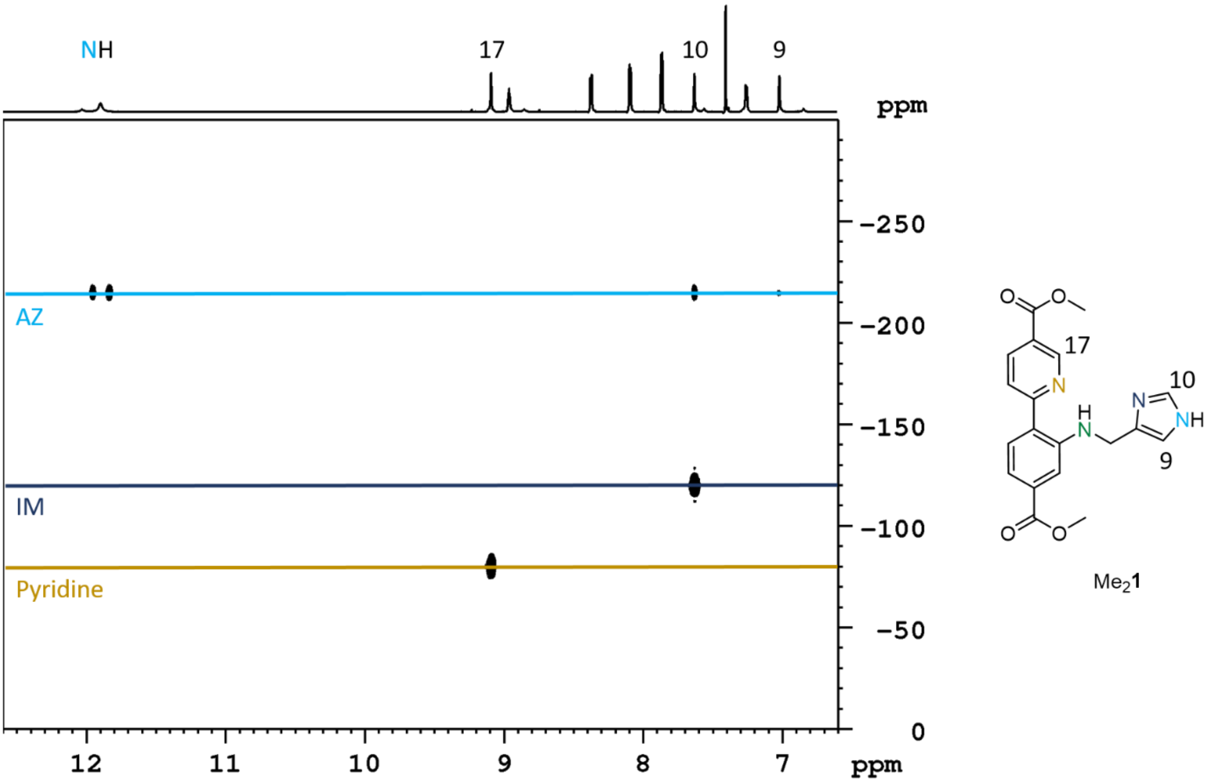

Figure S7. ^1H-^{15}N HMBC (800 MHz, d_6-DMSO) of Me$_2$**1**.

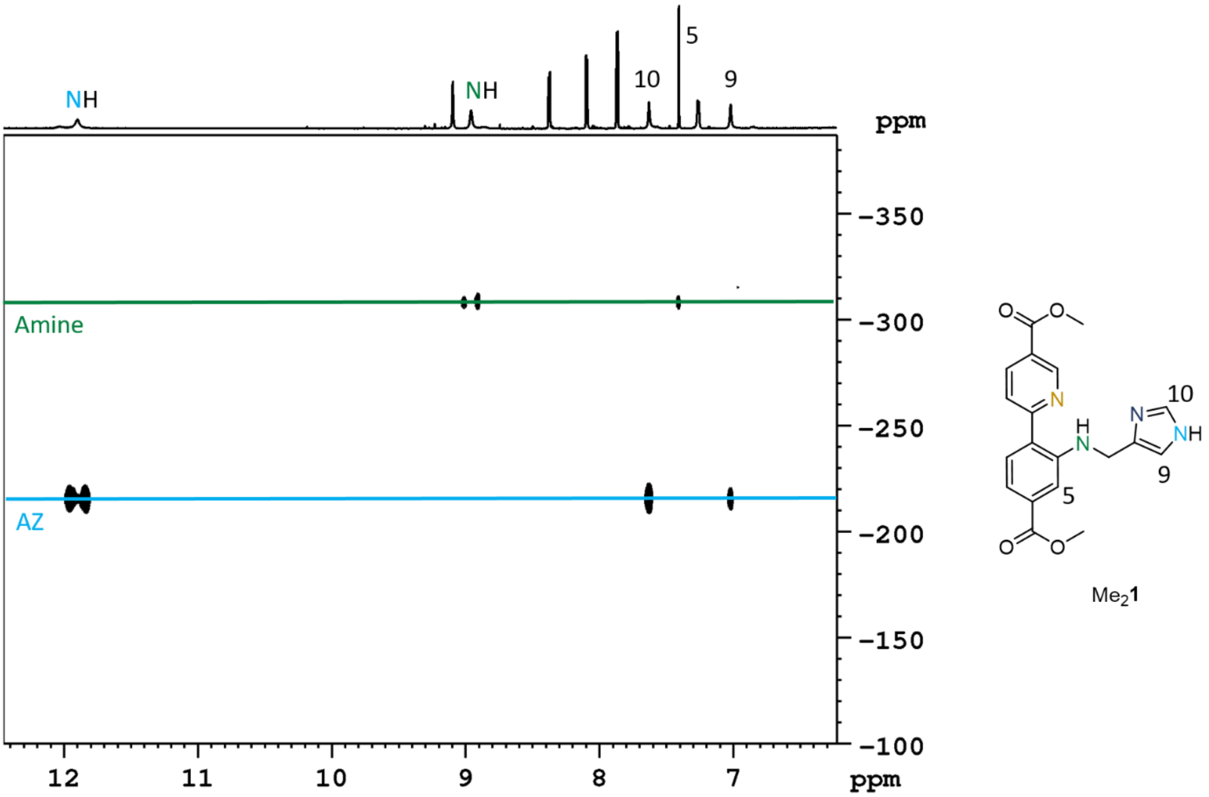

Figure S8. ^1H-^{15}N HMBC (800 MHz, d_6-DMSO) of Me$_2$**1** with upfield sweep width.

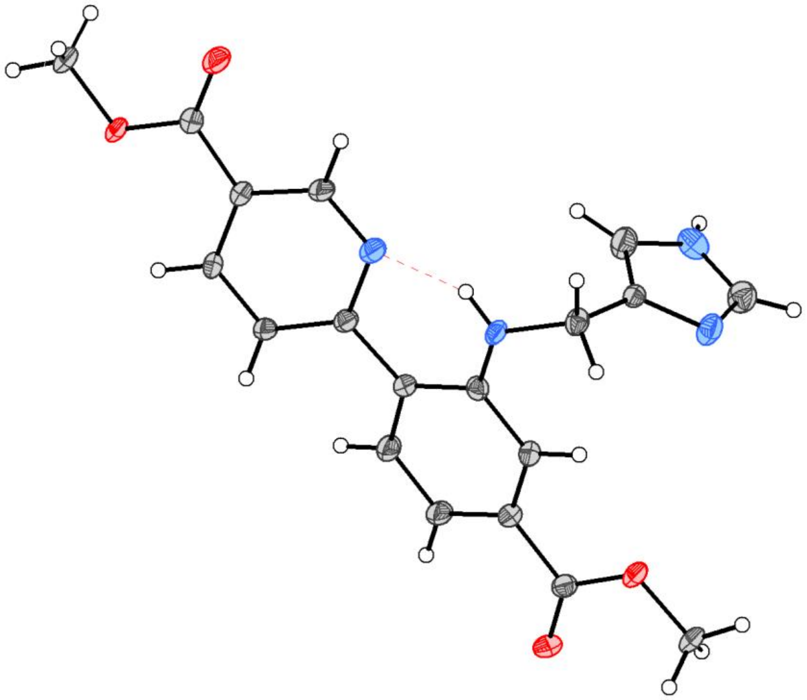

Figure S9. Single crystal XRD structure of Me_2**1** with ellipsoids at 50% probability.

Table S1. Crystal and refinement data for Me_2**1** (CCDC 2085805).

Crystal data	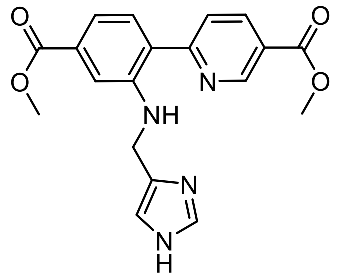
Chemical formula	$C_{19}H_{18}N_4O_4$
M_r	366.37
Crystal system, space group	Monoclinic, *Cc*
Temperature (K)	100
a, *b*, *c* (Å)	30.2632 (14), 17.8811 (8), 14.5204 (7)
β (°)	116.644 (1)
V (Å^3)	7023.2 (6)
Z	16
Radiation type	Mo *K*α
μ (mm^{-1})	0.10
Crystal size (mm)	0.47 × 0.42 × 0.32
Diffractometer	Bruker D8 Venture
Absorption correction	Multi-scan<br>*SADABS2016*/2 (Bruker,2016/2) was used for absorption correction. wR2(int) was 0.0705 before and 0.0506 after correction. The Ratio of minimum to maximum transmission is 0.9453. The λ/2 correction factor is Not present.
T_{min}, T_{max}	0.705, 0.746
No. of measured, independent and observed [$I > 2\sigma(I)$] reflections	67828, 17263, 14379
R_{int}	0.039
$(\sin \theta/\lambda)_{max}$ (Å^{-1})	0.669
Refinement	
$R[F^2 > 2\sigma(F^2)]$, $wR(F^2)$, S	0.045, 0.104, 1.01
No. of reflections	17263
No. of parameters	1009
No. of restraints	2
H-atom treatment	H atoms treated by a mixture of independent and constrained refinement
$\Delta\rho_{max}$, $\Delta\rho_{min}$ (e Å^{-3})	0.34, -0.28
Absolute structure	Flack x determined using 5902 quotients [(I+)-(I-)]/[(I+)+(I-)] (Parsons, Flack and Wagner, Acta Cryst. B69 (2013) 249-259).
Absolute structure parameter	0.1 (3)

Synthesis of H_2**1**·HCl

Me_2**1** (1.05 g, 2.92 mmol) and LiOH (0.280 g, 11.7 mmol, 4 equiv.) were stirred in a solvent mixture of H_2O (8 mL), THF (8 mL) and MeOH (4 mL) for a day at ambient temperature. The solvent volume was reduced to half of its original volume under reduced pressure. The protonated product was precipitated by addition of HCl (aq., conc.) until a pH~2 was reached. After addition of water (2-3 mL), the obtained product was collected by filtration and washed with acetone and THF. After drying, the compound was resuspended in THF (10 mL) and collected through filtration, then washed with acetone. This resuspension was performed twice. Drying in the vacuum oven at 70 °C gave H_2**1**·HCl (Yield: 0.889 g, 2.37 mmol, 81 %) as an orange powder.

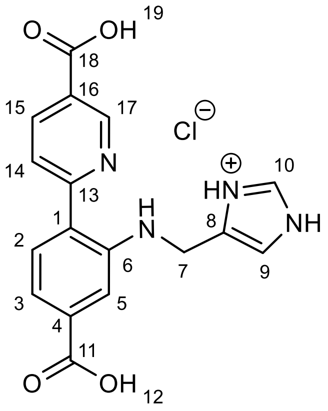

H_2**1**•HCl

^{1}H NMR (800 MHz, d_6-DMSO): δ[ppm] 14.50 (1H, s, COO**H**), 14.24 (1H, s, COO**H**), 13.45 (1H, *br*, s, imidazole N**H**), δ 12.97 (1H, *br*, s, imidazole N**H**), 9.14 (1H, d, $^{4}J_{H,H}$ = 2.2 Hz, H17), 9.02 (d, 8.96, $^{4}J_{H,H}$ = 0.8 Hz, H10), 8.37 (1H, dd, $^{3}J_{H,H}$ = 8.4 Hz, $^{4}J_{H,H}$ = 2.2 Hz, H15), 8.08 (1H, d, $^{3}J_{H,H}$ = 8.6 Hz, H14), 7.85 (1H, d, $^{3}J_{H,H}$ = 8.6 Hz, H2), 7.57 (1H, s, H9), 7.31 (2H, m, overlap of H3 and H5), 4.60 (2H, s, H7), amine N**H** missing. ^{13}C NMR (200 MHz, d_6-DMSO): δ[ppm] 167.2 (C11), 165.9 (C18), 160.8 (C13), 148.7 (C17), 146.6 (C6), 138.0 (C15), 134.4 (C10), 132.8(C1), 131.4 (C8), 130.1 (C2), 124.3 (C16), 123.8 (C4),122.5 (C14), 117.2 (C3), 116.5 (C9), 112.1 (C5), 37.1 (C7). $^{15}N\{^{1}H\}$ NMR (800 MHz, d_6-DMSO): δ[ppm] –83.1 (N_{Pyr}), –207.4 (N_{AZ}), N_{IM} and N_{amine} missing. ESI-MS: m/z 361.091 (100%, $[M+Na]^+$); HRMS m/z $[M+Na]^+$ ($C_{17}H_{14}N_4NaO_4^+$): Calcd: 361.0907 Found: 361.0907. Anal. Calcd: C, 54.48; H, 4.03; N, 14.95; Cl, 9.46 Found: C, 54.21; H, 3.97; N, 14.94; Cl, 9.48.

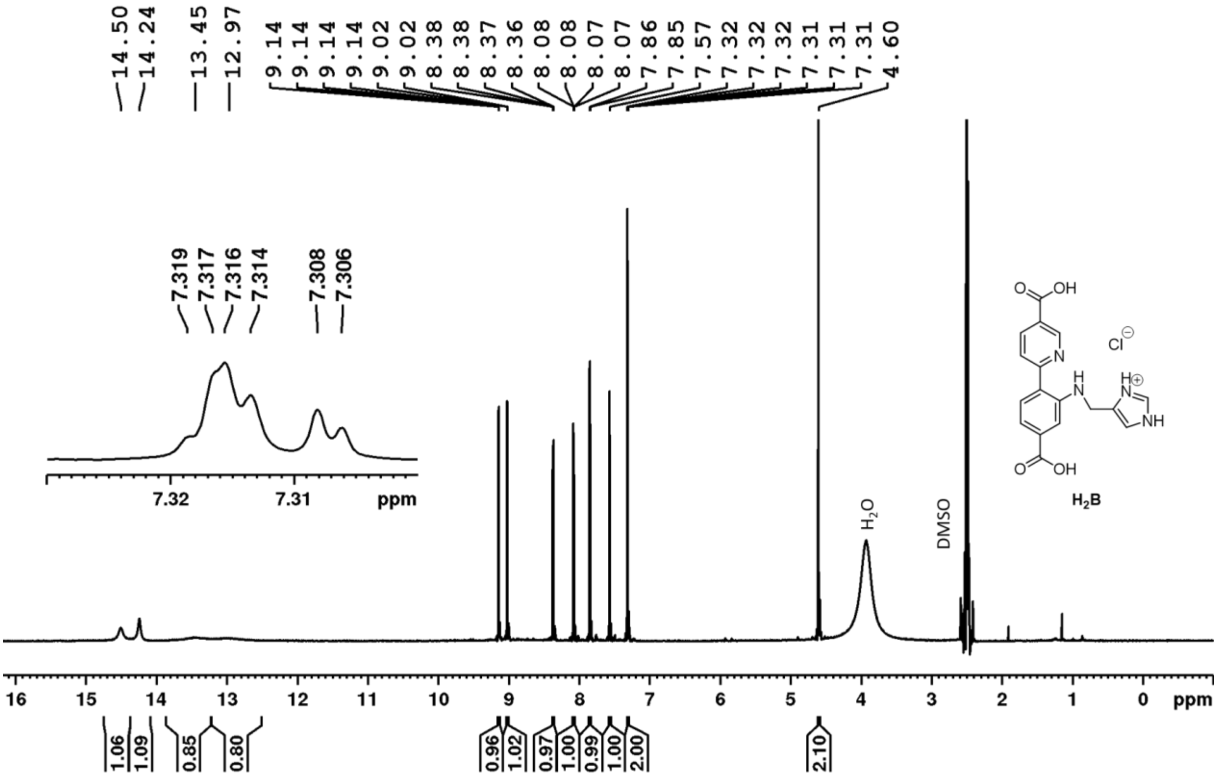

Figure S10. ^{1}H NMR spectrum (800 MHz, d_6-DMSO) of H_2**1**.

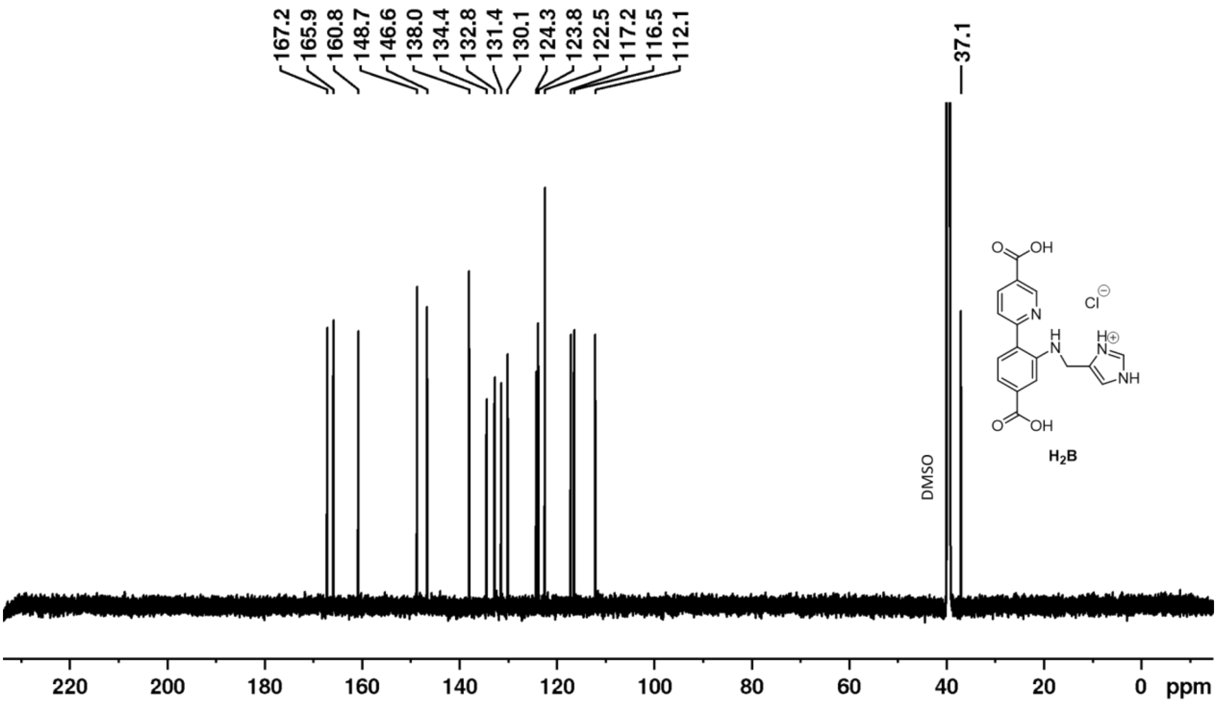

Figure S11. ^{13}C NMR spectrum (200 MHz, d_6-DMSO) of H_2**1**.

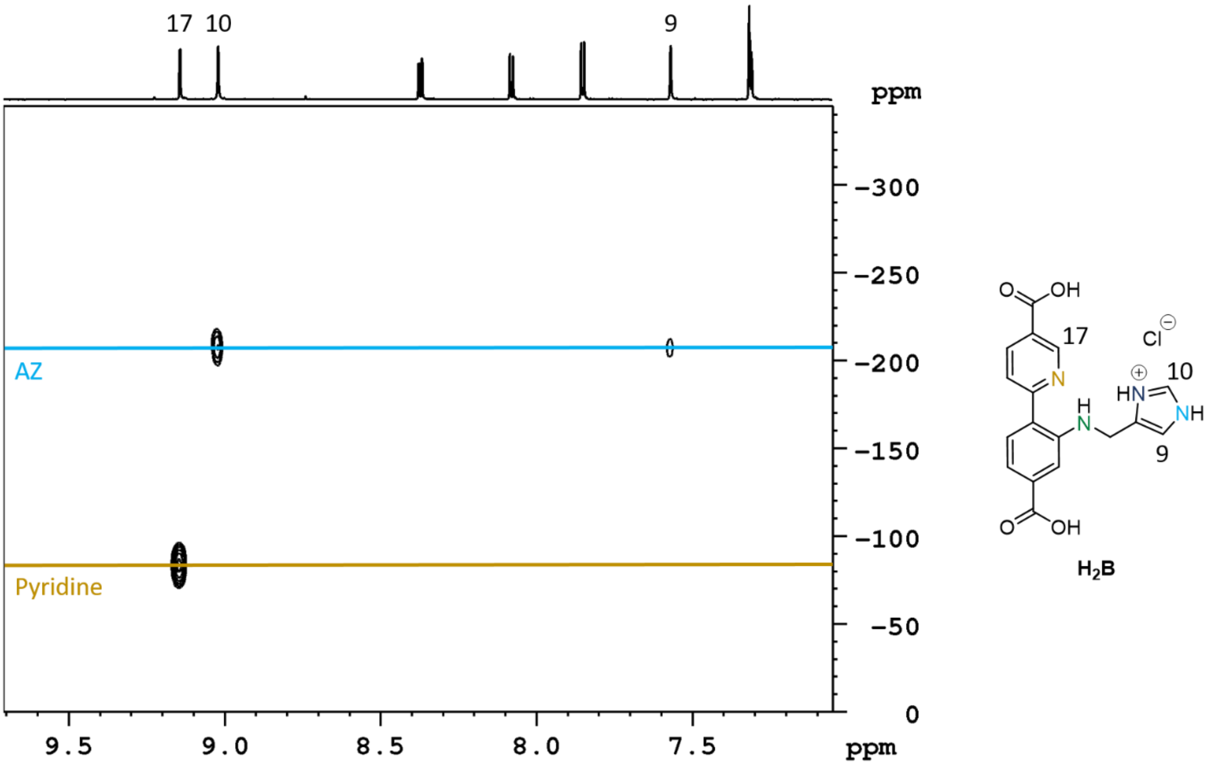

Figure S12. ^{1}H-^{15}N HMBC (800 MHz, d_6-DMSO) of H_2**1**.

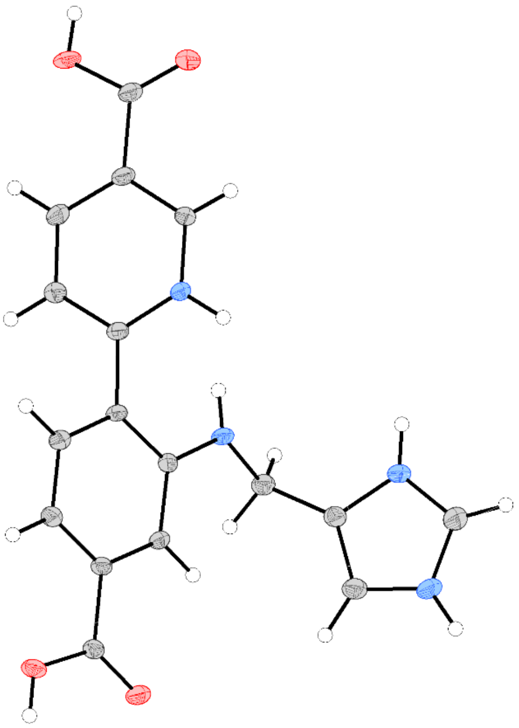

Figure S13. Single crystal XRD structure of H_2**1**·2HCl. Water molecules and chlorides (three each) were omitted for clarity. The crystal was obtained from the filtrate after acidification and does not represent the protonation state in the product described in this work.

Table S2. Crystal and refinement data for H_2**1** (CCDC 2105656).

Crystal data	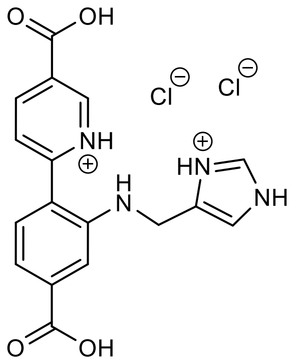
Chemical formula	$2(Cl)\cdot C_{17}H_{16}N_4O_4\cdot 3(H_2O)$
M_r	465.28
Crystal system, space group	Triclinic, $P\bar{1}$
Temperature (K)	100
a, *b*, *c* (Å)	9.4125 (5), 9.5825 (5), 12.6203 (7)
α, β, γ (°)	111.810 (1), 94.784 (1), 97.059 (1)
V ($Å^3$)	1038.46 (10)
Z	2
Radiation type	Mo *K*α
μ (mm^{-1})	0.36
Crystal size (mm)	0.4 × 0.25 × 0.17
Diffractometer	Bruker D8 Venture
Absorption correction	Multi-scan *SADABS2016*/2 (Bruker,2016/2) was used for absorption correction. wR2(int) was 0.1059 before and 0.0429 after correction. The Ratio of minimum to maximum transmission is 0.9312. The λ/2 correction factor is Not present.
T_{min}, T_{max}	0.694, 0.746
No. of measured, independent and observed [$I > 2\sigma(I)$] reflections	34513, 5156, 4594
R_{int}	0.029
$(\sin \theta/\lambda)_{max}$ ($Å^{-1}$)	0.668
Refinement	
$R[F^2 > 2\sigma(F^2)]$, $wR(F^2)$, S	0.033, 0.089, 1.06
No. of reflections	5156
No. of parameters	298
H-atom treatment	H atoms treated by a mixture of independent and constrained refinement
$\Delta\rho_{max}$, $\Delta\rho_{min}$ (e $Å^{-3}$)	0.43, -0.28

1.2 *Syntheses of the Molecular Copper Complexes*

Synthesis of [(Me$_2$**1**)$_2$Cu][OTf]

Me$_2$**1** (200 mg, 0.55 mmol) and CuOTf·4MeCN (206 mg, 0.55 mmol, 1 equiv.) were stirred overnight in MeCN (2 mL). The product was collected through filtration and washed with diethyl ether (2x 1 mL). After drying, [(Me$_2$**1**)$_2$Cu][OTf] was obtained as a bright yellow solid (152 mg, 0.16 mmol, 59 %).

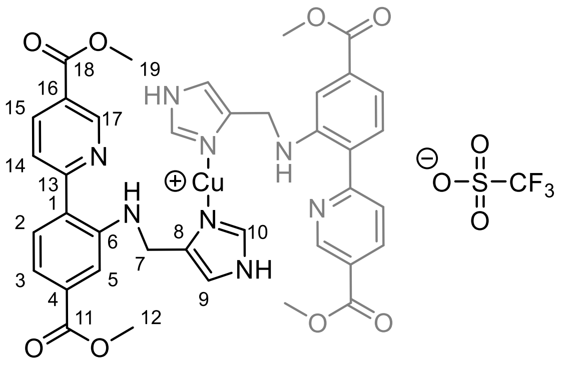

^{1}H NMR (800 MHz, d_6-DMSO): δ[ppm] 12.86 (2H, s, imidazole N**H**), 9.02 (2H, t, $^3J_{H,H}$ = 4.7 Hz, N**H**), 8.72 (2H, s, H17), 8.13 (2H, s, H15), 8.01 (2H, s, H10), 7.81 (2H, s, H14), 7.67 (2H, d, $^3J_{H,H}$ = 6.6 Hz, H2), overlap (4H) of 7.24 (*br,* s, H9) and 7.22 (s, H5), 7.17 (2H, d, $^3J_{H,H}$ = 7.8 Hz, H3), 4.34 (4H, $^3J_{H,H}$ = 3.4 Hz, H7), 3.89 (6H, s, H19), 3.81 (6H, s, H12). ^{13}C NMR (200 MHz, d_6-DMSO): δ[ppm] 166.0 (C11), 164.6 (C18), 160.7 (C13), 148.0 (C17), 147.0 (C6), 137.6 (C10), 137.3 (C15), 131.4 (C1), 129.9 (C2), 123.2 (C4), 122.8 (C16), 122.0 (C14), 120.6 (q, $^1J_{C-F}$ = 322 Hz, **C**F$_3$), 116.4 (C3), 112.3 (C5), 52.3 (C19), 52.1 (C12), 39.2 (C7, under solvent signal, detected with HSQC and DEPT). Missing: C8 and C9.^{15}N{^{1}H} NMR (800 MHz, d_6-DMSO): δ[ppm] –83.9 (N$_{Pyr}$), –172.6 (N$_{IM}$). The ^{15}N shifts were collected in two separate experiments (Figure S16 and Figure S17). ESI-MS: m/z 287.103 (100 % [Me$_2$Y+H]$^+$), 367.140 (45 %, [L+H]$^+$), 389.122 (38 %, [L+Na]$^+$), 428.054 (34 %, [L-H+Cu]$^+$), 794.185 (5 %, [M-H]$^+$); HRMS m/z [M-H]$^+$ ($C_{38}H_{36}CuN_8O_8^+$): Calcd: 794.1853 Found: 794.1868. Anal. Calcd: C, 49.55; H, 3.84; N, 11.85; Cu, 6.72 Found: C, 49.39; H, 3.88; N, 11.78; Cu, 6.67.

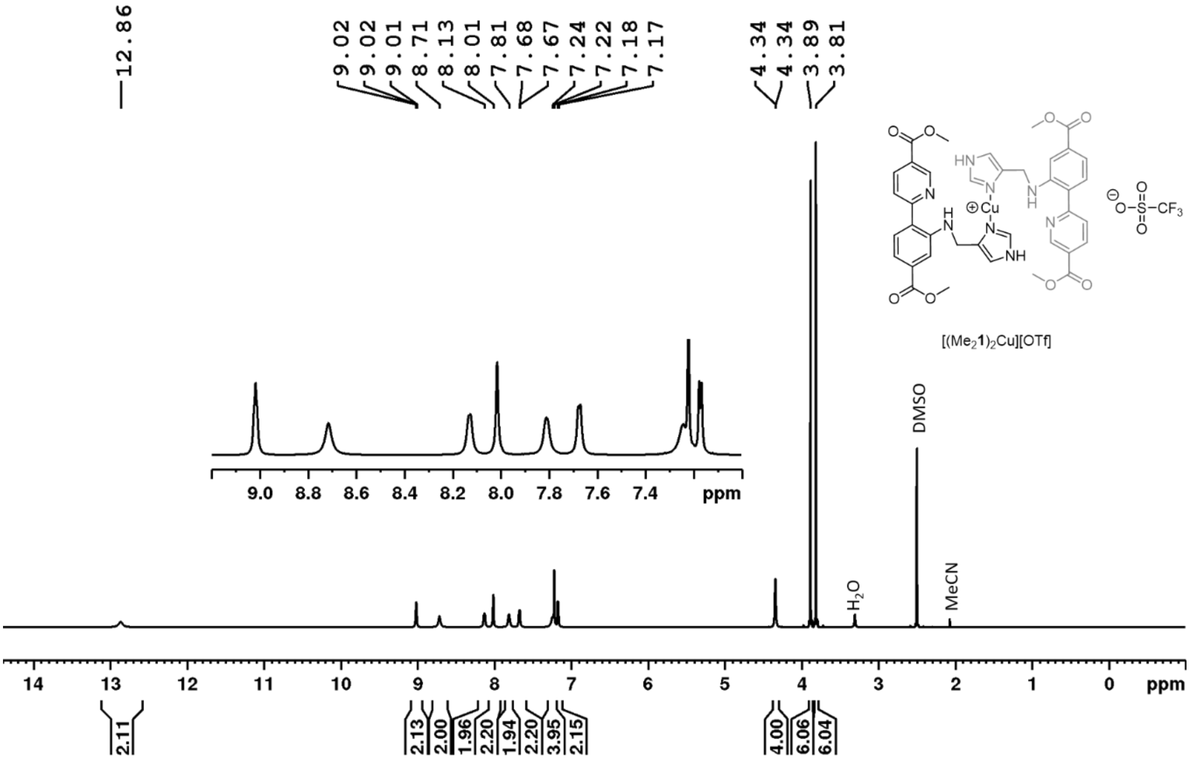

Figure S14. ^{1}H NMR spectrum (800 MHz, d_6-DMSO) of [(Me$_2$**1**)$_2$Cu][OTf].

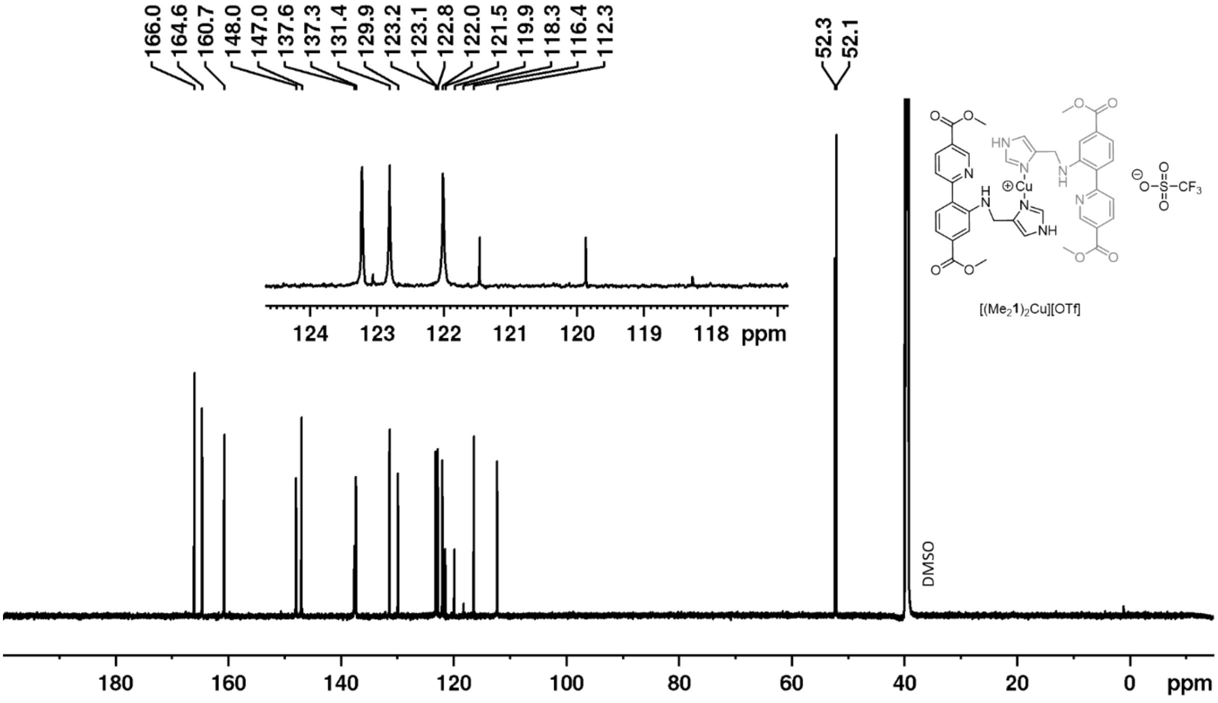

Figure S15. ^{13}C NMR spectrum (200 MHz, d_6-DMSO) of [(Me$_2$**1**)$_2$Cu][OTf].

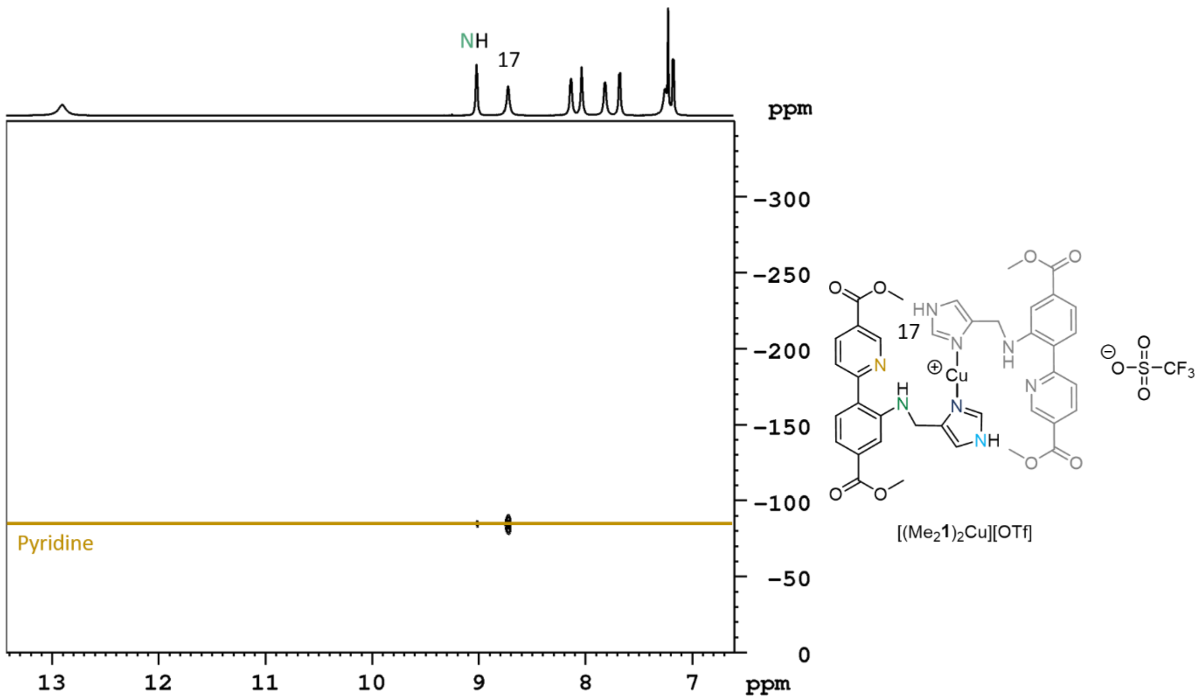

Figure S16. ^{1}H-^{15}N HMBC (800 MHz, d_6-DMSO) of [(Me$_2$**1**)$_2$Cu][OTf].

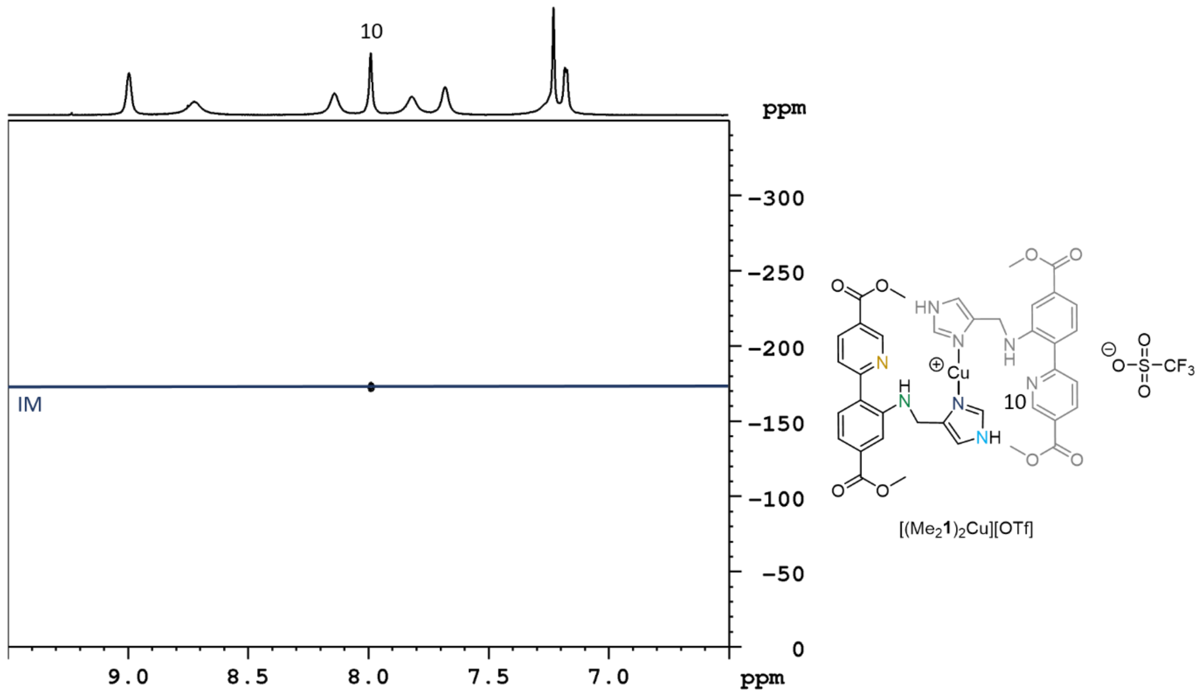

Figure S17. ^{1}H-^{15}N HMBC (800 MHz, d_6-DMSO) of [(Me$_2$**1**)$_2$Cu][OTf].

Synthesis of [(Me$_2$**1**)$_2$Cu][BF$_4$]

Me$_2$**1** (150 mg, 0.41 mmol) and CuBF$_4$·4MeCN (129 mg, 0.41 mmol, 1 equiv.) were stirred overnight in MeCN (2 mL). The product was collected through filtration and washed with diethyl ether (2x 1 mL). After drying, [(Me$_2$**1**)$_2$Cu][BF$_4$] was obtained as a bright yellow solid (Yield: 167 mg, 0.19 mmol, 93 %).

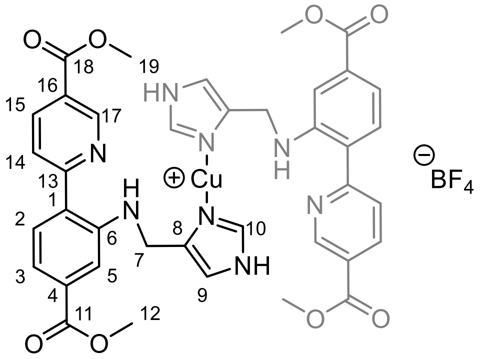

^{1}H NMR (800 MHz, d_6-DMSO): δ[ppm] 12.80 (2H, brs, imidazole NH), 9.01 (2H, brs, NH), 8.67 (2H, s, H17), 8.11 (2H, brs, H15), 7.99 (2H, s, H10), 7.78 (2H, s, H14), 7.65 (2H, H2), overlap (4H) of 7.28 (br, s, H9) and 7.22 (s, H5), 7.17 (2H, H3), 4.34 (4H, s, H7), 3.88 (6H, s, H19), 3.81 (6H, s, H12). ^{13}C NMR (200 MHz, d_6-DMSO): δ[ppm] 166.0 (C11), 164.6 (C18), 160.7 (C13), 148.0 (C17), 147.0 (C6), 137.7 (C10), 137.3 (C15), 131.4 (C1), 129.9 (C2), 123.3 (C4), 122.8 (C16), 122.0 (C14), 116.4 (C3), 112.3 (C5), 52.3 (C19), 52.1 (C12), 39.5 (C7, under solvent signal, detected with HSQC and DEPT). Missing: C8 and C9. ESI-MS: m/z 287.103 (100 % [Me$_2$Y+H]$^+$), 367.140 (77 %, [L+H]$^+$), 389.122 (44 %, [L+Na]$^+$), 429.062 (81%, [L+Cu]$^+$), 795.195 (24 %, [M]$^+$); HRMS m/z [M-H]$^+$ ($C_{38}H_{36}CuN_8O_8^+$): Calcd: 795.1947 Found: 795.1946. Elemental analysis (MP-AES): Calcd: 7.2 wt% Cu Found: 7.7±0.5 wt% Cu.

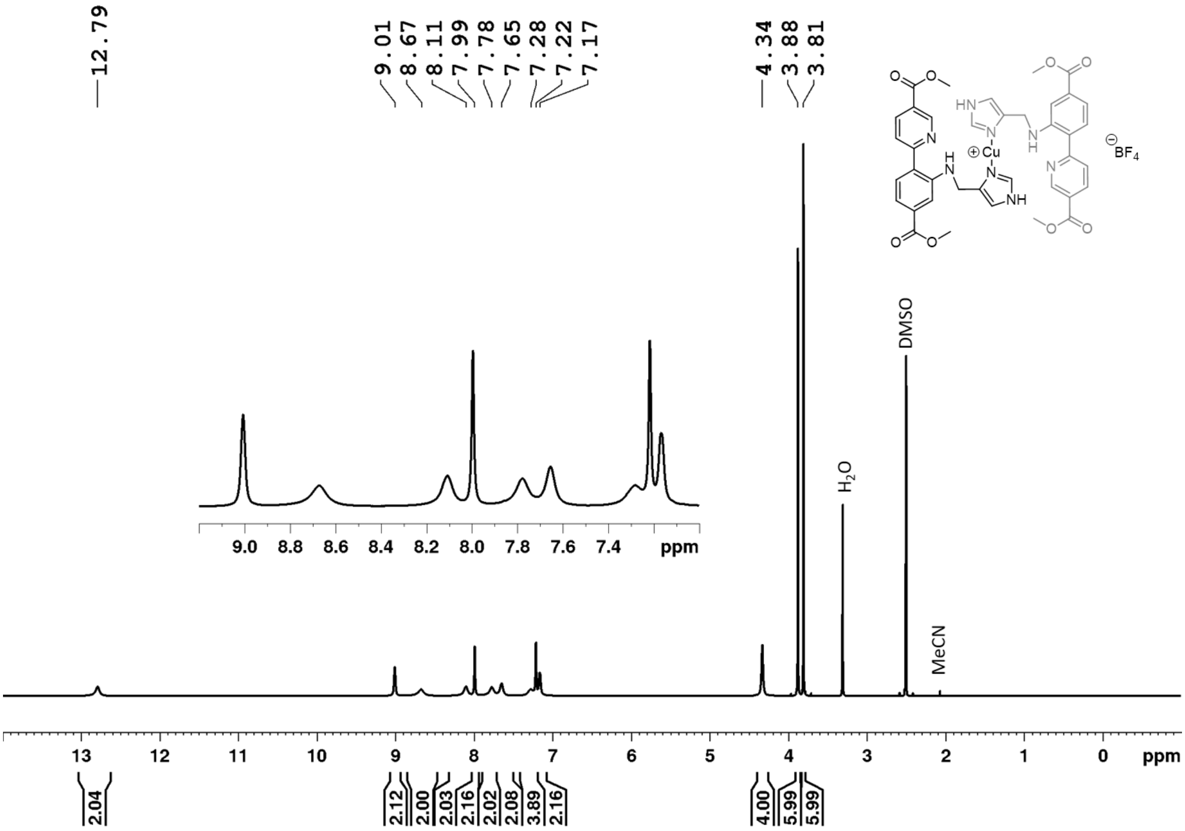

Figure S18. ^{1}H NMR spectrum (800 MHz, d_6-DMSO) of [(Me$_2$**1**)$_2$Cu][BF$_4$].

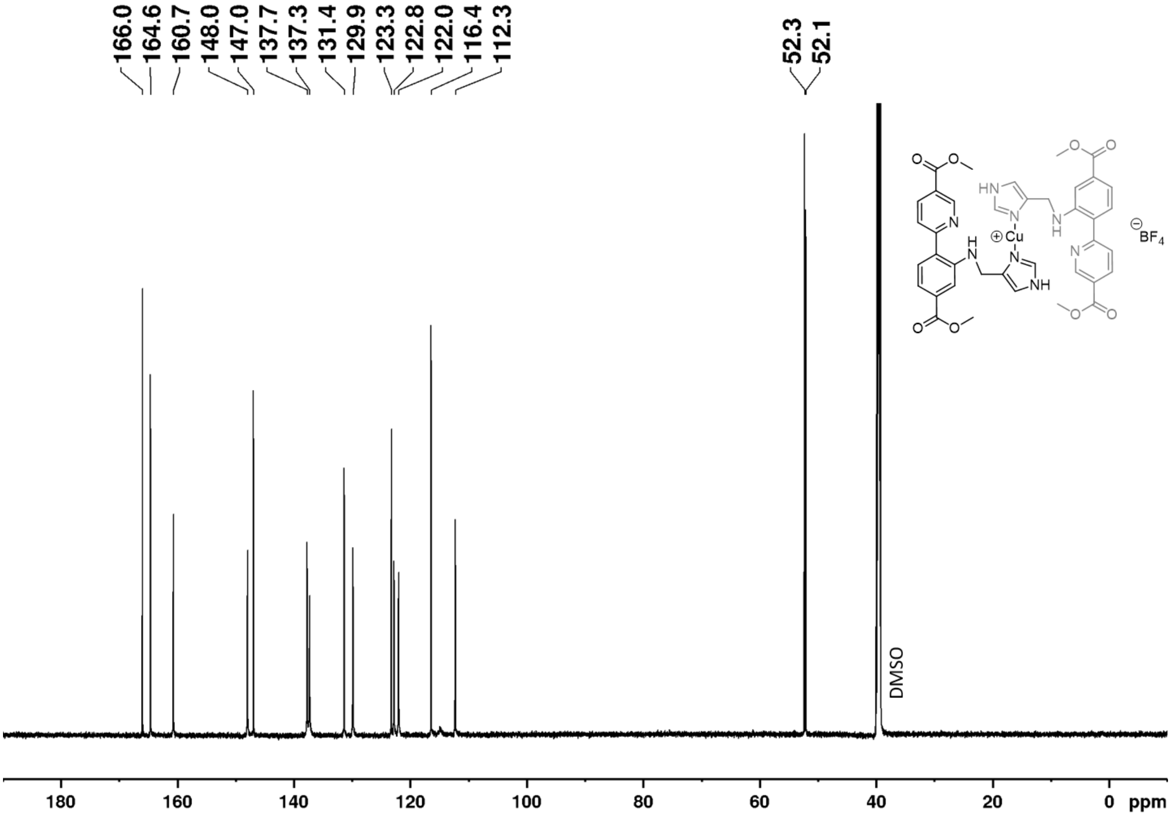

Figure S19. ^{13}C NMR spectrum (200 MHz, d_6-DMSO) of [(Me$_2$**1**)$_2$Cu][BF$_4$].

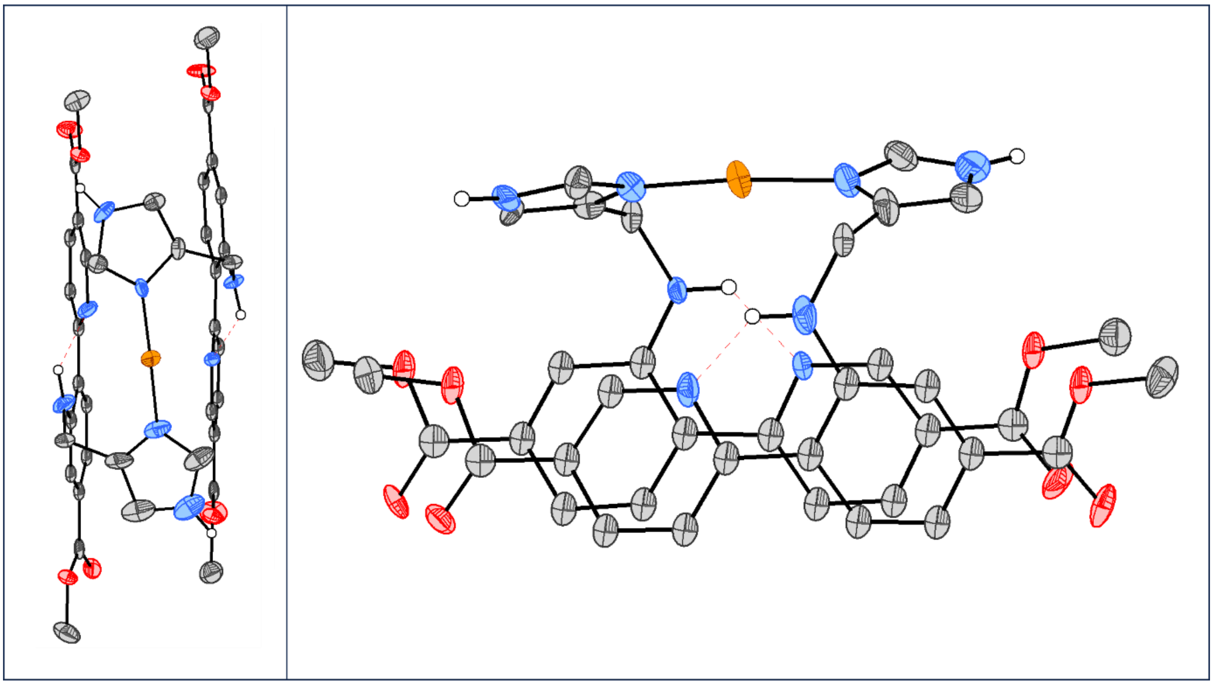

Figure S20. Single crystal XRD structure of [(Me$_2$**1**)$_2$Cu][BF$_4$] with ellipsoids at 50% probability (top and side view). Explicit modeling of the counter ion was not possible due to a high disorder and it was therefore treated with a solvent mask (see Table S3 for details).

Table S3. Crystal and refinement data for [(Me$_2$**1**)$_2$Cu][BF$_4$] (CCDC 2325932).

Crystal data	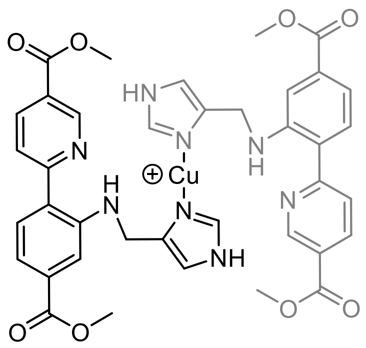
Chemical formula	$C_{38}H_{36}CuN_8O_8$
M_r	796.29
Crystal system, space group	Triclinic, $P\bar{1}$
Temperature (K)	100
a, *b*, *c* (Å)	7.0232 (12), 13.372 (2), 20.612 (3)
α, β, γ (°)	95.020 (7), 91.808 (9), 100.397 (9)
V (Å^3)	1894.5 (5)
Z	2

Radiation type	Mo *K*α
μ (mm^{-1})	0.64
Crystal size (mm)	1.18 × 0.13 × 0.05
Data collection	
Diffractometer	Bruker D8 Venture
Absorption correction	Multi-scan<br>SADABS2016/2 (Bruker,2016/2) was used for absorption correction. wR2(int) was 0.0909 before and 0.0592 after correction. The Ratio of minimum to maximum transmission is 0.8824. The λ/2 correction factor is Not present.
No. of measured, independent and observed [$I > 2\sigma(I)$] reflections	17194, 4059, 3176
R_{int}	0.069
θ_{max} (°)	21.0
$(\sin \theta/\lambda)_{max}$ (Å^{-1})	0.505
Refinement	
$R[F^2 > 2\sigma(F^2)]$, $wR(F^2)$, S	0.106, 0.244, 1.17
No. of reflections	4059
No. of parameters	332
H-atom treatment	H-atom parameters constrained
$\Delta\rho_{max}$, $\Delta\rho_{min}$ (e Å^{-3})	0.92, −0.93
Special Details	The sample scattered very weakly. Similar carbon and oxygen atom environments were constrained to have the same thermal parameteres using EADP, see _olex2_refinement_description section 3. Some carbon atoms were restrained to behave more isotropically using ISOR. The solvent mask removed 59 disordered electrons per unit cell, correspond roughly to the electron count of one BF4- counterion (42) and one acetonitrile solvent molecule (22) per unit cell."

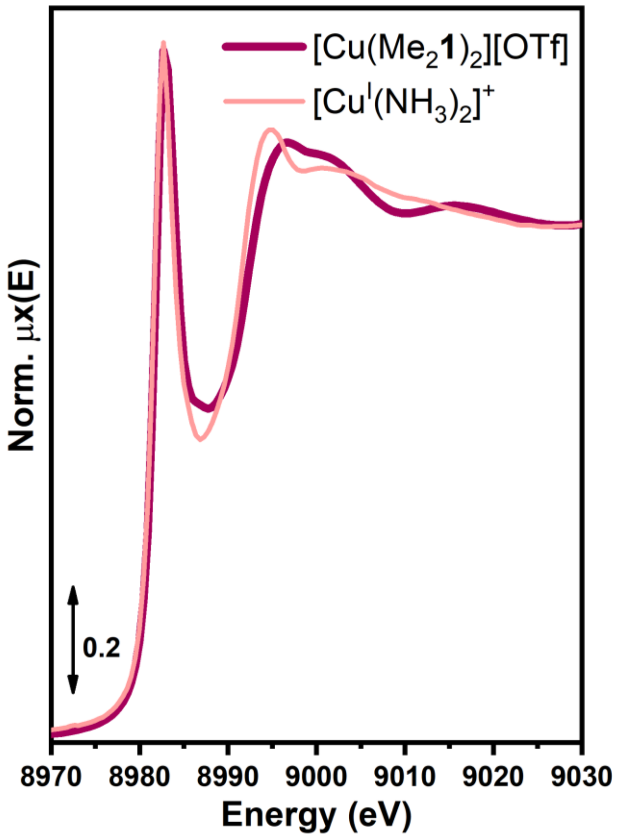

Figure S21. Comparison between Cu K-edge XANES spectra of $[Cu(Me_2\mathbf{1})_2][OTf]$ (solid, measured as a self-supporting pellet of optimized mass) with that of a $[Cu(NH_3)_2]^+$ model compound, measured previously in solution.[9]

Synthesis of [Me_2**1**Cu][BF_4]$_2$

Me_2**1** (200 mg, 0.546 mmol) was dissolved in CH_2Cl_2/MeOH (ca. 5 mL). A methanol solution of $Cu(BF_4)_2 \cdot 6H_2O$ (188 mg, 0.546 mmol, 1 equiv.) was added. The mixture was left to stir for 2 h. The solvent was removed under reduced pressure to obtain Me_2**1**$Cu(BF_4)_2$ (Yield: 315 mg, 0.522 mmol, 96%) as a green powder.

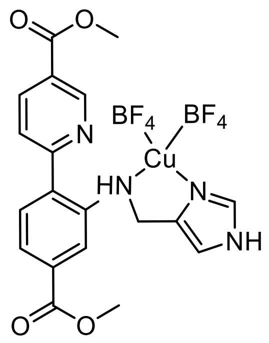

ESI-MS: m/z 287.103 (100 % [Me_2Y+H]$^+$), 367.140 (29 %, [L+H]$^+$), 389.122 (28 %, [L+Na]$^+$). Anal. Calcd: C, 37.81; H, 3.01; N, 9.28 Cu, 10.53 Found: C, 37.74; H, 3.01; N, 9.25; Cu, 10.51.

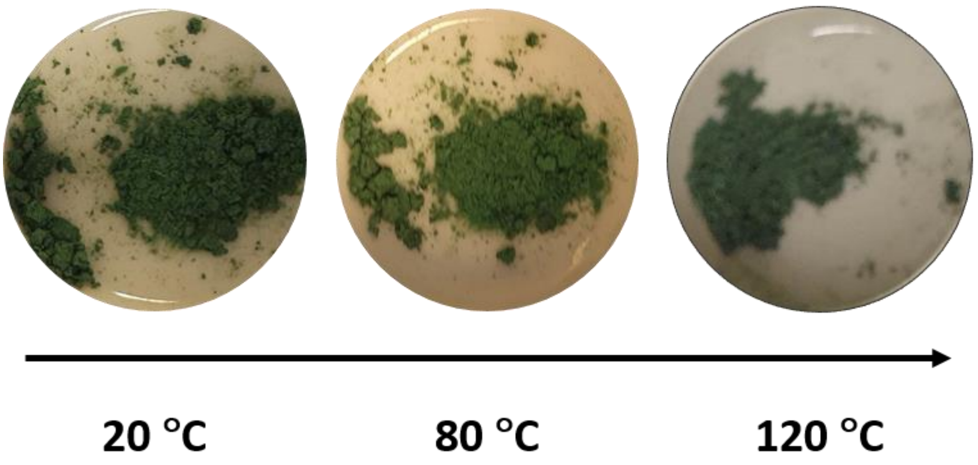

Figure S22. Complex Me_2**1**$Cu(BF_4)_2$ does not change its colour when heated in an oven.

Synthesis of Me_2**1**$CuCl_2$

Me_2**1** (100 mg, 0.273 mmol) and $CuCl_2$ (37 mg, 0.273 mmol, 1 equiv.) were stirred over night in MeOH (3 mL). The product was collected by filtration. After drying, a green solid was obtained (68 mg, 0.137 mmol, 50 %).

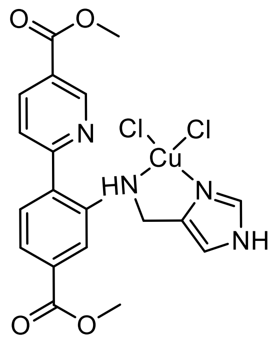

ESI-MS: m/z 287.103 (47 % $[Me_2Y+H]^+$), 367.140 (100 %, $[L+H]^+$), 389.122 (25 %, $[L+Na]^+$). Anal. Calcd: C, 45.57; H, 3.62; N, 11.19 Cu, 12.78 Found: C, 45.52; H, 3.59; N, 11.17; Cu, 12.72.

1.3 Syntheses of Metal Organic Frameworks

For the sake of obtaining the best possible data using various spectroscopic techniques, several samples with different copper loading were synthesized. The medium and high loading samples (ML and HL) have a comparable loading of **1**, *ca.* 1.2 per Zr_6-cluster, corresponding to a 20 % incorporation. The low loading sample has 0.3 **1**-linkers per Zr_6-cluster (*ca*. 5 % incorporation). Due to the paramagnetic nature of Cu(II), as well as the chance of forming insoluble metal complexes, quantification of **1** with ^{1}H NMR is not possible on the copper-incorporated materials. Incorporation of **1** was therefore assumed to remain unchanged between UiO-67-**1** and UiO-67-[**1**Cu][BF_4]$_2$.

<u>Synthesis of UiO-67-**1**</u>

Adapted from Fei and Cohen.[10]

H_2**1**·HCl (0.727 g, 1.94 mmol, 1.32 equiv.) was dissolved in 130 mL of KOH solution (4 %, aq.). The solution was then titrated with HCl solution (1 M, aq.) until pH = 7, before adding DMF (41 mL). UiO-67 (2.96 g, 1.47 mmol, 1.00 equiv.) was added to the clear, orange solution and was transferred to a suitable HDPE container and shaken continuously over night at room temperature. The following day, the mother liquor was removed after centrifugation and the solids were redispersed in water (100 mL). This was repeated two times, before displacing the water using acetone (100 mL). The material was washed a total of three times, before filtered and gently dried on the filter where it was washed one final time using diethyl ether (200 mL), yielding UiO-67**-1** ($Zr_6O_4(OH_4)(BPDC)_{4.7}$(**1**)$_{1.2}(OH/H_2O)_{0.1}$).

For the low-loading sample, 0.33 equiv. of H_2**1** was used instead.

<u>Synthesis of UiO-67-[**1**Cu][BF_4]$_2$-**LL**</u>

2.2 g of UiO-67-**1**-LL was added to a solution of $Cu(BF_4)_2{\cdot}6H_2O$ (0.088 g, 0.85 mol. equiv. to **1**) in acetonitrile (40 mL). The dispersion was stirred overnight at room temperature, before centrifuged. The product was then washed using acetonitrile (3 x 40 mL), and acetone (1x 40 mL). Lastly, the product was filtered and rinsed using diethyl ether (20 mL) on the filter where it was gently dried, yielding compound UiO-67-[**1**Cu][BF_4]$_2$-LL with the estimated composition $Zr_6O_4(OH)_4(BPDC)_{5.1}$(**1**)$_{0.3}(Cu(BF_4))_{0.2}$.

<u>Synthesis of UiO-67-[**1**Cu][BF_4]$_2$-**ML**</u>

10 g of UiO-67-**1**-ML was added to a solution of $Cu(BF_4)_2{\cdot}6H_2O$ (1.54 g, 0.85 mol. equiv. to **1**) in acetonitrile (200 mL). The dispersion was stirred overnight at room temperature, before centrifuged. The product was then washed using acetonitrile (3 x 200 mL), and acetone (1x 200 mL). Lastly, the product was filtered and rinsed using diethyl ether (100 mL) on the filter where it was gently dried, yielding compound UiO-67-[**1**Cu][BF_4]$_2$-ML with the estimated composition $Zr_6O_4(OH)_4(BPDC)_{4.6}$(**1**)$_{1.2}(Cu(BF_4))_{1.2}$.

Synthesis of UiO-67-[**1**Cu][BF_4]$_2$-**HL**

Approximately 1.40 g UiO-67-**1**-HL (2.035 g wet, equivalent to 0.8 mmol **1**)* was added to a solution of $Cu(BF_4)_2 \cdot 6H_2O$ (0.320 g, 0.927 mmol, 1.38 eqv. to **1**) in acetonitrile (40 mL). The reaction mixture was shaken continuously for two days at room temperature, before washed with acetonitrile (200 mL), ethanol (3 x 200 mL) and diethyl ether (200 mL). The green solid was separated by filtration and gently dried on the filter, yielding compound UiO-67-[**1**Cu][BF_4]$_2$-HL with the estimated composition $Zr_6O_4(OH)_4(BPDC)_{4.7}$(**1**)$_{1.2}$$(Cu(BF_4)_2)_{1.2}$.

* Residual solvent content of UiO-67-**1** was estimated to be roughly 30 % of its total mass using thermogravimetry. The sample was not dried prior to the metalation, and the estimated weight of dry MOF was therefore 1.40 g. Furthermore, from the approximate chemical formula there are 1.2 **1** linkers per unit MOF, giving rise to the 0.8 mmol **1** linkers available).

Removing Residual H_2BPDC

The unexpected manifestation of free H_2BPDC was observed in the medium and high loading samples in the powder X-ray diffraction patterns, present only after metalation was performed. (Figure S24B and C) The amounts present were not quantified, but were shown to be removable by washing with DMF. (Figure S24D)

0.20 g of UiO-67-[**1**Cu][BF_4]$_2$ was dispersed in 4 mL DMF, and was continuously agitated for 2 hours at room temperature. The solids were separated *via* centrifugation (4000 rpm, 5 min) and redispersed into acetone. This was repeated once, before filtered and rinsed with diethyl ether (10 mL) on the filter, while allowing it to gently dry.

Powder X-ray Diffraction

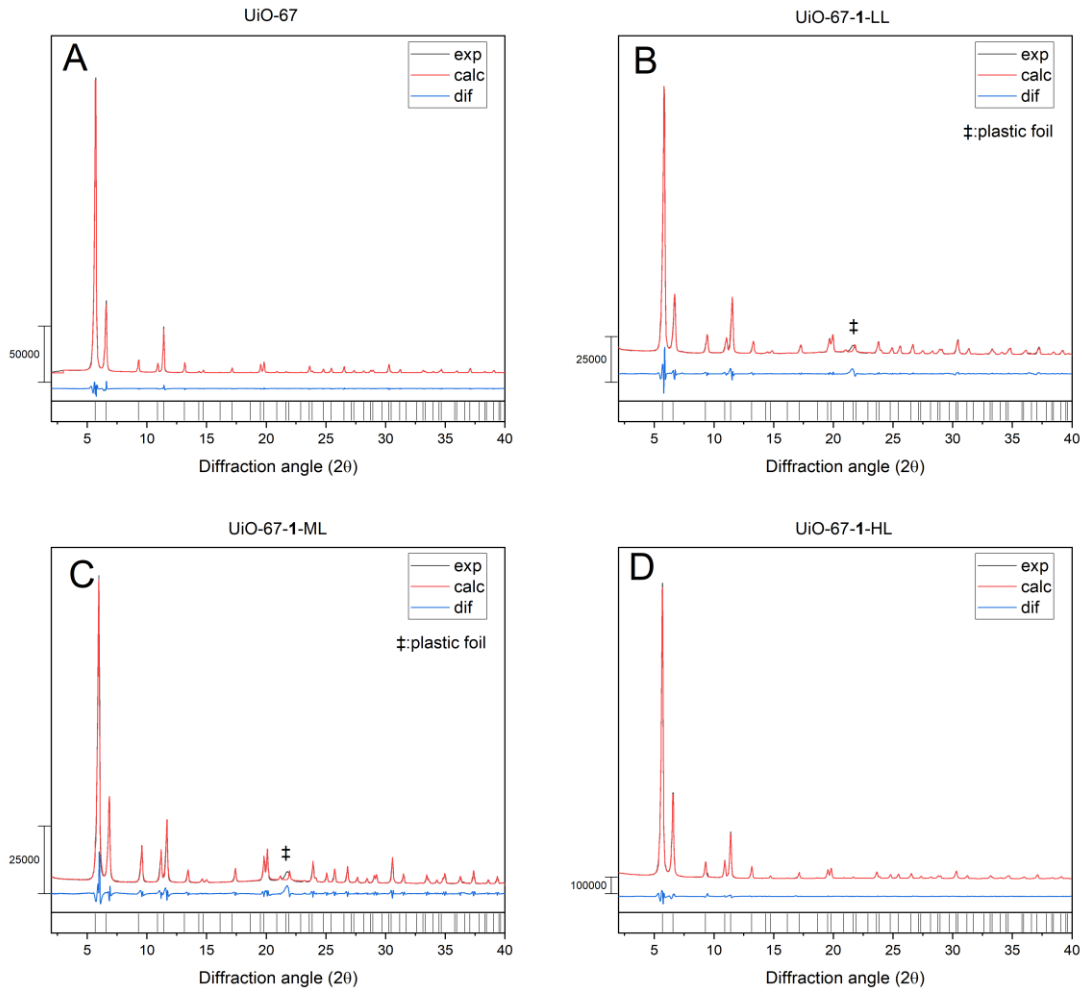

Figure S23. Powder X-ray diffraction patterns (black) of the parent material UiO-67 as received (**A**), and the three post-synthetically linker exchanged materials. **B**: LL, **C**: ML and **D**: HL. Plastic foil has been used to prepare some of the samples (seen as a broad peak at 21.8°). All patterns have been Pawley fit (red), with the differences shown in blue. The calculated reflections for UiO-67 are also shown below.

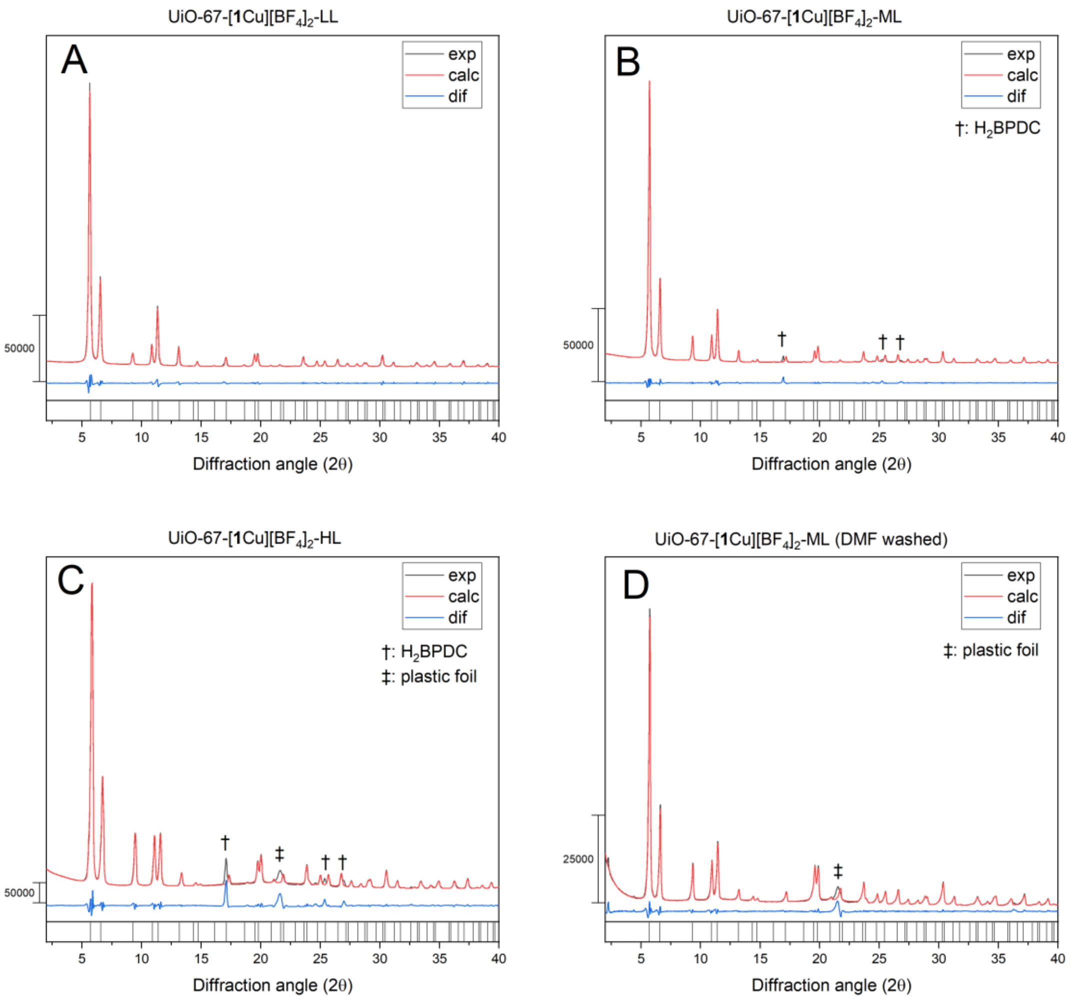

Figure S24. Powder X-ray diffraction patterns (black) of the metallated materials. **A**: UiO-67-[**1**Cu]$[BF_4]_2$-LL, **B**: UiO-67-[**1**Cu]$[BF_4]_2$-ML, **C**: UiO-67-[**1**Cu]$[BF_4]_2$-HL, **D**: DMF washed UiO-67-[**1**Cu]$[BF_4]_2$-ML. Plastic foil has been used to prepare some of the samples (seen as a broad peak at 21.8°). All patterns have been Pawley fit (red), with the differences shown in blue. The calculated reflections for UiO-67 are also shown below.

Variable Temperature pXRD of UiO-67-[1Cu][BF_4]$_2$-**HL**

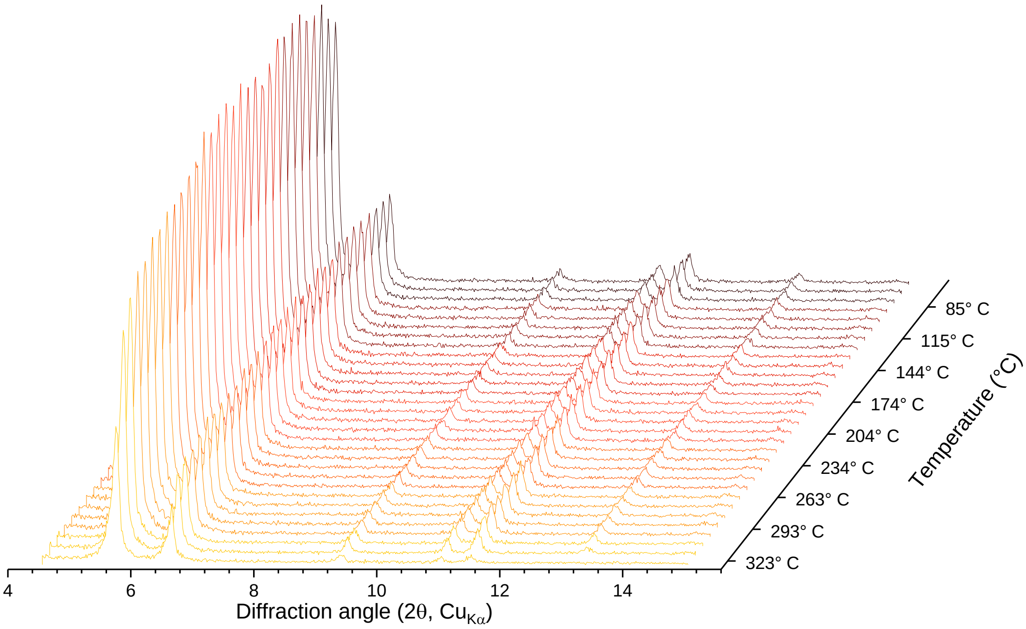

Figure S25. Variable temperature powder X-ray diffraction (VT-PXDR) of sample UiO-67-[**1**Cu][BF_4]$_2$-HL. Measured in a sealed capillary tube with a heating rate of 5 °Cmin^{-1}.

Digestion ^{1}H NMR of Metal-Organic Frameworks

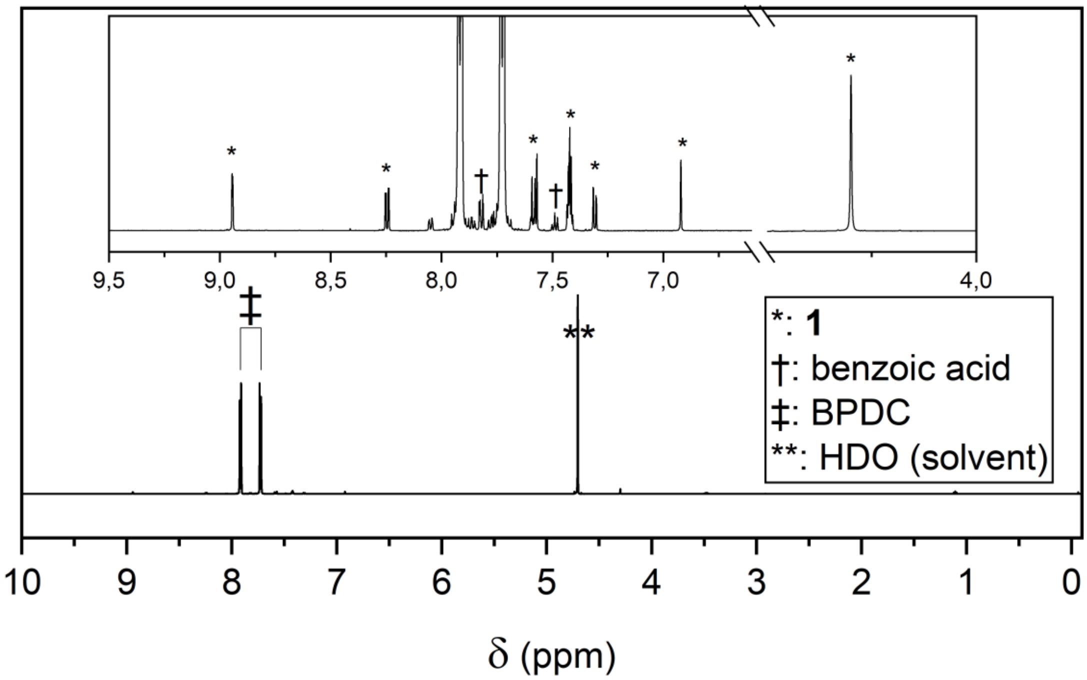

Figure S26. Representative ^{1}H NMR spectrum (600 MHz, 1 M NaOD in D_2O) of digested UiO-67-**1**-ML. Sample contains residual diethyl ether from washing.

Table S4. NMR integrals for the parent UiO-67 and the linker exchanged materials.

Sample	∫ **1***	∫ BPDC†	∫ benzoate‡
UiO-67	-	4	0.076
UiO-67-**1**-LL	0.057	4	0.026
UiO-67-**1**-ML	0.251	4	0.019
UiO-67-**1**-HL	0.0266	4	0.053

*: Integrated doublet of doublets at 8.24 ppm (1H)

†: Integrated peak at 7.91 ppm (4H)

‡: Integrated peak at 7.48 ppm (1H)

Thermogravimetric Analysis

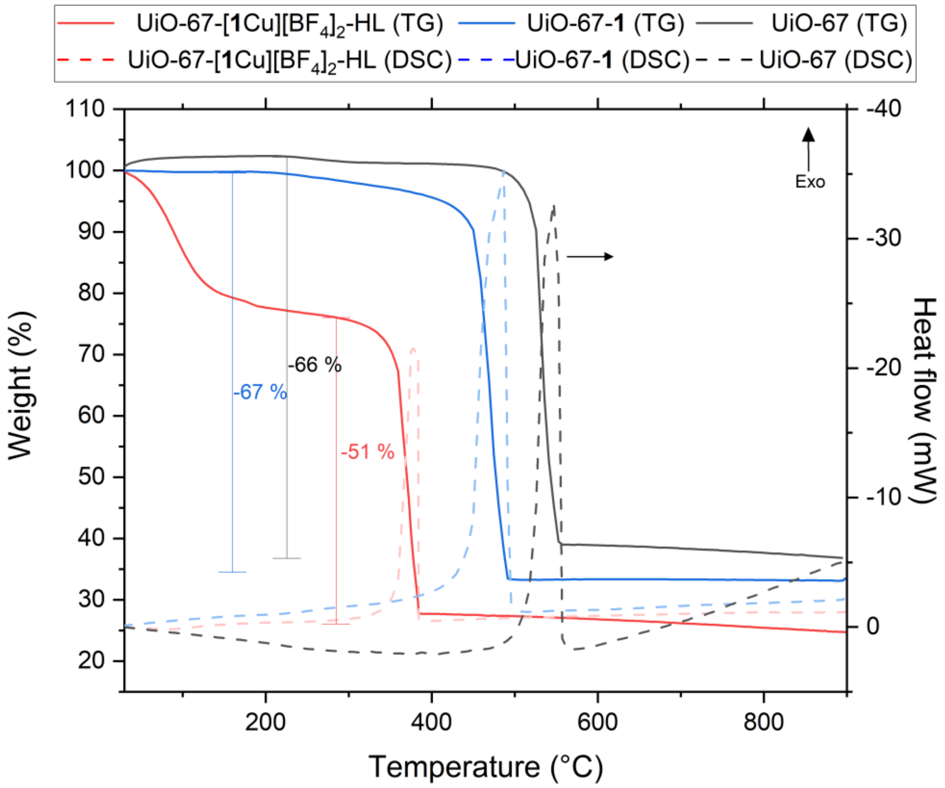

Figure S27. Thermogravimetric analysis of UiO-67-[**1**Cu][BF4]2-HL (red) and UiO-67-**1**-HL (blue) and UiO-67 (black). Solid lines are TG while dashed lines indicate the DSC signal.

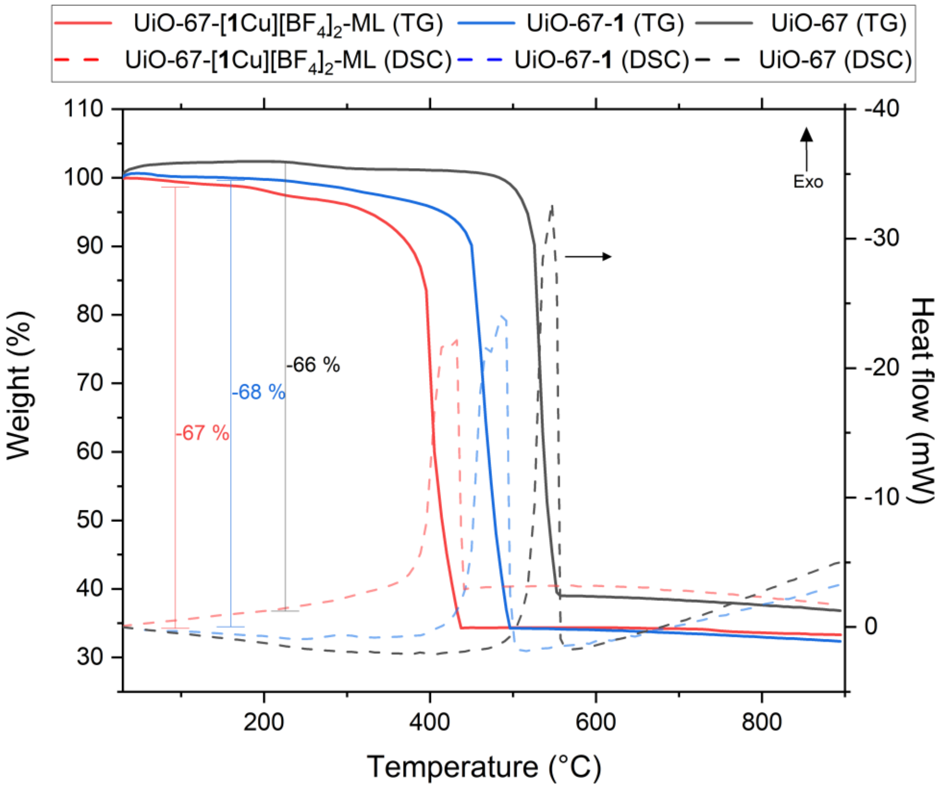

Figure S28. Thermogravimetric analysis of UiO-67-[**1**Cu][BF_4]$_2$-ML (red) and UiO-67-**1**-ML (blue) and UiO-67 (black). Solid lines are TG while dashed lines indicate the DSC signal.

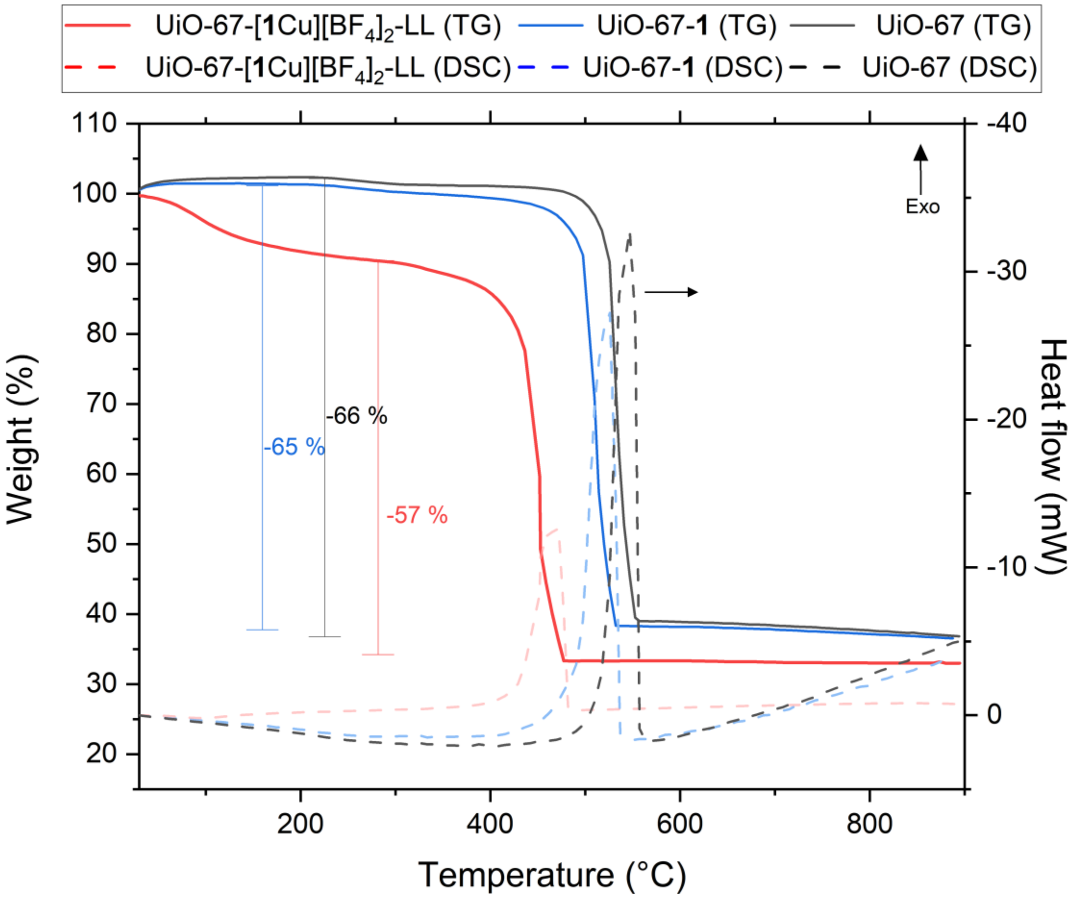

Figure S29. Thermogravimetric analysis of UiO-67-[**1**Cu][BF_4]$_2$-LL (red) and UiO-67-**1** (blue)-LL and UiO-67 (black). Solid lines are TG while dashed lines indicate the DSC signal.

Energy Dispersive X-ray Spectroscopy (EDS)

Table S5. Fluorine to copper ratio for UiO-67-[**1**Cu][BF_4]$_2$-**HL**, as given by EDS analysis.

Sample	F/Cu-ratio
UiO-67-[**1**Cu][BF_4]$_2$-HL	7.87 ± 0.06

Estimation of MOF Composition

The chemical composition of UiO-67-**1** was estimated using previously published methodology,[11] on the basis of thermogravimetry and digestion NMR results.

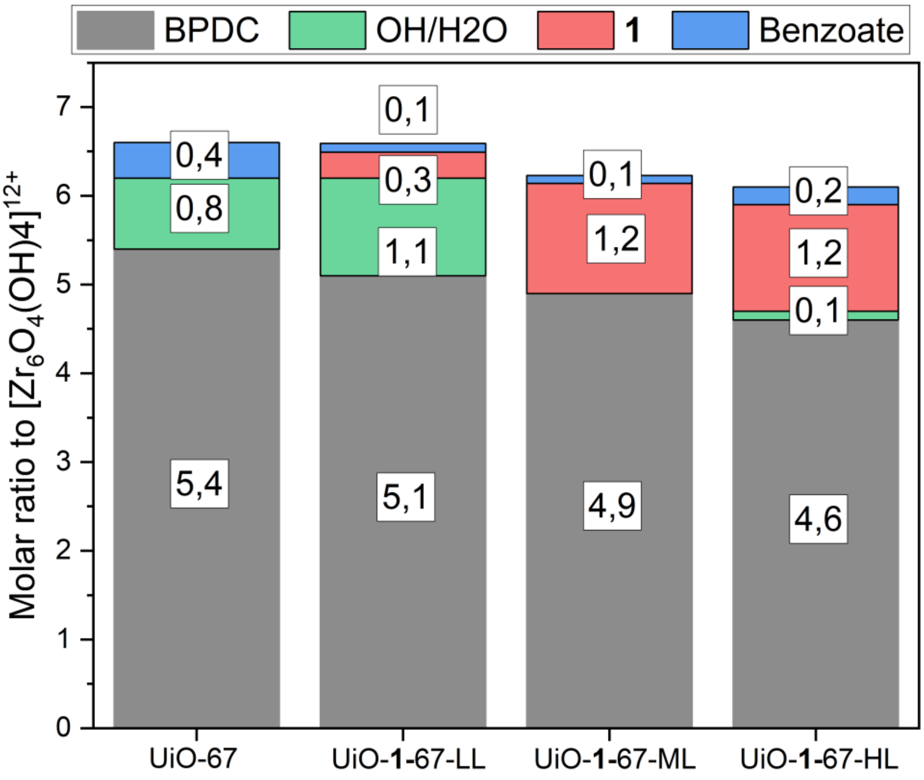

Figure S30. Chemical composition of the parent material UiO-67 and the post-synthetically linker exchanged UiO-67-**1**. The values are given relative to one $[Zr_6O_4(OH)_4]^{12+}$ cluster.

Elemental Analysis

Table S6. Combustion elemental analysis results for medium loaded sample.

Sample	Element [%]					
	C	H	N	Zr	F	Cu
UiO-67-**1**	44.29	4.12	3.87	13.71		
UiO-67-[**1**Cu][BF_4]$_2$-ML	40.39	3.49	3.65	11.87	7.21	
UiO-67-[**1**Cu][BF_4]$_2$-ML *dried*	42.58	3.96	4.11	13.45	6.33	3.05

Microwave Plasma Atomic Emission Spectroscopy (MP-AES)

Three replicates of UiO-67-[**1**Cu][BF_4]$_2$, consisting of 10 mg each, were digested in 500 µL of conc. H_2SO_4 at 70 °C overnight. Then, 200 µL H_2O_2 (aq., 30 wt.%) was added while still warm (**Caution**: highly exothermic), before diluted to 50 mL using H_2O. The solutions were filtered using a 0.22 µm syringe filter prior to measurement.

The samples were analyzed using an Agilent 4100 MP-AES instrument, using λ_{Zr} = 339.198 nm and λ_{Cu} = 327.395 nm.

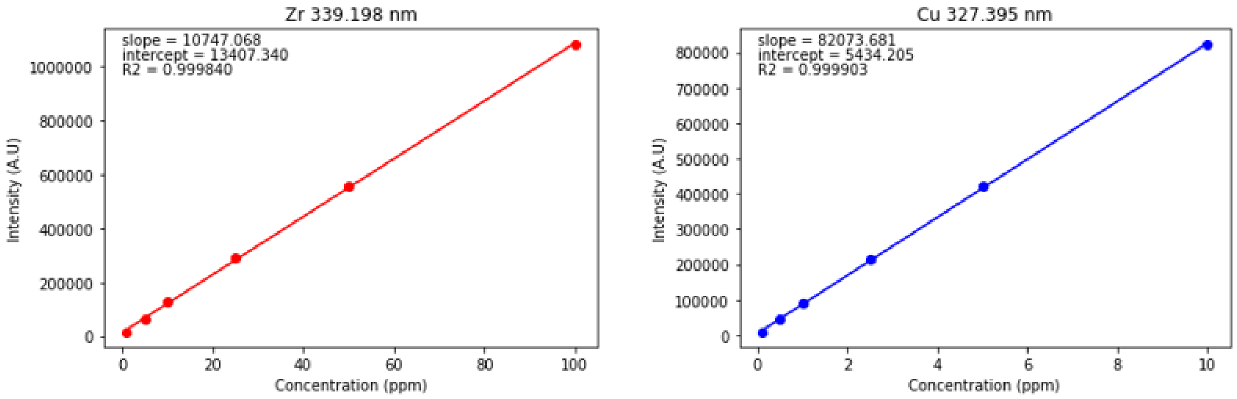

Figure S31. Calibration curves used for determination of Zr (left, red) and Cu (right, blue) concentrations.

Table S7. Copper to zirconium ratios for metallated samples, given by atomic emission spectrometry.

Sample	Cu/Zr_6 -ratio
UiO-67-[**1**Cu][BF_4]$_2$-LL	0.2020 ± 0.0009
UiO-67-[**1**Cu][BF_4]$_2$-ML	0.780 ± 0.004
UiO-67-[**1**Cu][BF_4]$_2$-HL	1.191 ± 0.007

N_2 Adsorption

Pretreatment was performed using a BELprep VAC I vacuum degasser. UiO-67-**1** was pretreated at 120 °C under high vacuum (< 10^{-3} mbar) for 24 hours prior to the measurement. UiO-67-[**1**Cu][BF_4]$_2$ was pretreated at 80 °C under similar vacuum conditions. The samples were then measured using a MicroBEL BelMini II at 77 K.

Table S8. Specific surface areas and pore volume for all metal-organic framework materials, measured by isothermal N_2 adsorption at 77 K.

**Sample**	**Surface area†**	**Pore volume‡**
	[m^2g^{-1}]	[cm^3g^{-1}]
UiO-67	2329	0.92
UiO-67-**1-LL**	2273	0.90
UiO-67-**1-ML**	1927	0.79
UiO-67-**1-HL**	1707	0.71
UiO-67-[**1**Cu][BF_4]$_2$-**LL**	2183	0.91
UiO-67-[**1**Cu][BF_4]$_2$-**ML**	1730	0.74
UiO-67-[**1**Cu][BF_4]$_2$-**HL**	1552	0.72

†: Calculated with BET theory

‡: Measured at 0.8 p/p_0

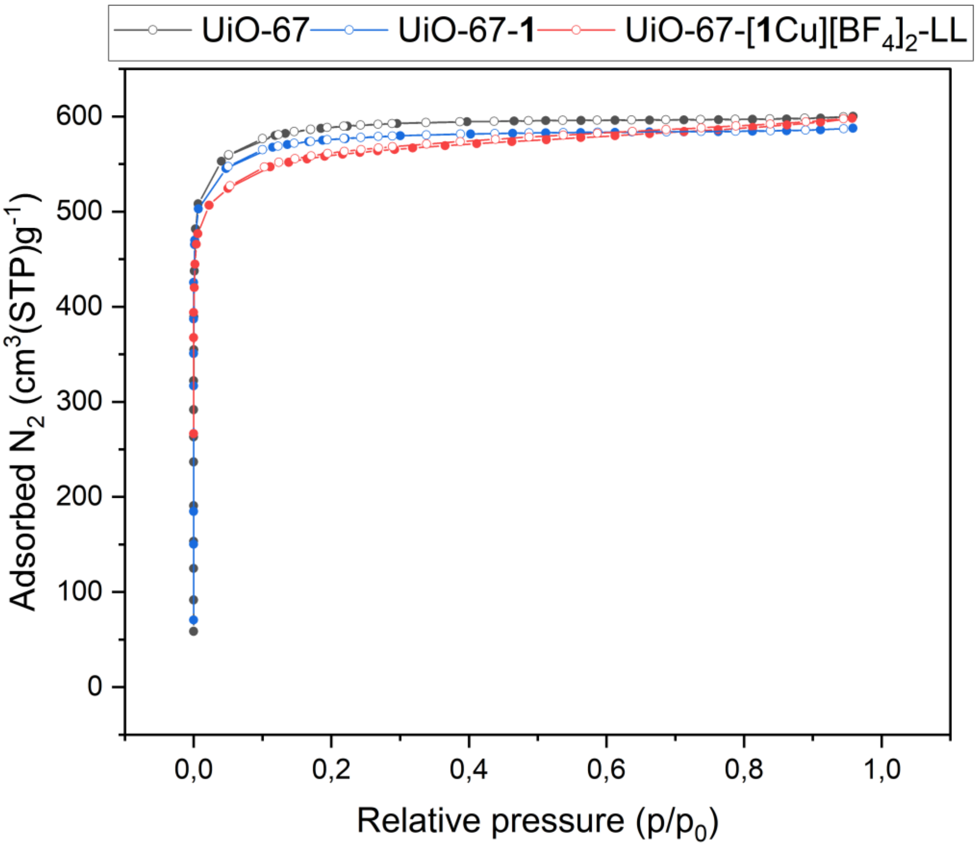

Figure S32. Nitrogen adsorption isotherm for compound UiO-67 (black), UiO-67-**1-LL** (blue) and UiO-67 [**1**Cu][BF_4]$_2$-**LL** (red). Desorption points are marked with open symbols.

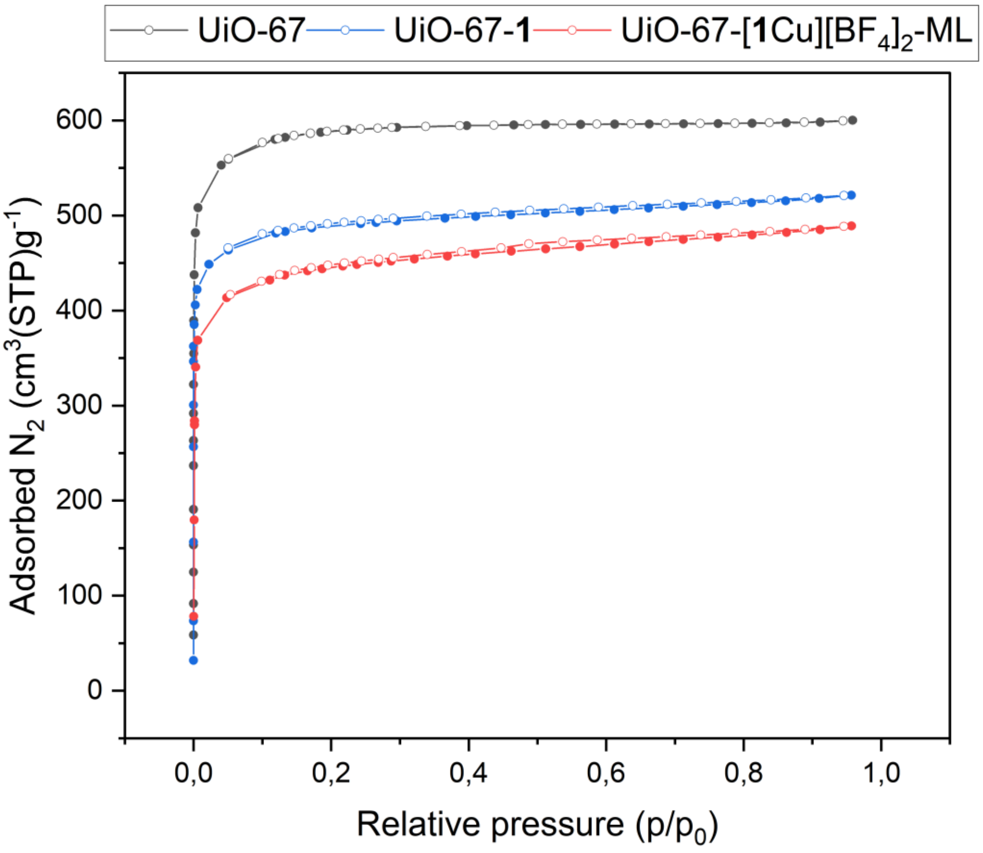

Figure S33. Nitrogen adsorption isotherm for compound UiO-67 (black), UiO-67-**1-ML** (blue) and UiO-67-[**1**Cu][BF_4]$_2$-**ML** (red). Desorption points are marked with open symbols.

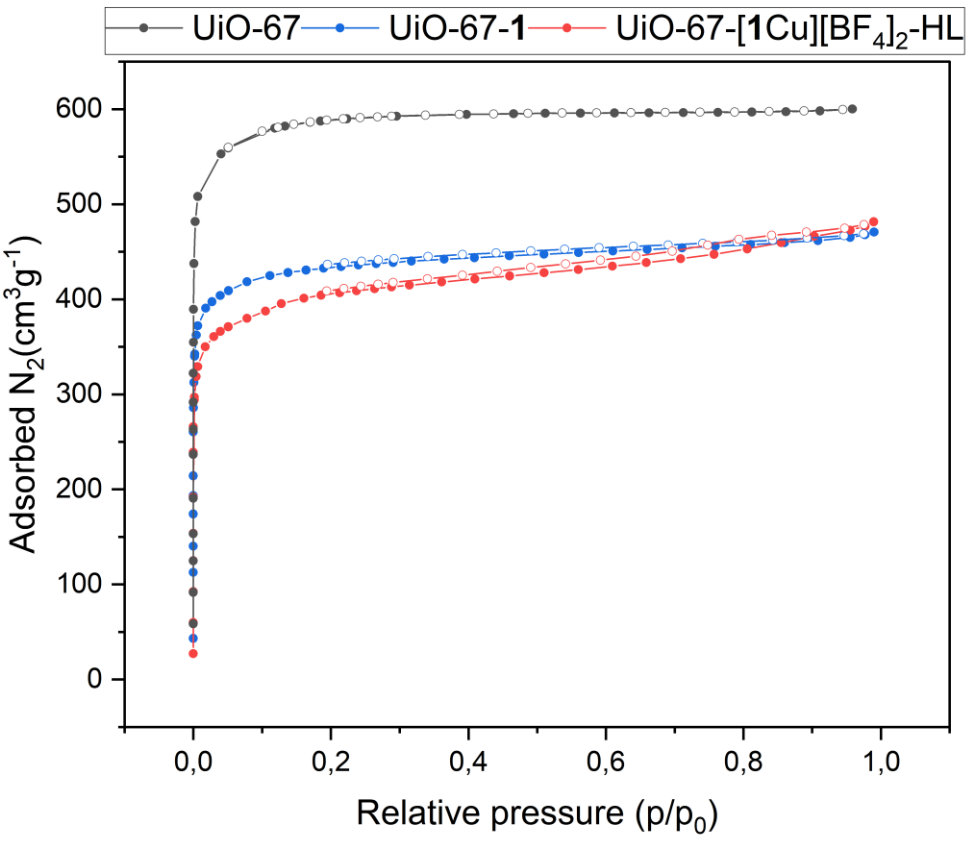

Figure S34. Nitrogen adsorption isotherm for compound UiO-67 (black), UiO-67-**1**-**HL** (blue) and UiO-67-[**1**Cu][BF_4]$_2$-**HL** (red). Desorption points are marked with open symbols.

2. X-Ray Absorption Spectroscopy

2.1 EXAFS Fitting for as-prepared UiO-67-1[Cu(BF$_4$)$_2$]-ML and -HL

EXAFS Fitting Model

EXAFS fitting for as-prepared HL and ML samples, measured at RT, was performed in R-space, in the $\Delta R = 1.0 – 4.0$ Å range, on the FT of the k^2-weigthed $\chi(k)$ EXAFS spectrum transformed in the 2.5–12.0 Å^{-1} range, resulting in 18 independent points ($2\Delta k \Delta R/\pi > 18$). Phases and amplitudes were computed by the FEFF6 code using the Artemis software from the Demeter package.[2, 13] To limit the number of free variables, all the included single scattering (SS) and multiple scattering (MS) paths have been refined with a common passive amplitude reduction factor (S_0^2) and energy shift (ΔE) parameters. As starting guess for the fit, we build a molecular structure of Cu(II) local environment in the UiO-67-[**1**Cu][BF4]2 MOF designed based on the available multi-technique characterization results. The local environment of Cu (see Figure S35a) includes in the first shell three N atoms from linker **1** (**N**, $\langle R_N \rangle = 2.03$ Å from DFT) and two O atoms from H_2O molecules (**O**, $\langle R_O \rangle = 2.25$ Å from DFT). The second shell includes the six C atoms of the B linker closer to the Cu centre (**C**, $\langle R_C \rangle = 2.94$ Å from DFT). The corresponding SS paths were parametrized with independent radial shifts (ΔR_i, i=N, O, C), Debye-Waller (DW) factors (σ^2_i, i=N, O, C), and coordination numbers N_i fixed according to the optimized geometry for the **HL** sample. Based on experimental data comparison and test EXAFS fits, for the **ML** sample, N_O was set to 1, while all the other coordination numbers were unchanged with respect to the theoretical model. The numerous SS and MS paths contributing in the 3.0–4.0 Å range were modelled considering a common contraction/expansion factor α_{high-R} and DW factor σ^2_{high-R} increasing as the square root of the distance $R_{eff,i}$ of the ith scattering atom from the absorber ($\Delta R_{high-R,i} = \alpha_{high-R} (R_{eff,i}/R_0)$; $\sigma^2_{high-R,i} = ss_{high-R}(R_{eff,i}/R_0)^{1/2}$, where R_0 indicates the shortest scattering path of the group).[6, 12] It is worth to note that the H atoms belonging the H_2O ligands and to linker **1** are actually 'EXAFS silent', due to their very low back-scattering amplitude. Consequently, the H atoms has been omitted in the model used as input for EXAFS analysis. For the same reason, the discrimination between H_2O and OH ligands from EXAFS is challenging, and we could not exclude substitution of the water molecule(s) considered in the model with hydroxyl group(s).

EXAFS Fit Results

The best-fit and experimental spectra for as-prepared UiO-67-[**1**Cu][BF$_4$]$_2$ are compared in Figure S35b,c and Figure S35d,e for the **HL** and **ML** samples, respectively. Table S9 reports an overview of the best-fit values for the optimized parameters in the two samples. Overall, a good fit is obtained in both cases, providing physically meaningful values for all the refined parameters.

Consistently with XANES evidence (see Figure 5, main text**)**, EXAFS analysis confirms that the average local environment for Cu(II) centers in the as-prepared **HL** MOF is consistent with five-fold coordinated sites, including 3 N from linker **1** and two extra O, plausibly from H_2O/OH ligands. Importantly, the high-R region is properly reproduced

by the adopted model, supporting the successful incorporation of Cu centers in the MOF scaffold. With respect to the first-shell environment, we observed a substantial improvement of the fit employing in the initial input only the shorter Cu-N and Cu-O SS paths among those predicted by DFT (i.e., R_N = 1.92 Å and R_O = 2.18 Å), associated with cumulative N_N = 3 and N_O = 2, respectively. The results reported in Figure S35 and Table S9 are indeed obtained under this fitting configuration. This points out a less distorted coordination environment with respect to that predicted theoretically, with a general shortening of EXAFS-refined average interatomic bond distances. Possibly, this could be related to the presence of additional physisorbed water/solvent molecules in the MOF pores under our experimental conditions, indirectly influencing the ligands arrangement and distance distribution in the first coordination sphere of Cu-centers.

Not surprisingly, a test fit performed applying the same fitting model (including N_O set to 2) to the **ML** sample resulted in unphysically high value of $\sigma^2_O \approx 0.02$ Å^2. Consistently with the qualitative comparison of XAS data for **HL** and **ML** samples reported in Figure 5 (main text), this result underlines an excessively high coordination number for the O coordination shell set in the model, artificially compensated by increasing the corresponding DW factor. Conversely, by setting N_O =1, a very good fit is achieved (corresponding to the curves and values reported in Figure S35d,e and Table S9 for UiO-67-[**1**Cu][BF_4]$_2$-**ML** which supports the preference for four-fold coordinated Cu(II) centers in this case. Notably, the refined interatomic distances are very similar for HL and ML samples, with a global slight elongation of Cu-N and Cu-O distances (barely outside the fit error bars) in the latter case.

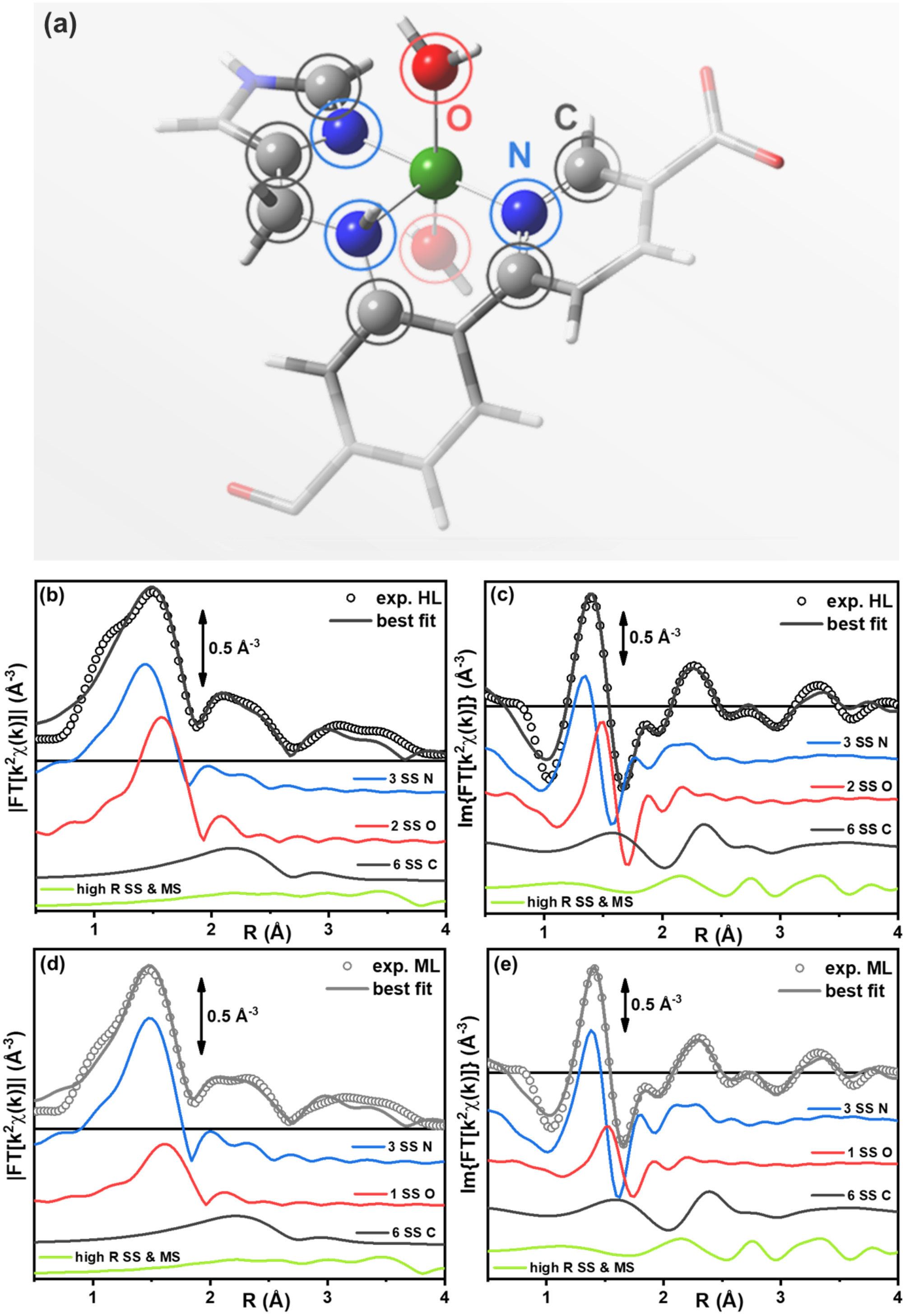

Figure S35. (a) Local structure of Cu sites in UiO-67-[**1**Cu][BF_4]$_2$: Cu (absorber), green; H, white; N, blue; O, red, C, gray; the atoms belonging to the first and second coordination shells of Cu are shown in ball-and-stick mode and highlighted by colored circles, flowing the same color code as in parts (b, c); the second H_2O ligand, preferentially found in the **HL** sample is reported in shadowed color. Magnitude (b, d) and imaginary part (c, e) of the experimental phase-uncorrected FT-EXAFS spectra for the as-prepared UiO-67-[**1**Cu][BF_4]$_2$-**HL** (b,c) and -**ML** (d, e) samples measured RT (circles) compared with correspondent best fit curves (thick lines) obtained using the geometry reported in part (a). The principal contributions to the EXAFS signal are also reported as colored thin lines, using the same color code as in part (a).

Table S9. Results from EXAFS fit for the as prepared Cu-MOF measured at RT using as structural guess the optimized geometry shown in Figure S35. Parameters fixed in the fit are underlined.

**EXAFS Parameters**	**Best fit values As prepared UiO-67-[1Cu][BF_4]$_2$**	
	**HL**	**ML**
R-factor	0.022	0.017
N_{par} (N_{ind})	9 (18)	9 (18)
S_0^2	0.9	0.9
ΔE (eV)	− 3 ± 1	− 2 ± 1
N_N	3	3
<R_N (Å)>	1.92 ± 0.02	1.96 ± 0.01
σ^2_N(Å^2)	0.005 ± 0.003	0.003 ± 0.002
N_O	2	1
<R_O (Å)>	2.02 ± 0.02	2.06 ± 0.02
σ^2_O (Å^2)	0.002 ± 0.002	0.002 ± 0.003
N_C	6	6
<R_C (Å)>	2.88 ± 0.04	2.92 ± 0.04
σ^2_C (Å^2)	0.008 ± 0.005	0.010 ± 0.004
$\alpha_{high\text{-}R}$(Å)	0.04 ± 0.03	0.05 ± 0.02
$SS_{high\text{-}R}$ (Å^2)	0.004 ± 0.003	0.004 ± 0.003

3. UV/Vis Spectroscopy

3.1 MOF Reactivity

The procedure employed during the *in situ* XANES on UiO-67-[**1**Cu][BF_4]$_2$ (see main text) was used to study the DR UV/Vis-NIR spectra during the reaction (Figure S36a). The exposure of UiO-67-[**1**Cu][BF_4]$_2$ to He flow at 150 °C (green *vs.* red spectrum) results in a decrease in the intensity of the band at 15100 cm^{-1} and the growth of a new component at 19300 cm^{-1}. This corresponds to the dehydration described in the main text.

Figure S36a also shows the subsequent reaction of UiO-67-[**1**Cu][BF_4]$_2$ with O_2 in isothermal conditions, resulting in very minor changes of the spectrum. A difference spectrum can be calculated subtracting the red spectrum (after 60 min in He at 150 °C) from the blue one (after 60 min in O_2 at 150 °C) in order to highlight the effect of the O_2 treatment on the electronic transitions (see Figure S36b). It supports a slight increase of Cu (II) species, since the band at 15100 cm^{-1} related to its d-d transitions is slightly decreased in intensity.

As a control, the same treatment was run on UiO-67-**1**, where no substantial changes were observed (see Figure S37).

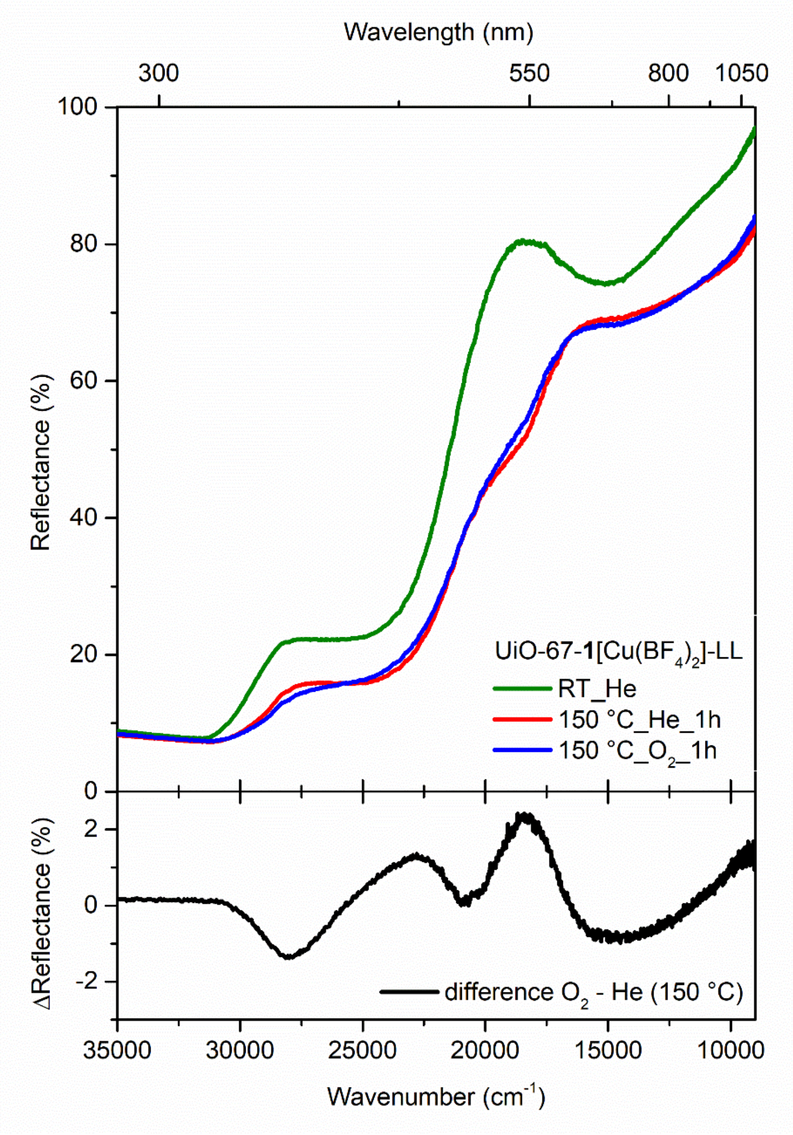

Figure S36. A) DR UV/Vis-NIR spectra of UiO-67-[**1**Cu][BF_4]$_2$-**LL** at RT under He flow (dark green) and activated under He flow up to 150 °C. The red spectrum shows the material after 60 min under He flow at 150 °C, while the blue one after 60 min under O_2 flow at the same temperature. b) Difference spectrum obtained subtracting the red spectrum from the blue one.

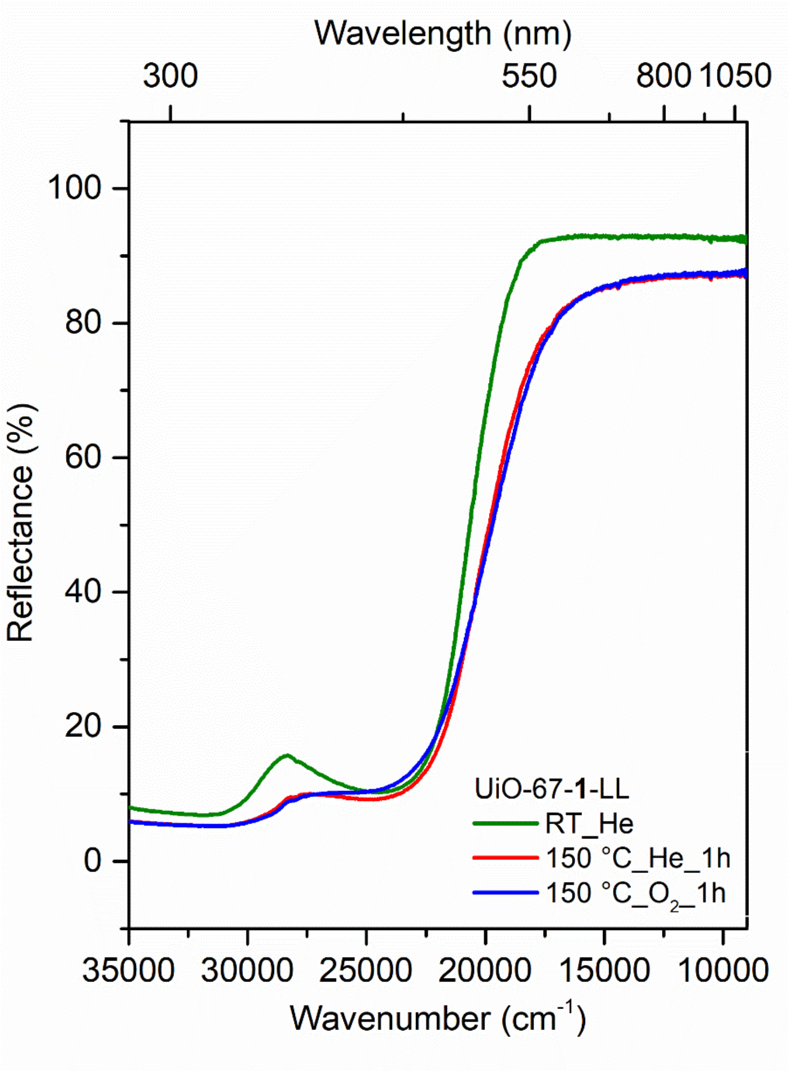

Figure S37. DR UV/Vis-NIR spectra of UiO-67-**1**-**LL** at RT under He flow (dark green). The red spectrum shows the material after 60 min under He flow at 150 °C, while the blue one was obtained after 60 min under O_2 flow at the same temperature.

4. Catalytic testing

4.1 Test and Work-up Procedures

The tridentate complexes [(Me$_2$**1**)$_2$Cu][BF$_4$]**,** [Me$_2$**1**Cu][BF$_4$]$_2$ and the corresponding MOF system UiO-67-[**1**Cu][BF$_4$]$_2$-**ML** were studied for the catalytic oxidation of cyclohexane using H_2O_2 as a co-substrate. The oxidation products were identified by GC-MS as cyclohexanol, cyclohexanone, and cyclohexyl hydroperoxide. Reactivity of these Cu-based systems was compared to corresponding copper tetrafluoroborate salts.

The tests were performed in closed vials at 25 °C using acetonitrile as a solvent. The catalyst amount was scaled such that equal concentration of copper sites was used in all tests. More specifically, the copper concentration was 1.84 mmol/L, cyclohexane concentration 155 mmol/L, and H_2O_2 concentration 1370 mmol/L, in all tests.

Samples were collected at 0.5 h and 4 h intervals. Three work-up steps were applied before GC-MS analysis. First, freshly prepared and activated alumina columns were used to capture the catalyst, and a 1:1 v/v of acetonitrile:diethyl ether mixture was used to rinse the columns and extract the products. When the samples were subsequently analysed by GC-MS without further work-up, a random decomposition pattern of cyclohexyl hydroperoxide into cyclohexanol, cyclohexanone and other species was observed, leading to inaccurate identification and quantification of the products. Therefore, an excess amount of triphenylphosphine was added to the solution before GC-MS injection, to reduce cyclohexyl hydroperoxide into cyclohexanol before being quantified indirectly via GC-MS. This procedure was originally developed by Shul'pin and co-workers and has later been used by several groups (see publication by Shul'pin,[12] and references therein). Finally, a constant amount of cycloheptanone was added to all samples as an internal standard before injection.

In situ NMR testing was performed in CD_3CN in J-Young NMR tubes with a zgesgp pulse sequence that suppresses the water signal by excitation sculpting. The actual ^{1}H resonance of water was determined before every measurement and used in the subsequent water suppression experiment, as a strong shift was observed during the experiment.

4.2 Selectivity Studies

A modified work-up procedure was followed to further investigate the selectivity towards different products. In preliminary studies, acidic activated alumina showed high selectivity to capture cyclohexanol while the same amount of cyclohexanone was recovered. Hence, we utilized the same previous method for testing, but two parallel samples were collected at each interval and different types of columns were used (acidic and neutral alumina). Data from both samples were processed and compared to calculate the actual selectivity of the catalytic systems to each type of oxidation products.

4.3 *Leaching Test of MOF Sample*

Same tests as before were repeated for the MOF sample UiO-67-[**1**Cu][BF_4]$_2$-**ML** at 1.84 mmol/L copper concentration. The tests were performed for 0.5, 4 or 20 h under the same conditions. Subsequently, the vials were centrifuged at 3000 rpm for 20 min and the supernatant was transferred into closed vials. Two samples were collected from each vial right after centrifugation and after a total of 24 h and 48 h of reaction at 25 °C. Samples were processed into neutral alumina columns as before and analyzed via GC-MS. Data from all intervals were compared.

4.4 *Analysis equipment*

GC-MS analysis of liquid samples was performed on an Agilent Technologies 7890B GC system equipped with a 5977B MSD detector, FID detector, and an Automatic Liquid Sampler (ALS). A backflushing system was built in the GC-MS using two identical columns of the type vf-5ms (40 m, 0.15 mm, 0.6 µm) connected sequentially. An injection volume of 1 µL with a 1:10 split ratio was used to analyze samples. The temperature program was as follows: 70 °C initial temperature (hold time: 2 min), followed by an increasing temperature up to 175 °C (rate: 15 °C/min, hold time: 1 min), then increased up to 250 °C (rate: 30 °C/min, hold time: 6 min), and finally the backflushing mode was activated with a reverse flow for 10 min at 300 °C.

4.5 *NMR Analysis of the Digested MOF UiO-67-[1Cu][BF_4]$_2$*

UiO-67-[**1**Cu][BF_4]$_2$ MOF was put under reaction conditions for 24 h, separated, washed extensively with acetonitrile and diethylether, and then digested using 1 M NaOD as follows. 30 mg of MOF sample was added to a 15 mL HDPE falcon tube. 0.8 mL sodium deuteroxide (1.0 M NaOD in D_2O) was added to the MOF, and the tube was agitated using a whirlmixer for 20 seconds. The precipitate was separated from the mother liquor by centrifugation (3000 rpm, 30 minutes). 0.5 mL of the mother liquor was transferred to a clean NMR tube and analyzed using a 400 MHz NMR spectrometer (BRUKER AVIII400). ^{1}H-NMR, ^{13}C-NMR, HSQC, and HMBC spectra were collected and analyzed. The same procedure was followed for the parent MOF and compared with reacted sample.

Table 10. Reactivity parameters of the molecular complexes [(Me_2**1**)$_2$Cu][BF_4] and [Me_2**1**Cu][BF_4]$_2$, and the heterogeneous UiO-67-[**1**Cu][BF_4]$_2$ system for the catalytic oxidation of cyclohexane after 0.5 and 4 h contact time compared to the corresponding copper tetrafluoroborate salts. Copper concentration 1.84 mmol/L, cyclohexane concentration 155 mmol/L, H_2O_2 concentration 1370 mmol/L.

**Catalyst**	**Total turnover to Cy-OOH + Cy=O + Cy-OH**		**Total yield (%)**		**Turnover frequency (h^{-1})**	
	**0.5 h**	**4 h**	**0.5 h**	**4 h**	**0.5 h**	**4 h**
**[($Me_2$1)$_2$Cu][BF_4]**	4.1	25.6	4.8	30.4	8.2	6.4
**[$Me_2$1Cu][BF_4]$_2$**	1.7	9.6	2.0	11.2	3.4	2.4
**UiO-67-[1Cu][BF_4]$_2$-ML**	0.1	0.3	0.1	0.4	0.2	0.1
**Cu^{I}(BF_4)**	0.8	4.5	0.9	5.4	1.5	1.1
**Cu^{II}(BF_4)$_2$**	0.8	4.7	1.0	5.5	1.6	1.2

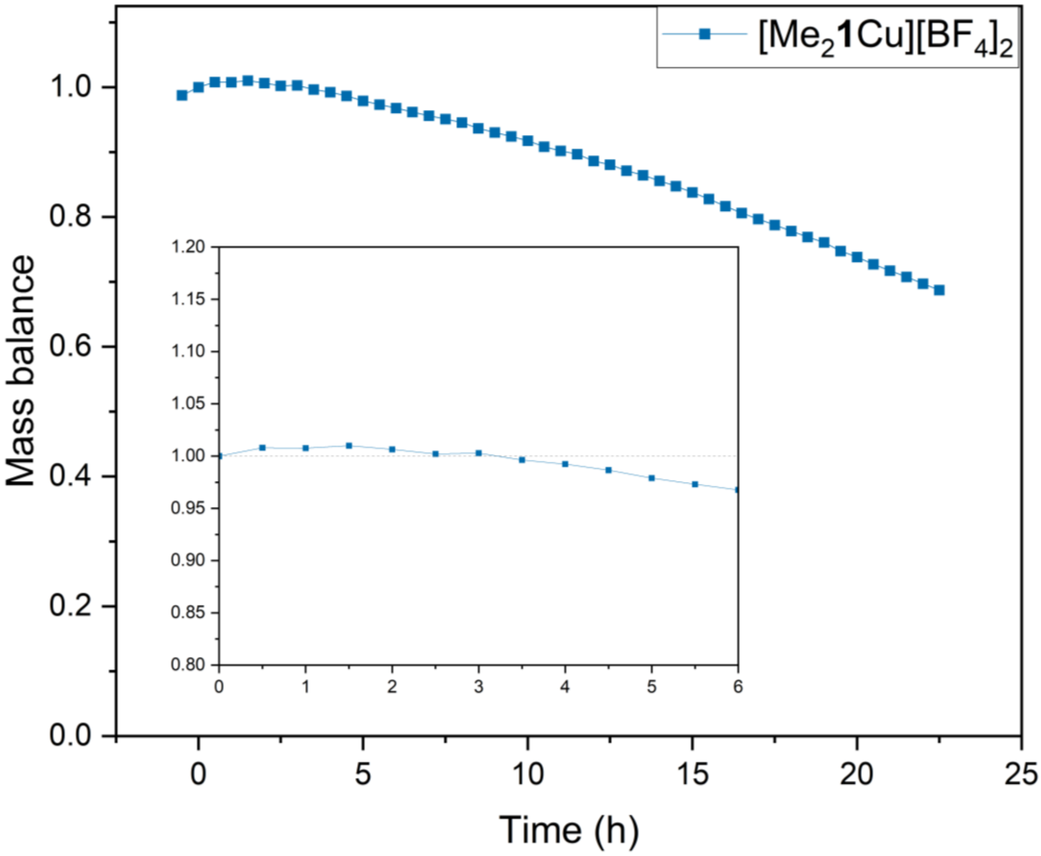

Figure S38. Mass balance of cyclohexane conversion over complex [Me_2**1**Cu][BF_4]$_2$ based on *in situ* NMR testing.

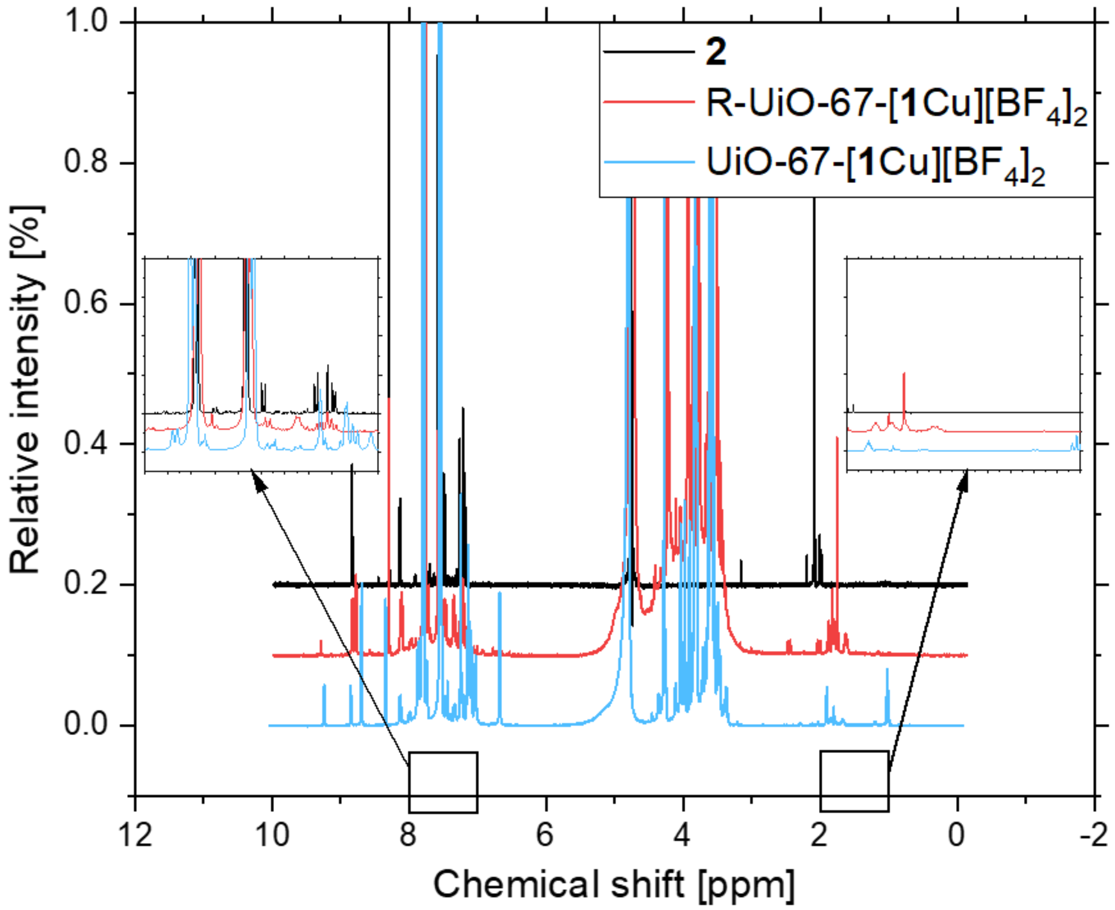

Figure S39. ^{1}H-NMR data of the digested Cu-MOF after catalytic testing R-UiO-67-[1Cu][BF_4]$_2$ (red line), the parent UiO-67-[1Cu][BF_4]$_2$ (light blue line) and compound **2** (methyl ester of the expected hydrolysis product, black line).

5. Bibliography

1 G. M. Sheldrick, *Acta Cryst.*, 2015, **C71**, 3–8.
2 G. M. Sheldrick, *Acta Cryst.*, 2015, **A71**, 3–8.
3 O. V. Dolomanov, L. J. Bourhis, R. J. Gildea, J. a. K. Howard and H. Puschmann, *J. Appl. Crystallogr.*, 2009, **42**, 339–341.
4 F. H. Allen, O. Johnson, G. P. Shields, B. R. Smith and M. Towler, *J. Appl. Crystallogr.*, 2004, **37**, 335–338.
5 P. M. Abdala, O. V. Safonova, G. Wiker, W. van Beek, H. Emerich, J. A. van Bokhoven, J. Sá, J. Szlachetko and M. Nachtegaal, *CHIMIA*, 2012, **66**, 699–699.
6 B. Ravel and M. Newville, *J. Synchrotron Rad.*, 2005, **12**, 537–541.
7 O. Mathon, A. Beteva, J. Borrel, D. Bugnazet, S. Gatla, R. Hino, I. Kantor, T. Mairs, M. Munoz, S. Pasternak, F. Perrin and S. Pascarelli, *J Synchrotron Rad*, 2015, **22**, 1548–1554.
8 K. T. Hylland, S. Øien-Ødegaard and M. Tilset, *Eur. J. Org. Chem.*, 2020, 4208–4226.
9 T. V. W. Janssens, H. Falsig, L. F. Lundegaard, P. N. R. Vennestrøm, S. B. Rasmussen, P. G. Moses, F. Giordanino, E. Borfecchia, K. A. Lomachenko, C. Lamberti, S. Bordiga, A. Godiksen, S. Mossin and P. Beato, *ACS Catal.*, 2015, **5**, 2832–2845.
10 H. Fei and S. M. Cohen, *Chem. Commun.*, 2014, **50**, 4810–4812.
11 D. K. Sannes, S. Øien-Ødegaard, E. Aunan, A. Nova and U. Olsbye, *Chem. Mater.*, , DOI:10.1021/acs.chemmater.2c03744.
12 G. B. Shul’pin, *J. Mol. Catal. A: Chem.*, 2002, **189**, 39–66.
